# Gut microbiota in patients with prostate cancer: a systematic review and meta-analysis

**DOI:** 10.1186/s12885-024-12018-x

**Published:** 2024-02-24

**Authors:** Haotian Huang, Yang Liu, Zhi Wen, Caixia Chen, Chongjian Wang, Hongyuan Li, Xuesong Yang

**Affiliations:** Department of Urology, Afliated Hospital of North Sichuan Medical College, Nanchong, China

**Keywords:** Prostate cancer, Gut microbiota, Microbiome, 16S sequencing, Systematic review and meta-analysis

## Abstract

**Background:**

Increasing evidence indicates that gut microbiota are closely related to prostate cancer. This study aims to assess the gut microbiota composition in patients with prostate cancer compared to healthy participants, thereby advancing understanding of gut microbiota's role in prostate cancer.

**Methods:**

A systematic search was conducted across PubMed, Web of Science, and Embase databases, in accordance with the Preferred Reporting Items for Systematic Reviews and Meta-Analyses (PRISMA) guidelines. The methodological quality of included studies was evaluated using the Newcastle–Ottawa Scale (NOS), and pertinent data were analyzed. The kappa score assessed interrater agreement.

**Results:**

This study encompassed seven research papers, involving 250 prostate cancer patients and 192 controls. The kappa was 0.93. Meta-analysis results showed that alpha-diversity of gut microbiota in prostate cancer patients was significantly lower than in the control group. In terms of gut microbiota abundance, the ratio of *Proteobacteria*, *Bacteroidia*, *Clostridia*, *Bacteroidales*, *Clostridiales*, *Prevotellaceae*, *Lachnospiraceae*, *Prevotella*, *Escherichia*-*Shigella*, *Faecalibacterium*, and *Bacteroides* was higher in prostate cancer patients. Conversely, the abundance ratio of *Actinobacteria*, *Bacteroidetes*, *Firmicutes*, *Selenomonadales*, *Veillonella*, and *Megasphaera* was higher in the control group.

**Conclusion:**

Our study reveals differences in alpha-diversity and abundance of gut microbiota between patients with prostate cancer and controls, indicating gut microbiota dysbiosis in those with prostate cancer. However, given the limited quality and quantity of selected studies, further research is necessary to validate these findings.

**Supplementary Information:**

The online version contains supplementary material available at 10.1186/s12885-024-12018-x.

## Background

Prostate cancer (PCa) is the most prevalent malignant tumor in males, particularly in the United States [1], significantly impacting public health. In 2022, PCa constituted approximately 27% of newly diagnosed male cancer cases, with its mortality rate ranking second among male cancers [2]. The incidence of PCa is also rapidly increasing in many Asian countries [3]. Research has indicated potential influences of various factors on PCa development, including genetics, race, age, local inflammation, and lifestyle habits [4–7]. However, the definitive impact of these factors on PCa pathogenesis remains unconfirmed. Recent studies have highlighted an increasing association between human diseases and microbiota, notably the gut microbiota (GM). Consequently, microbial factors, such as urinary and gut microbiota, are attracting significant interest in their impact on health [8, 9].

The term 'microbiota' denotes the collection of microorganisms residing in a specific biological environment, including bacteria, viruses, parasites, and fungi [10]. The mammalian gastrointestinal tract hosts a complex community of trillions of symbiotic entities, such as bacteria, fungi, archaea, and viruses, collectively known as the GM [11]. Research has linked the GM to various conditions, including diabetes, Alzheimer's disease, and ulcerative colitis [12–15]. Advances in next-generation sequencing technologies have greatly improved our understanding of the GM's composition, for example, through the sequencing of the 16S rRNA gene or its amplicons, based on the variability of small subunit ribosomal RNA sequences [16, 17]. This has enabled deeper exploration into the GM's relationship with diseases.

The prostate, being relatively distant from the gut, initially left the impact of gut microbiota (GM) on PCa unclear. However, recent studies have uncovered an association between GM and PCa. In 2018, Golombos et al. analyzed the GM of 20 male subjects, noting a higher prevalence of Bacteriodes massiliensis in PCa patients, although GM diversity appeared similar when comparing PCa patients with healthy controls [18]. In 2022, Fernandes et al. observed differences in the relative abundance of phylum-level bacteria between PCa patients and healthy individuals [19]. These studies suggest a significant link between GM and PCa, utilizing GM sequencing to analyze PCa patient samples.

Nevertheless, due to varying sample sizes and individual differences, the specific characteristics of GM in PCa patients remain ambiguous. To address this, our meta-analysis was conducted to examine changes in GM composition in PCa patients. This aims to discern GM's role in the etiology and progression of PCa and to explore new preventive and diagnostic methods.

## Methods

This systematic review and meta-analysis adhered to the Preferred Reporting Items for Systematic Reviews and Meta-Analyses (PRISMA) guidelines and was prospectively registered with PROSPERO (CRD 42023476765).

### Search strategy

We systematically retrieved relevant studies from the PubMed, EMBASE, and Web of Science databases from their inception until September 2023. Our search strategy was based on the PICOS principle: (P) Population: prostate cancer patients; (C) Comparison: healthy controls; (O) Outcome: diversity and abundance of gut microbiota; (S) Study Design: prospective studies, case–control studies, or cohort studies. The details of our search terms and strategy are presented in Additional file 1: Table S1 (using PubMed as an example).

### Inclusion and exclusion criteria

The inclusion criteria for studies were: (1) prospective studies, cohort studies, or case–control studies; (2) original research comparing the GM of PCa patients with a control cohort; (3) use of 16S rRNA sequencing technology; and (4) studies reporting microbial communities in fecal samples. The exclusion criteria were: (1) studies not on topic; (2) animal experiments, reviews, summaries, conference abstracts, secondary research, and editorials; and (3) studies where microbiota originated from urine, prostatic fluid, or prostate tissuee.

### Study selection

Endnote reference management software was used for managing literature and eliminating duplicate records in our study. Records with 100% similarity were automatically removed, while those with 80–99% similarity were manually reviewed for removal. Two researchers (HH and LY) screened titles and abstracts for initial evaluation and categorization, determining which literature to include or exclude. They then fully read the remaining literature to confirm its relevance. The eliterature selection was independently conducted by these two investigators. Disagreements were resolved by consulting a third researcher.

Additionally, reference lists of included studies, systematic reviews, and reviews on the topic were scrutinized. All related articles were thoroughly read, and relevant articles were identified using the snowball technique.

### Data extraction and quality assessment

Data extraction was independently performed by two researchers, including details such as the first author’s name, publication year, country, participant number, sample collection method, and the alpha-diversity and abundance of GM. Disputes were resolved through discussion with a third researcher. We used kappa score to assess interrater agreements. A kappa score ≤ 0.2 was considered a poor agreement, 0.21- 0.40 as fair agreement, 0.41–0.60 as moderate agreement, 0.61–0.80 as good and 0.81–1.00 as very good agreement [20].

For quality assessment, we used the Newcastle–Ottawa Scale (NOS) [21]. This tool, designed for observational studies, comprises eight components assessing the study's selectiveness, comparability of the exposed group, and outcome clarity. The total score is 9 points, with studies scoring 6 or above considered high-quality. Studies scoring below 6 were deemed low-quality and excluded from our analysis.

### Outcome measures

The primary outcome assessed was the variation in alpha-diversity of gut microbiota between prostate cancer patients and the control group. "Alpha-diversity" is an evaluation of microbial diversity, which may include species richness, evenness of abundance, or both. Indexes such as Shannon and Simpson were utilized to assess alpha-diversity, while the Chao1 index and the count of Observed species or Operational Taxonomic Units (OTUs) estimated microbial richness. The secondary outcome evaluated the relative abundance of various taxa within the microbiota across studies, encompassing taxonomic categories like phylum, class, order, family, and genus.

### Data analysis

A meta-analysis was conducted using StataMP 15.0 to assess both the primary outcome (alpha-diversity of GM) and the secondary outcome (relative abundance of various taxa). For continuous indicators, such as microbial alpha-diversity and abundance, we compiled and analyzed the overall mean, standard deviation (SD), and standard error (SE). SE was calculated using the formula SE = √(r*[1-r]/n) if not provided in the studies. For PCa and control groups with multiple subgroups, subgroup data were combined. The combined effect size (ES) was calculated using StataMP 15.0. Heterogeneity was quantitatively analyzed using I^2^ [22]. A random-effects model was applied if I2 > 50%; a fixed-effects model was used if I2 < 50%. Sensitivity analysis was conducted by sequentially excluding individual studies to confirm the stability and reliability of our results. Publication bias was assessed using Begg's rank correlation test and Egger's linear regression test, with *p* < 0.05 considered statistically significant [23].

## Results

### Study selection,characteristics and quality of included studies

**Fig. 1:**
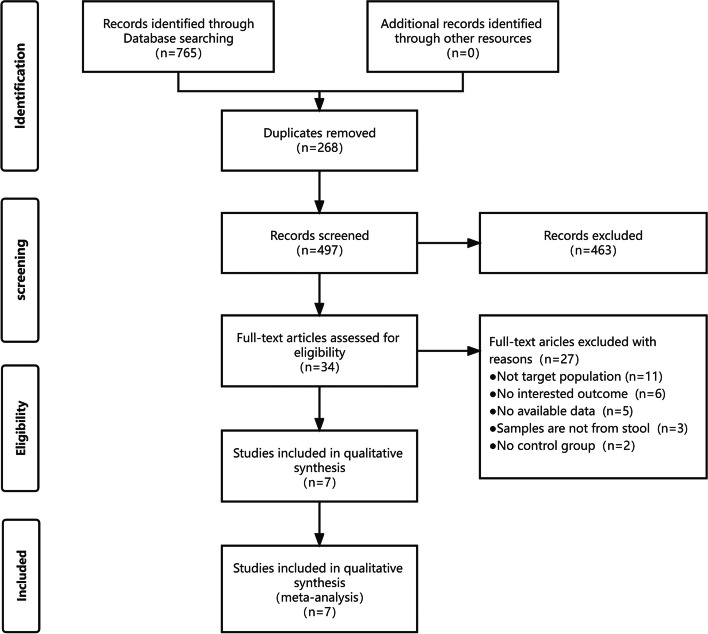
PRISMA fow diagram for the systematic review

From an initial retrieval of 765 articles from the database, 268 duplicates were identified and removed. Reviewing the titles and abstracts of the remaining 497 articles resulted in a further exclusion of 463 studies. After full-text assessments of the remaining 34 articles, 27 were excluded due to non-conforming inclusion populations, lack of GM-related data, non-fecal sample origin, or absence of a control group. Consequently, 7 articles were included in this study [24–30], as illustrated in Fig. 1. These articles predominantly originated from China, the United States, Finland, and Israel, encompassing a total of 442 samples (250 PCa patients and 192 controls). All studies employed 16sRNA sequencing, though the amplified regions of the 16S rRNA gene varied. Three studies targeted the V4 region [26, 29, 30], one the V3-V5 region [24], one the V3-V4 region [25], one the V6 region [27], and one the V1-V2 region [28]. For detailed population data and microbiota information, please see Table 1.

**Table 1:** Characteristics of included studies

Authors	Country	Number of patients	Control group	Age	Method of Sample collection	Microbiota analysis technique	Alpha-diversity metrics	Relative abundance of various taxa				
								**Phylum**	**Class**	**Order**	**Family**	**Genus**
Alanee et al. 2019 [24]	USA	30 (14 PCa vs. 16 controls)	Cancer-free	NA	Rectal swab	16 S rRNA gene sequencing (V3-V5 region)	Not significantly different (Shannon index, *P* > 0.05)	Actinobacteria, Bacteroidetes, Firmicutes, and Proteobacteria	Actinobacteria, Bacteroidia, Bacilli, Clostridia, Negativicutes, and Gammaproteobacteria	Bacteroidales, Lactobacillales, Clostridiales, Selenomonadales, Enterobacteriales, and Actinomycetales	Corynebacteriaceae, Prevotellaceae, Streptococcaceae, Ruminococcaceae, Veillonellaceae, Enterobacteriaceae, Bacteroidaceae, and Moraxellaceae	Prevotella, Escherichia -Shigella, Faecalibacterium, Bacteroides, Veillonella, Streptococcus, and Acinetobacter
Zhong et al. 2022 [25]	China	35 (15 with nmPCa/mPCa vs. 20 controls)	BPH	BPH: 70.5 (IQR, 63–76.75) nmPca: 69.5 (IQR, 66.5–75) mPca: 68 (IQR, 65–74)	Stool	16 S rRNA gene sequencing (V3-V4 region)	Not significantly different (Shannon, Simpson index, *P* > 0.05)	Actinobacteria, Bacteroidetes, Cyanobacteria, Firmicutes, Proteobacteria, Verrucomicrobia, Fusobacteria, and Synergistetes	Actinobacteria, Bacteroidia, Bacilli, Clostridia, Erysipelotrichia, Negativicutes, Gammaproteobacteria, Coriobacteriia, Deltaproteobacteria, and Verrucomicrobiae	Bacteroidales, Lactobacillales, Clostridiales, Selenomonadales, Enterobacteriales, Bifidobacteriales, and Coriobacteriales	Prevotellaceae, Lachnospiraceae, Ruminococcaceae, Acidaminococcaceae, Veillonellaceae, Enterobacteriaceae, Bacteroidaceae, and Bifidobacteriaceae	Prevotella, Escherichia -Shigella, Faecalibacterium, Bacteroides, and Megasphaera
Smith et al. 2021 [26]	USA	16 (8 PCa vs. 8 controls)	Cancer-free	NA	Stool	16 S rRNA gene sequencing (V4 region)	Significantly different (Chao1, Observed Species index, *P* < 0.05); Shannon index was not significantly different (*P* > 0.05)	Actinobacteria, Bacteroidetes, Firmicutes, Proteobacteria, Fusobacteria, Synergistetes, and Spirochaetes	Actinobacteria, Bacteroidia, Bacilli, Clostridia, Erysipelotrichia, Gammaproteobacteria, Coriobacteriia, Fusobacteria, Alphaproteobacteria, Betaproteobacteria, Epsilonproteobacteria, Synergistia, and Spirochaetes	Bacteroidales, Lactobacillales, Clostridiales, Actinomycetales, Bifidobacteriales, and Coriobacteriales	Prevotellaceae, Streptococcaceae, Lachnospiraceae, Ruminococcaceae, Erysipelotrichaceae, Veillonellaceae, Burkholderiaceae, Moraxellaceae, Bifidobacteriaceae, and Actinomycetaceae	Prevotella, Faecalibacterium, Veillonella, Streptococcus, Acinetobacter, and Megasphaera
Sfanos et al. 2018 [27]	USA	30 (21 with LC/ BR/ mHSPC/ mCPRC vs. 9 controls)	Cancer-free, BPH	BPH: 68 (52–80) Cancer-free: 70 (64–78) LC: 60.1 (53–71) BR: 64.4 (54–72) mHSPC: 58 (51–65) mCPRC: 74 (64–85)	Rectal swab	16 S rRNA gene sequencing (V6 region)	Significantly different (Chao1,Observed species index, *P* < 0.05); Not significantly different (Shannon, Simpson index, *P* > 0.05)	NA	NA	NA	Corynebacteriaceae, Prevotellaceae, Streptococcaceae, Lachnospiraceae, Ruminococcaceae, Erysipelotrichaceae, Bacteroidaceae, Bifidobacteriaceae, and Actinomycetaceae	NA
Liss et al. 2018 [28]	USA	105 (64 PCa vs. 41 controls)	Cancer-free	Cancer-free: 65 (IQR, 60–68.5) Pca: 66.5 (IQR, 62–70)	Rectal swab	16 S rRNA gene sequencing (V1-V2 region)	NA	Actinobacteria, Bacteroidetes, Firmicutes, Proteobacteria, Verrucomicrobia, Fusobacteria, Synergistetes, and Spirochaetes	Actinobacteria, Bacteroidia, Bacilli, Clostridia, Erysipelotrichia, Negativicutes, Gammaproteobacteria, Deltaproteobacteria, Fusobacteria, Alphaproteobacteria, Betaproteobacteria, Epsilonproteobacteria, Synergistia, and Spirochaetes	NA	NA	NA
Kalinen et al. 2021 [29]	Finland	181 (108 PCa vs. 73 controls)	Cancer-free	Cancer-free: 62 (IQR, 55–67) Pca: 68 (IQR, 62–72)	Rectal swab	16 S rRNA gene sequencing (V4 region)	Not significantly different (Chao1, Shannon index, *P* > 0.05)	NA	NA	NA	Prevotellaceae, Lachnospiraceae, Ruminococcaceae, Acidaminococcaceae, Veillonellaceae, Burkholderiaceae, Enterobacteriaceae, and Bacteroidaceae	NA
Katz et al. 2022 [30]	Israel	45 (20 PCa vs. 25 controls)	BPH	NA	Rectal swab	16 S rRNA gene sequencing (V4 region)	Not significantly different (Evenness index, *P* > 0.05)	Actinobacteria, Bacteroidetes, Cyanobacteria, Firmicutes, Proteobacteria, Verrucomicrobia, Fusobacteria, and Synergistetes	NA	NA	NA	NA

*BPH* Benign prostate hyperplasia, *PCa* Prostate cancer, *nmPCa* Non-metastatic prostate cancer, *mPCa* Metastatic prostate cancer, *LC* Localized cancer; *BR* Biochemical recurrence, *mHSPC* Metastatic hormone-sensitive prostate cancer, *mCPRC* Metastatic castration-resistant prostate cancer, *NA* Not applicable

The Kappa’s score for data extraction was 0.93, which demonstrated “very good” interrater agreement. All 7 articles scored 6 or above on the NOS, signifying their high quality (Table 2).

**Table 2:** Quality assessment of studies included for the meta-analysis

Study (year)	Quality assessment criteria			
	Selection (4)	Comparability (2)	Outcome (3)	Quality score
Alanee et al. 2019 [24]	★★	★★	★★★	7
Kalinen et al. 2021 [29]	★★★	★★	★★	7
Katz et al. 2022 [30]	★★	★★	★★	6
Liss et al. 2018 [28]	★★	★★	★★★	7
Sfanos et al. 2018 [27]	★★★	★★	★★★	8
Smith et al. 2021 [26]	★★	★★	★★★	7
Zhong et al. 2022 [25]	★★★	★★	★★★	8

The quality of the studies was evaluated using the Newcastle–Ottawa Quality Assessment Scale for observational studies. ★★ 2 points. ★★★ 3 points. ★★★★ 4 points

### Alpha diversity of GM

**Fig. 2:**
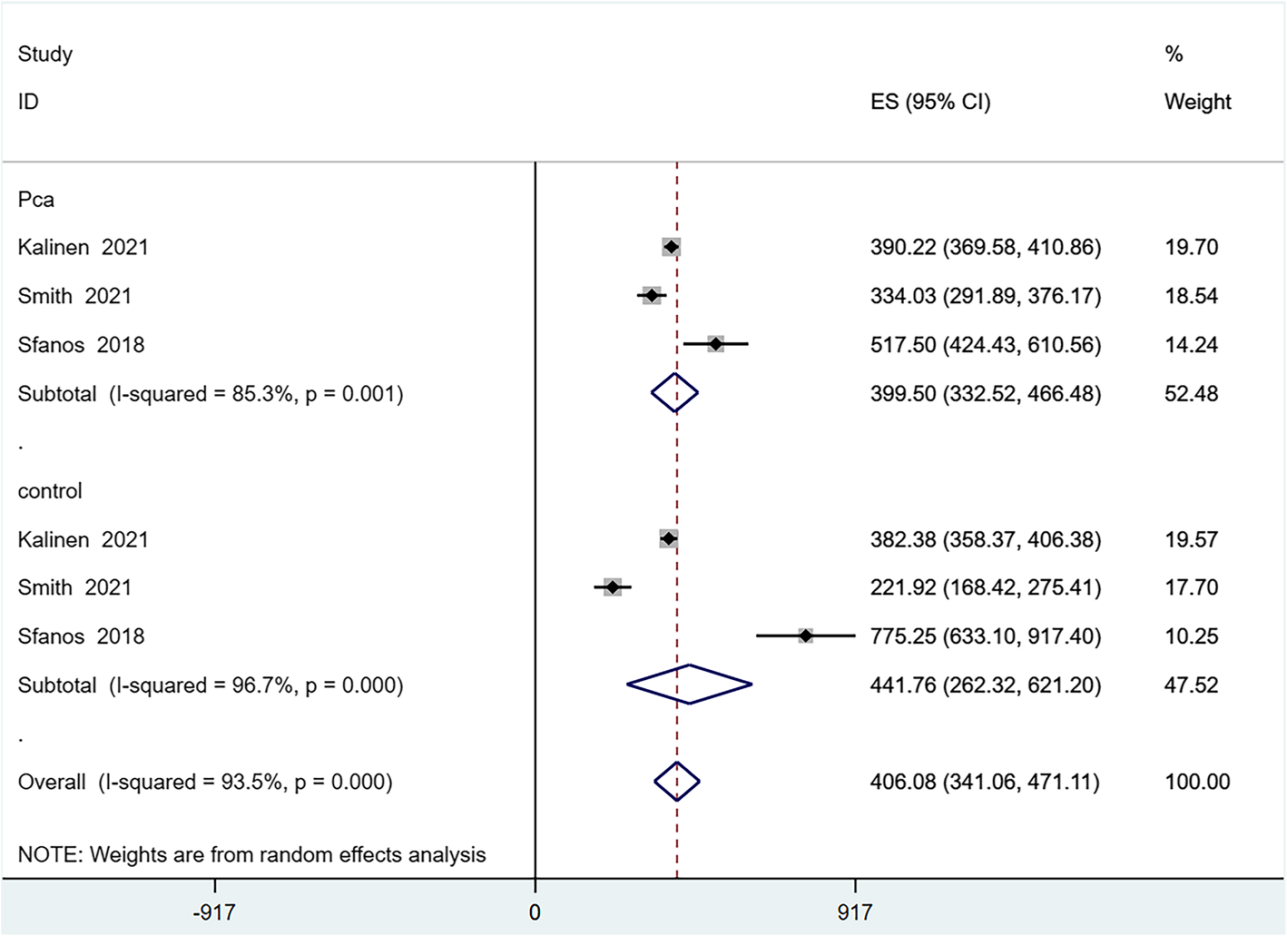
Forest plot of alpha-diversity in Chao1 index

**Fig. 3:**
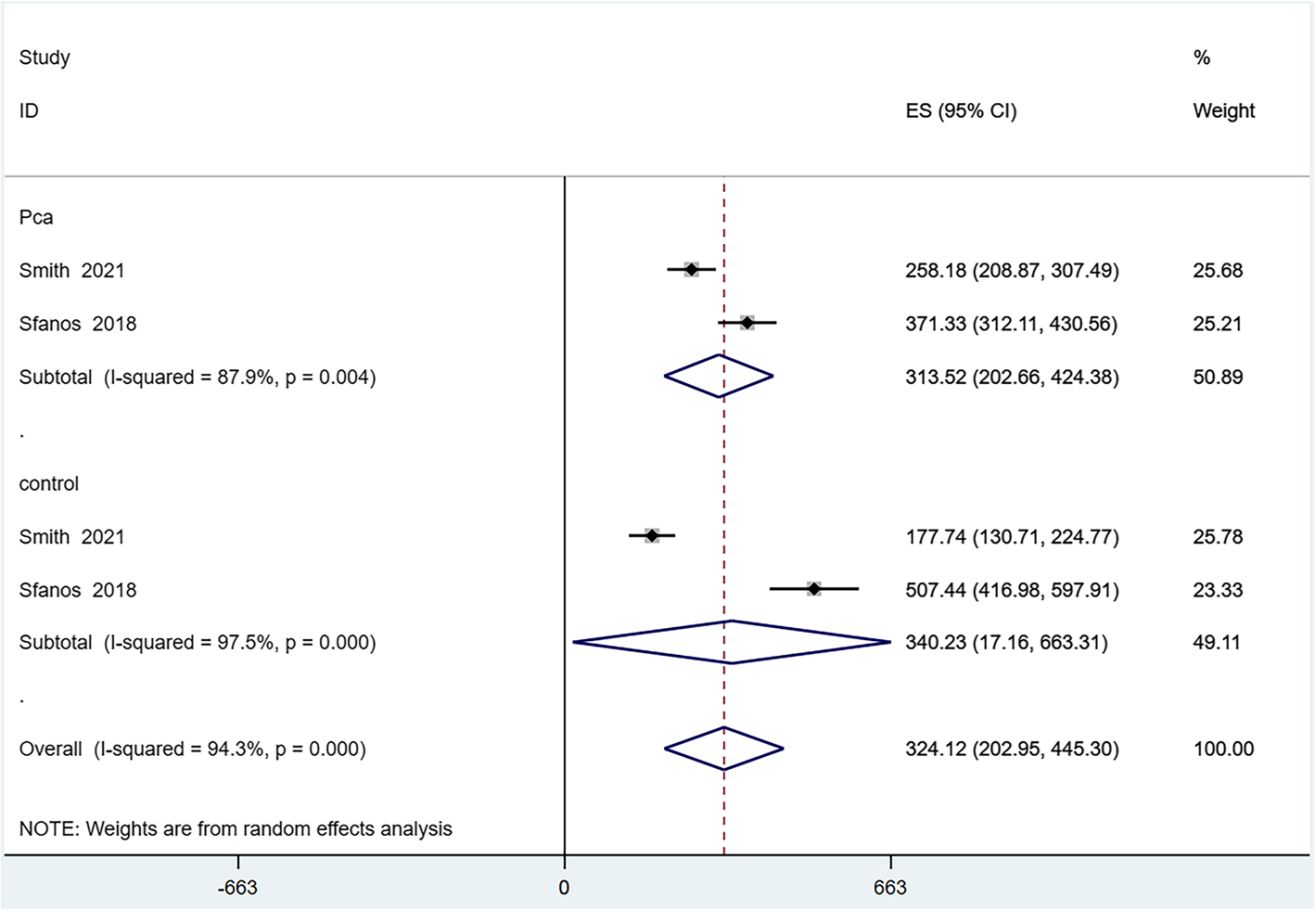
Forest plot of alpha-diversity in Observed Species index

**Fig. 4:**
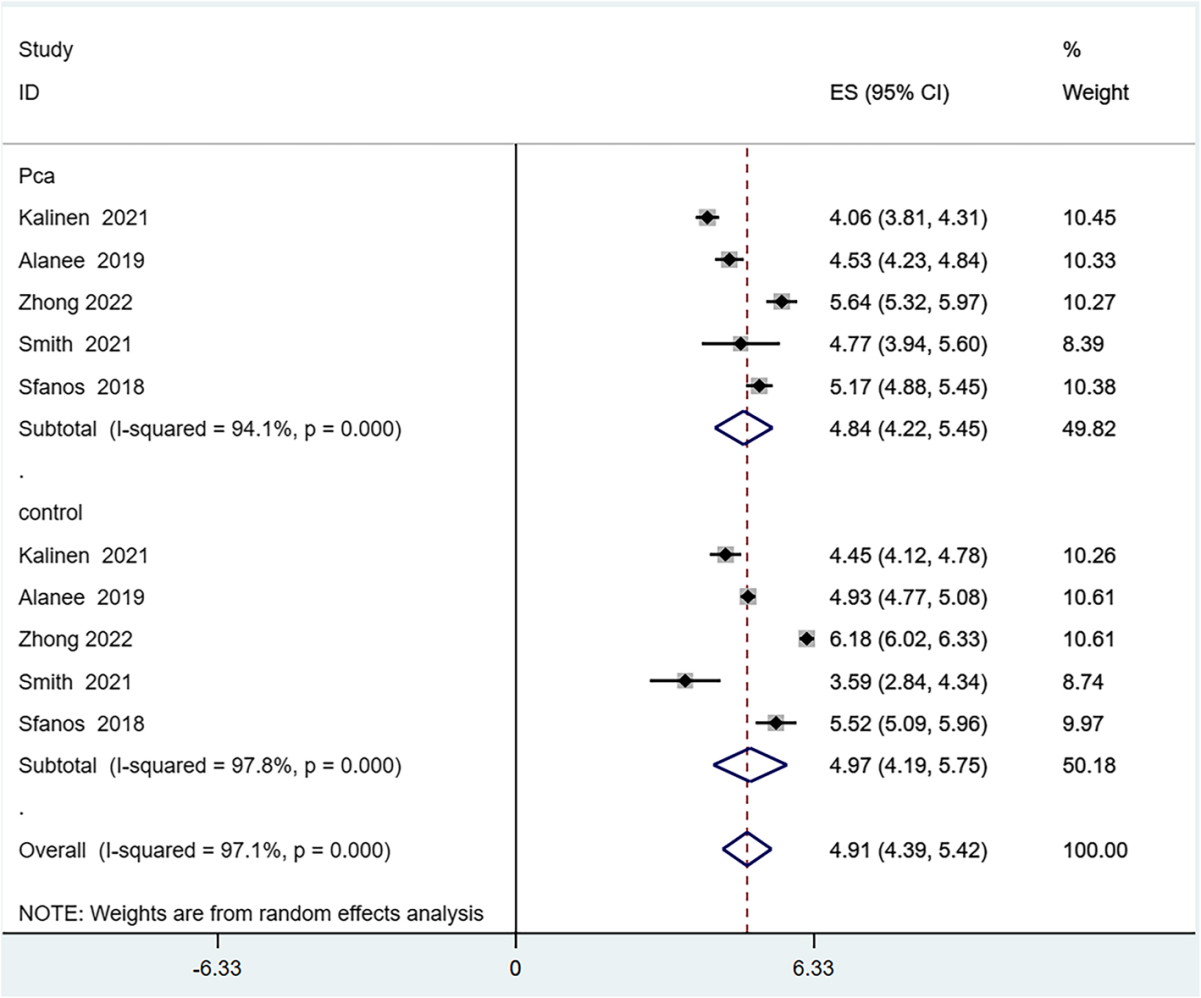
Forest plot of alpha-diversity in Shannon index

**Fig. 5:**
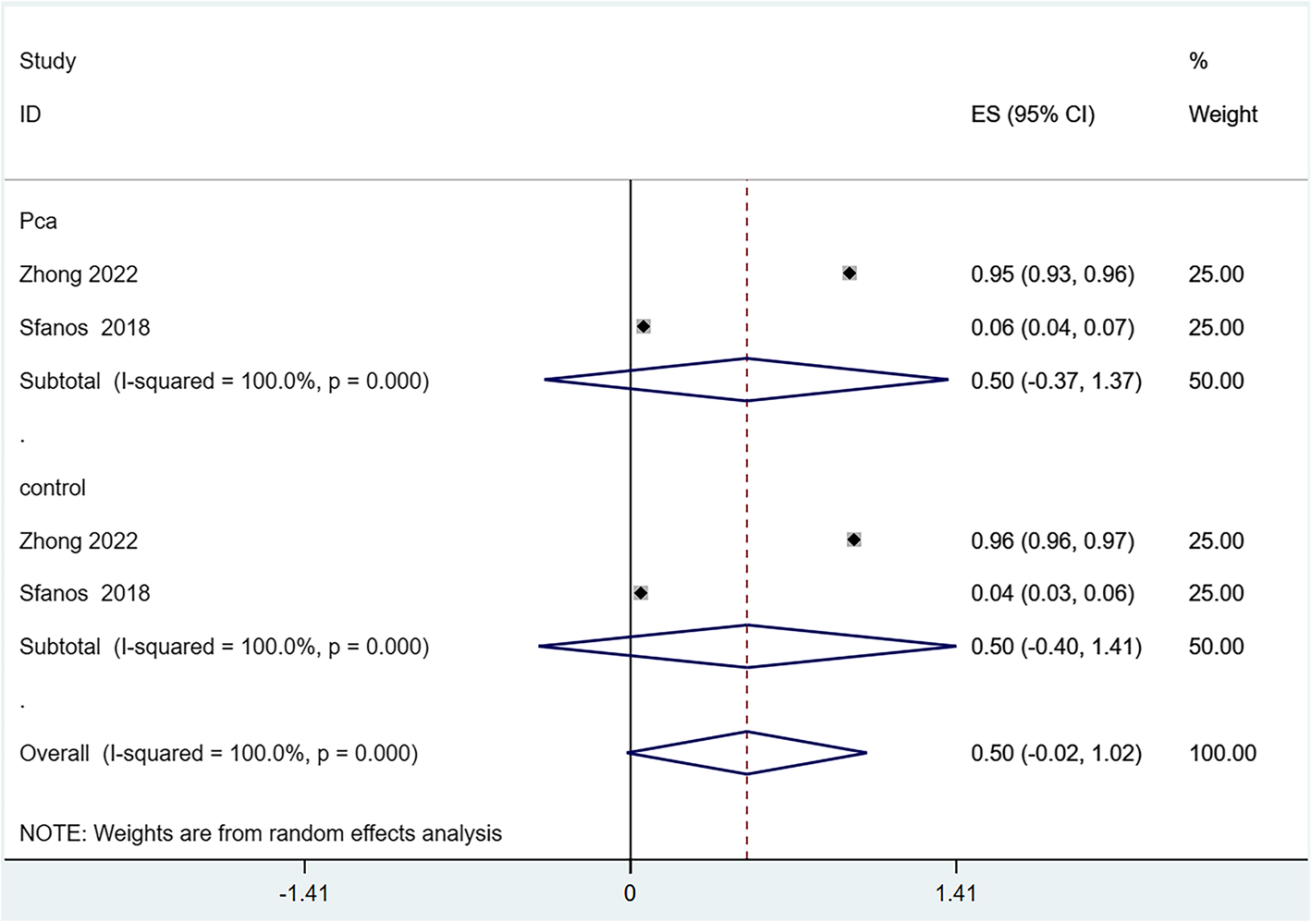
Forest plot of alpha-diversity in Simpson index

Six articles reported on the alpha-diversity of the GM [24–27, 29, 30], However, the Evenness index, reported in only one article [30], was excluded from the quantitative analysis due to insufficient data. We analyzed the alpha-diversity of the PCa population and the control group, including the Chao1, Observed Species, Shannon, and Simpson indexes. The results showed reduced diversity in PCa patients compared to controls, as evidenced by lower scores in the Chao1 (Fig. 2), Observed Species (Fig. 3), and Shannon (Fig. 4) indexes. However, there was no significant difference in the Simpson index diversity between PCa patients and controls (Fig. 5). Due to significant heterogeneity, a sensitivity analysis was conducted (Additional file 2: Figure S1-4). The results' stability was confirmed by this sensitivity analysis, indicating that excluding any single study did not significantly alter the overall effect size.

### Bacterial phylum

Five studies reported data on the relative abundance of bacteria at the phylum level [24–26, 28, 30]. Forest plots showed that the GM of the control group had higher proportions of *Actinobacteria*, *Bacteroidetes*, and *Firmicutes* compared to the PCa group. In contrast, the relative abundance of *Proteobacteria* was higher in PCa patients. No statistically significant differences were observed in the relative abundance of *Cyanobacteria*, V*errucomicrobia*, *Fusobacteria*, *Synergistetes*, and *Spirochaetes* between the PCa and control groups (Additional file 3: Figure S5-13). Due to significant heterogeneity in some bacteria, further sensitivity analysis was conducted (Additional file 3: Figure S14-15). The stability of the results was reaffirmed by this analysis, showing no significant changes in the overall effect size when any single study was excluded.

### Bacterial class

Four studies reported data on the relative abundance of bacteria at the class level [24–26, 28]. Forest plots revealed that the GM of the control group had higher proportions of *Actinobacteria*, *Negativicutes*, *Betaproteobacteria*, and *Epsilonproteobacteria* compared to the PCa group. However, in the PCa group, the relative abundance of *Bacteroidia*, *Clostridia*, *Gammaproteobacteria*, and *Coriobacteriia* exceeded that of the control group. No statistically significant differences were observed in the relative abundance of *Bacilli*, *Erysipelotrichia*, *Deltaproteobacteria*, *Verrucomicrobiae*, *Fusobacteria*, *Alphaproteobacteria*, *Synergistia*, and *Spirochaetes* between PCa patients and controls (Additional file 3: Figure S16-31). Significant heterogeneity in some bacteria necessitated a sensitivity analysis (Additional file 3: Figure S32-34). The results' stability was confirmed by this analysis, showing no significant changes in the overall effect size when any single study was excluded.

### Bacterial order

Three studies reported data on the relative abundance of bacteria at the order level [24–26]. The GM of the control group exhibited higher proportions of *Lactobacillales*, *Selenomonadales*, and *Actinomycetales* compared to the PCa group. In contrast, the relative abundance of *Bacteroidales*, *Clostridiales*, *Enterobacteriales*, *Bifidobacteriales*, and *Coriobacteriales* was higher in PCa patients (Additional file 3: Figure S35-42). Due to significant heterogeneity in some bacteria, a sensitivity analysis was conducted (Additional file 3: Figure S43-45). The stability of the results was reaffirmed by this sensitivity analysis, indicating no significant changes in the overall effect size when any single study was excluded.

### Bacterial family

Five studies reported data on the relative abundance of bacteria at the family level [24–27, 29]. Forest plots showed that the GM of the control group had higher proportions of *Corynebacteriaceae*, *Veillonellaceae*, *Bacteroidaceae*, and *Actinomycetaceae* compared to the PCa group. However, the abundance of *Prevotellaceae*, *Lachnospiraceae*, *Ruminococcaceae*, *Erysipelotrichaceae*, *Burkholderiaceae*, and *Bifidobacteriaceae* was greater in the PCa group. There were no statistically significant differences in the relative abundance of *Streptococcaceae*, *Acidaminococcaceae*, and *Enterobacteriaceae* between PCa patients and controls (Additional file 3: Figure S46-58). Significant heterogeneity in some bacteria necessitated sensitivity analysis (Additional file 3: Figure S59-60). The results' stability was confirmed by this analysis, showing no significant changes in the overall effect size when any single study was excluded.

### Bacterial genus

Three studies have reported data on the relative abundance of bacteria at the genus level [24–26]. Forest plots indicated that the GM of the control group had higher proportions of *Veillonella* and *Megasphaera* compared to the PCa group. In contrast, the abundance of *Prevotella*, *Escherichia-Shigella*, *Faecalibacterium*, and *Bacteroides* was higher in PCa patients. No statistically significant differences in the abundance of *Streptococcus* were observed between PCa patients and controls (Additional file 3: Figure S61-67).

### Publication bias

A risk of bias assessment was conducted for each article included in our study. Based on Begg's correlation test and Egger's regression test, there was no statistically significant evidence of bias in the alpha-diversity of GM (Additional file 4: Table S2). However, for the relative abundance of GM, publication bias was identified in certain bacteria, while no apparent bias was detected in others (Additional file 5: Table S3-7).

## Discussion

Our comprehensive review represents the first meta-analysis examining gut microbiota composition in prostate cancer (PCa) patients. We observed notable variations in the composition of GM between PCa patients and non-PCa individuals. Our results indicated a decline in alpha-diversity of GM in PCa patients compared to the control group. Additionally, significant differences in bacterial relative abundance were evident at the phylum, class, order, family, and genus levels. Specifically, at the phylum level, a higher proportion of *Proteobacteria* was observed in PCa patients, while the proportions of *Actinobacteria*, *Bacteroidetes*, and *Firmicutes* were comparatively lower. At the genus level, increased abundance of *Prevotella*, *Escherichia-Shigella*, *Faecalibacterium*, and *Bacteroides* was noted in PCa patients, with a decreased abundance of *Veillonella* and *Megasphaera*.

Dysbiosis in the gut is defined as any alteration (increase or decrease) in GM that adversely affects the health of the host organism. Several studies suggest that the diversity of GM is increasingly recognized as a crucial factor in host health. Concurrently, a decrease in microbial diversity has been associated with various gastrointestinal and systemic diseases [31, 32]. Thus, GM is considered a regulatory factor in human health [31]. This finding aligns with our research, where a declining trend in gut microbiota was observed in PCa patients. Our studies facilitate exploration into the correlation between PCa and GM, but do not establish a causal relationship. The following factors may contribute to the decrease in gut microbiota α-diversity.

Changes in estrogen levels in humans may contribute to the decline in gut microbial alpha-diversity in patients diagnosed with PCa. Barrett-Connor et al. suggested a potential link between increased estrogen levels in the body and an increased risk associated with the prostate [33]. Thus, estrogen is considered a potential factor influencing the onset and progression of PCa [34]. Estrogen can indirectly suppress androgens by inhibiting the hypothalamic luteinizing hormone-releasing hormone (LHRH), reducing the stimulation of the pituitary gland to secrete luteinizing hormone (LH) and thereby constraining PCa progression. Some gut bacteria can metabolize and produce estrogen, known as the estrobolome, affecting the body's estrogen levels [35]. Normally, conjugated estrogen (glucuronide) produced in the liver cannot bind with estrogen receptors (ER). Gut microbiota can produce beta-glucuronidase to catalyze estrogen from a conjugated form to a dissociated form, which is closely related to human health. Dysbiosis of gut microbiota can impair this process, leading to decreased deconjugation and circulating estrogens, potentially linked with cancer emergence. Furthermore, estrogen might play a role in the progression of PCa, possibly via pathways such as genetic mutation, DNA damage, or chronic inflammation [36].

The implementation of Androgen Deprivation Therapy (ADT) in patients diagnosed with PCa might be linked to a decrease in the alpha-diversity of GM. ADT, a standard treatment for PCa, aims to control disease progression by suppressing androgen production. Matsushita et al. identified a potential positive correlation between serum testosterone levels and the prevalence of *Firmicutes* [37]. A study involving PCa patients who underwent short-term, medium-term, and long-term ADT found that those receiving long-term ADT had significantly lower GM diversity compared to the other groups. At the phylum level, the abundance of Firmicutes and Bacteroidetes was higher in the long-term ADT group than in the other two subgroups [38]. Additionally, Sfanos' research, which analyzed the feces of PCa patients undergoing androgen deprivation therapy (ADT), noted an enrichment of bacteria capable of steroid biosynthesis, such as *muciniphila*, *Ruminococcaceae*, or *Lachnospiraceae*, in the GM of these patients. Gut bacteria can also produce androgens from corticosteroids. These studies suggest that GM undergoes changes due to androgen deprivation and serves as a source of androgenic steroids, potentially contributing to resistance against ADT. This aligns with our findings, where *Ruminococcaceae* and *Lachnospiraceae* are proportionally higher in PCa patients. However, as various bacteria can perform steroid synthesis, further research is needed to identify specific androgenic steroid biosynthetic pathways activated within bacteria [39]. Therefore, the decline in GM diversity may be attributed to changes in testosterone levels [40].

Long-term intake of a high-fat diet (HFD) may also contribute to a decrease in the alpha-diversity of GM in patients with PCa. The composition of GM is influenced by various factors, including lifestyle habits, diet, illness conditions, and drug usage, with dietary factors having a particularly significant impact [41]. The consumption of HFD, dairy products, and processed meats has been confirmed as risk factors for prostate cancer [42, 43]. A study using a prostate-specific Pten knockout mouse model suggests that a high-fat diet (HFD) promotes prostate cancer growth compared to a control diet, with the effects of the control diet being negated by administering broad-spectrum antibiotics [44]. Short-chain fatty acids (SCFA) produced by GM can signal through IGF1 on prostate epithelial cells, activating MAPK and PI3K signaling pathways and stimulating prostate tumor growth. Additionally, SCFA produced by gut bacteria may mitigate inflammation by regulating cytokine production (such as IL-10) and promoting regulatory T cell expansion, though the specific mechanisms are not fully understood. Recent research indicates that HFD consumption increases the abundance of anaerobic bacteria and Bacteroides in the gut. HFD can alter GM, increasing the translocation of Gram-negative bacteria into the bloodstream and mesenteric fat tissue through the intestinal mucosa, leading to inflammation [45]. HFD may also compromise the gut barrier, enhance intestinal permeability, and allow various intestinal metabolites or bacterial components to enter the host's circulation, triggering an inflammatory response. This inflammatory response is a crucial factor in HFD-induced prostate cancer growth, with HFD potentially leading to increased IL-6 expression in prostate tissue and triggering prostate cancer [46].

Quantitatively analyzed at the phylum level, the GM of PCa and control populations exhibited differences, particularly in *Proteobacteria*, *Actinobacteria*, *Bacteroidetes*, and *Firmicutes*. The equilibrium of GM is primarily maintained by these phyla [47], with *Bacteroidetes* and *Firmicutes* typically dominating the balance. A reduction in these bacteria often indicates gut dysbiosis, contributing to disease [48], which aligns with our research findings. Additionally, an increased abundance of *Proteobacteria* is considered indicative of GM dysbiosis. While a temporary rise in Proteobacteria in a healthy state may not cause clinical symptoms [48, 49], a long-term overabundance might reflect microbiota dysbiosis or a diseased state [48]. The specific relationship between *Proteobacteria* and PCa, however, remains unclear and warrants further investigation to explore this connection.

Quantitatively analyzed at the genus level, the gut microbiota (GM) of PCa and control populations show differences, particularly in *Prevotella*, *Escherichia-Shigella*, *Faecalibacterium*, *Bacteroides*, *Veillonella*, and *Megasphaera*. Among these, *Faecalibacterium* is a core genus in the human gut. Research indicates that *Faecalibacterium* can stimulate the NF-KB pathway and elevate the expression of multiple pro-inflammatory cytokine genes, potentially driving the progression of colorectal cancer [50]. While a direct link between *Faecalibacterium* and PCa has not been established, considering the gut inflammation response as a risk factor for PCa [46], a connection is plausible. Studies have shown that the abundance of *Prevotella* is high in the GM of patients with colorectal cancer [51]. Interestingly, Prevotella is also abundant in the gut of PCa patients, suggesting a possible connection. However, the specifics of this relationship and its underlying mechanisms remain to be explored, necessitating further research. Although we have conducted a thorough analysis at the phylum and genus levels, the role of GM at the order, class, and family levels in relation to PCa remains unclear. Future studies are required to explore these aspects and deepen our understanding of GM's role in PCa.

Additionally, dietary habits, medical procedures, race, geographic location, and other factors may contribute to the observed differences in diversity and abundance of GM between the PCa population and the control group. In terms of diet, Western-style diets are often associated with an increased risk of PCa compared to Chinese cuisine. However, current research yields inconsistent findings regarding whether the Western-style diet affects PCa risk through the mediation of GM, or through other factors such as metabolism or inflammation in prostate tissue [52, 53]. Dietary nutrients, including fats, proteins, carbohydrates, vitamins (such as A, D, and E), and polyphenols, may also play a role in preventing PCa by influencing GM, though their specific mechanisms are not yet clear. Geographic variations also influence GM composition; for example, the gut microbiota in Japan exhibits a more abundant *Actinobacteria* phylum [54]. In terms of race, the participants in Alanee's studies were Caucasians [24], while those in Zhong's studies were Asians [25]. The diversity of subjects may impact the results, underscoring the need for more research to examine the influence of various factors on GM composition.

Given the presence of treatment-resistant cases in current PCa therapies, the GM offers a potential avenue for the prevention and treatment of prostate cancer. Understanding the intricate relationship between GM and PCa could lead to novel approaches in managing this disease.

Regarding screening potential, the use of serum PSA screening remains controversial due to modest risk reduction, a high rate of false positives, and questions about cost-efficacy at the population level [55]. Hence, detecting "unfavorable" characteristics in gut microbiota may be incorporated into prostate cancer risk screening. Our research results offer a reference for clinical physicians in this regard.

In terms of therapeutic potential, strategies aimed at transforming the gut microbiota of prostate cancer patients from unfavorable to favorable characteristics may aid in delaying or treating the disease. Various methods, such as fecal microbiota transplantation (FMT), prebiotics, probiotics, or synbiotics, can be used to treat the gut microbiota in prostate cancer. For instance, probiotics have seen wide application in patients with obesity and alcoholic liver disease [56–58]. Our research findings indicate potential bacterial differences between the cancer and control groups, which could guide future researchers in identifying "favorable" or "unfavorable" microbiota. This offers a reference for the development of future microbiota therapies in prostate cancer management.

### Strengths and limitations

Our study exhibited several advantages. We maintained strict inclusion criteria, systematically retrieved all relevant studies that meet our predetermined conditions, and adhered to the PRISMA guidelines for reporting systematic reviews and meta-analyses. Additionally, our research included recent matching cohorts, providing an in-depth examination of the diversity and richness of gut microbiota in patients with PCa.

Despite these strengths, our study faced several limitations. 1. The number of articles included was limited, with only seven studies being available for quantitative analysis. 2. High heterogeneity among the included studies could have influenced the results, a common challenge in observational studies [59], as opposed to randomized controlled trials. 3. The included studies showed significant clinical and methodological heterogeneity, with factors like participant sample size, race, diet, residence, treatment methods, and age impacting GM composition. 4. Variations in DNA extraction methods, sequencing platforms, and sequencing depths used for sequencing the 16S rRNA gene region might have led to inconsistent results. 5. The methods of feces collection, such as stool samples and rectal swabs, also varied, potentially affecting the outcomes. The composition of the control group was not always consistent, and the inclusion of both healthy samples and benign prostatic hyperplasia (BPH) samples might have introduced biases.

Furthermore, our study could not encompass all bacterial strains associated with PCa. While we established a correlation between GM and PCa, this did not definitively imply a causal relationship. Future high-quality studies are required to validate these findings.

## Conclusions

Overall, our meta-analysis findings indicated variances in both the abundance and alpha diversity of GM when comparing PCa patients to the control group.Microbial dysbiosis may be caused by ADT treatment, HFD, and changes in endogenous estrogens. The impact of GM on the pathogenicity of PCa still remained disputed. In the future, the gut microbiota may find broader applications in the screening and treatment of PCa (prostate cancer). However, further foundational and clinical research were required to elucidate this connection.

### Supplementary Information

**Supplementary Material 1.**

**Supplementary Material 2. **

**Supplementary Material 3. **

**Supplementary Material 4. **

**Supplementary Material 5. **

**Supplementary Material 6. **

## Data Availability

The datasets analyzed in this study are potentially available from the corresponding authors upon a reasonable request.

## Abbreviations

PCa: Prostate cancer
GM: Gut microbiota
BPH: Benign prostate hyperplasia
HFD: High-fat diet
LHRH: Luteinizing hormone-releasing hormone
LH: Luteinizing hormone
ADT: Androgen deprivation therapy
