# Supplementary material for: Gut microbiota in patients with prostate cancer: a systematic review and meta-analysis
Source: BMC Cancer. 2024 Feb 24;24:261. doi: 10.1186/s12885-024-12018-x (PMC10893726; doi:10.1186/s12885-024-12018-x)
Supplement: Supplementary file 1 — Supplementary Material 1. [file 12885_2024_12018_MOESM1_ESM.zip › Additional file 1/Table S1. The search strategy.docx]

**Table S1. The search strategy**

| Search | PUBMED |
| --- | --- |
| #1 | Prostatic Neoplasms[MeSH Terms] |
| #2 | ((((((((((((((((Prostate Neoplasms[Title/Abstract]) OR (Neoplasms, Prostate[Title/Abstract])) OR (Neoplasm, Prostate[Title/Abstract])) OR (Prostate Neoplasm[Title/Abstract])) OR (Neoplasms, Prostatic[Title/Abstract])) OR (Neoplasm, Prostatic[Title/Abstract])) OR (Prostatic Neoplasm[Title/Abstract])) OR (Prostate Cancer[Title/Abstract])) OR (Cancer, Prostate[Title/Abstract])) OR (Cancers, Prostate[Title/Abstract])) OR (Prostate Cancers[Title/Abstract])) OR (Cancer of the Prostate[Title/Abstract])) OR (Prostatic Cancer[Title/Abstract])) OR (Cancer, Prostatic[Title/Abstract])) OR (Cancers, Prostatic[Title/Abstract])) OR (Prostatic Cancers[Title/Abstract])) OR (Cancer of Prostate[Title/Abstract]) |
| #3 | #1 OR #2 |
| #4 | Gastrointestinal Microbiome[MeSH Terms] |
| #5 | ((((((((((((((((((((((((((((((((((((Gastrointestinal Microbiome[Title/Abstract]) OR (Microbiome, Gastrointestinal[Title/Abstract])) OR (Gut Microbiome[Title/Abstract])) OR (Gut Microbiomes[Title/Abstract])) OR (Microbiome, Gut[Title/Abstract])) OR (Gut Microflora[Title/Abstract])) OR (Microflora, Gut[Title/Abstract])) OR (Gut Microbiota[Title/Abstract])) OR (Gut Microbiotas[Title/Abstract])) OR (Microbiota, Gut[Title/Abstract])) OR (Gastrointestinal Flora[Title/Abstract])) OR (Flora, Gastrointestinal[Title/Abstract])) OR (Gut Flora[Title/Abstract])) OR (Flora, Gut[Title/Abstract])) OR (Gastrointestinal Microbiota[Title/Abstract])) OR (Gastrointestinal Microbiotas[Title/Abstract])) OR (Microbiota, Gastrointestinal[Title/Abstract])) OR (Gastrointestinal Microbial Community[Title/Abstract])) OR (Gastrointestinal Microbial Communities[Title/Abstract])) OR (Microbial Community, Gastrointestinal[Title/Abstract])) OR (Gastrointestinal Microflora[Title/Abstract])) OR (Microflora, Gastrointestinal[Title/Abstract])) OR (Gastric Microbiome[Title/Abstract])) OR (Gastric Microbiomes[Title/Abstract])) OR (Microbiome, Gastric[Title/Abstract])) OR (Intestinal Microbiome[Title/Abstract])) OR (Intestinal Microbiomes[Title/Abstract])) OR (Microbiome, Intestinal[Title/Abstract])) OR (Intestinal Microbiota[Title/Abstract])) OR (Intestinal Microbiotas[Title/Abstract])) OR (Microbiota, Intestinal[Title/Abstract])) OR (Intestinal Microflora[Title/Abstract])) OR (Microflora, Intestinal[Title/Abstract])) OR (Intestinal Flora[Title/Abstract])) OR (Flora, Intestinal[Title/Abstract])) OR (Enteric Bacteria[Title/Abstract])) OR (Bacteria, Enteric[Title/Abstract]) |
| #6 | #4 OR #5 |
| #7 | #3 AND #6 |
