# Supplementary material for: Gut microbiota in patients with prostate cancer: a systematic review and meta-analysis
Source: BMC Cancer. 2024 Feb 24;24:261. doi: 10.1186/s12885-024-12018-x (PMC10893726; doi:10.1186/s12885-024-12018-x)

**Figure S1.** Sensitivity analysis of Chao1 in subgroup of prostate cancer patient vs control.


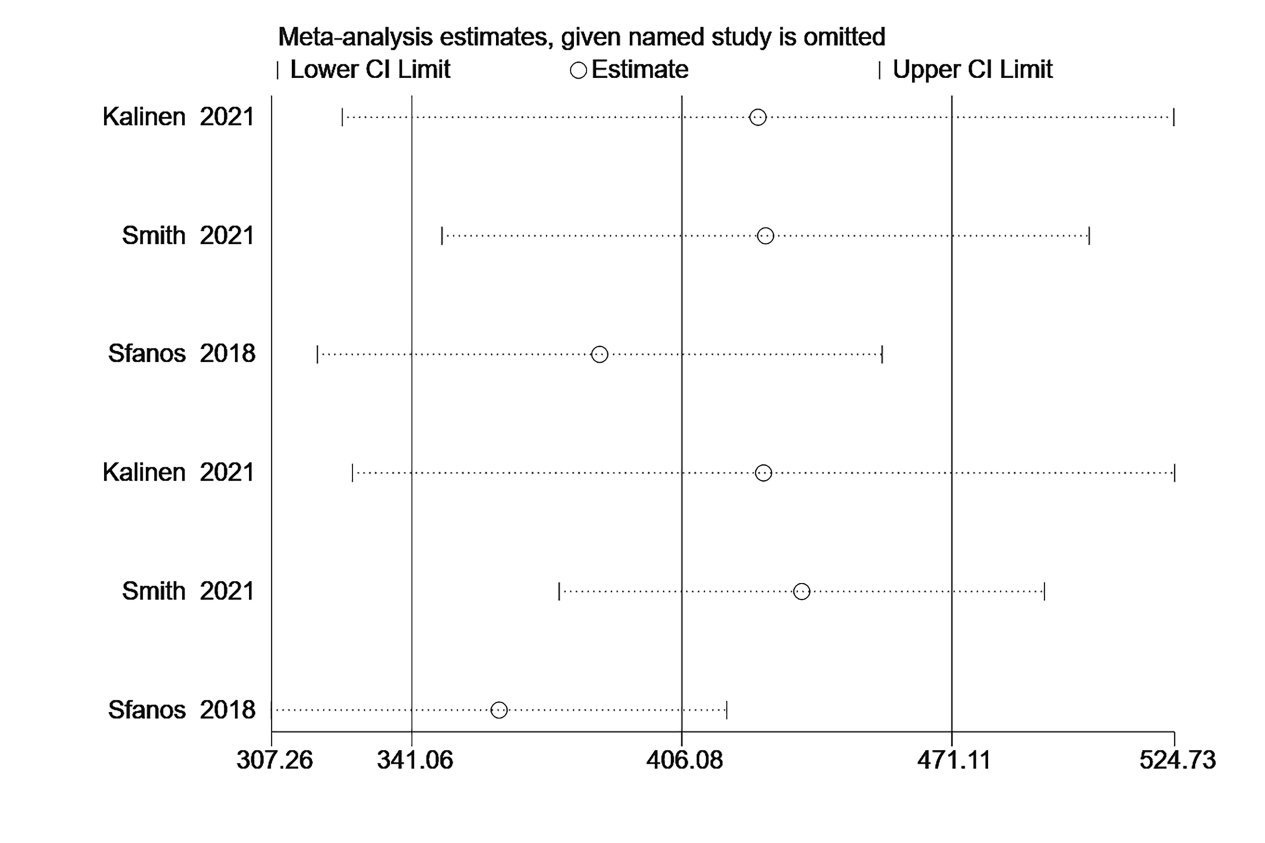


**Figure S2.** Sensitivity analysis of Observed Species in subgroup of prostate cancer patient vs control.


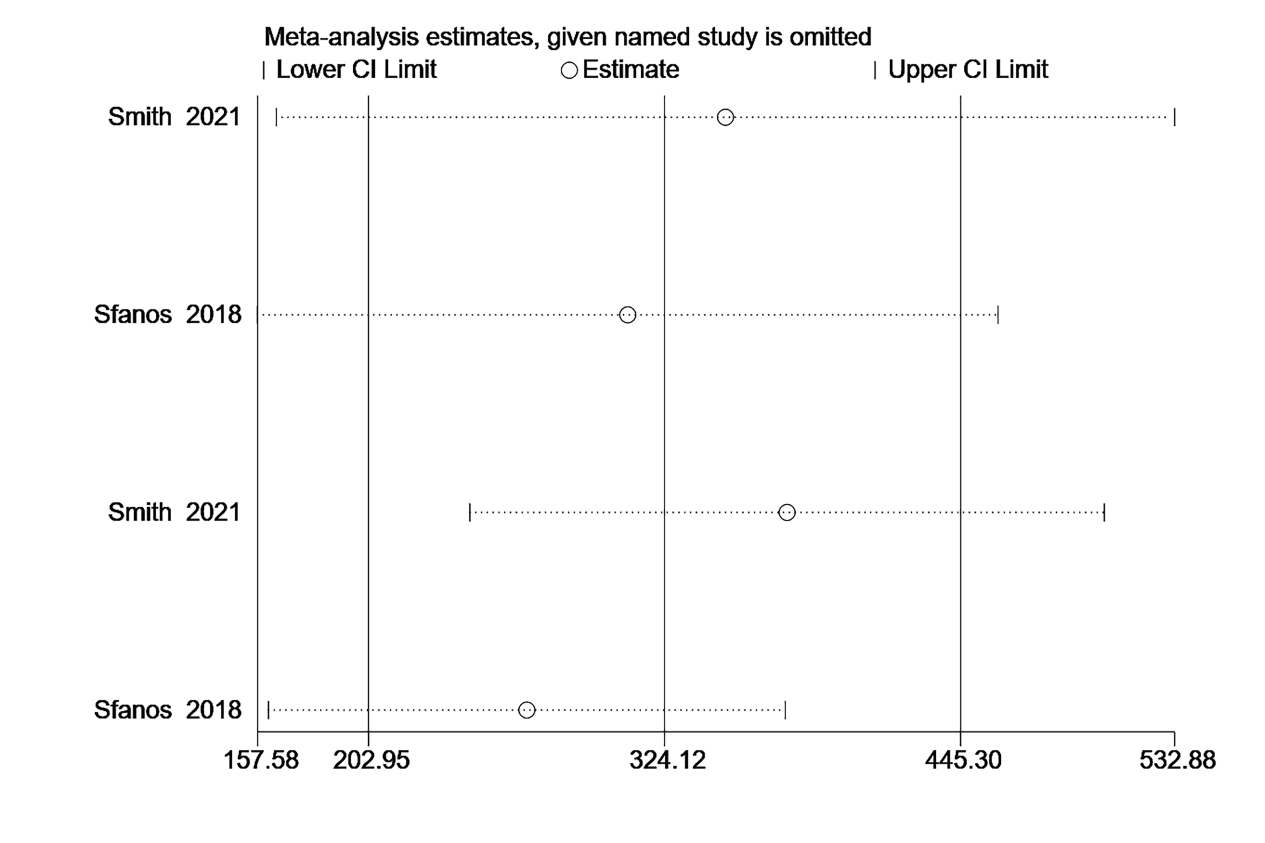


**Figure S3.** Sensitivity analysis of Shannon in subgroup of prostate cancer patient vs control.


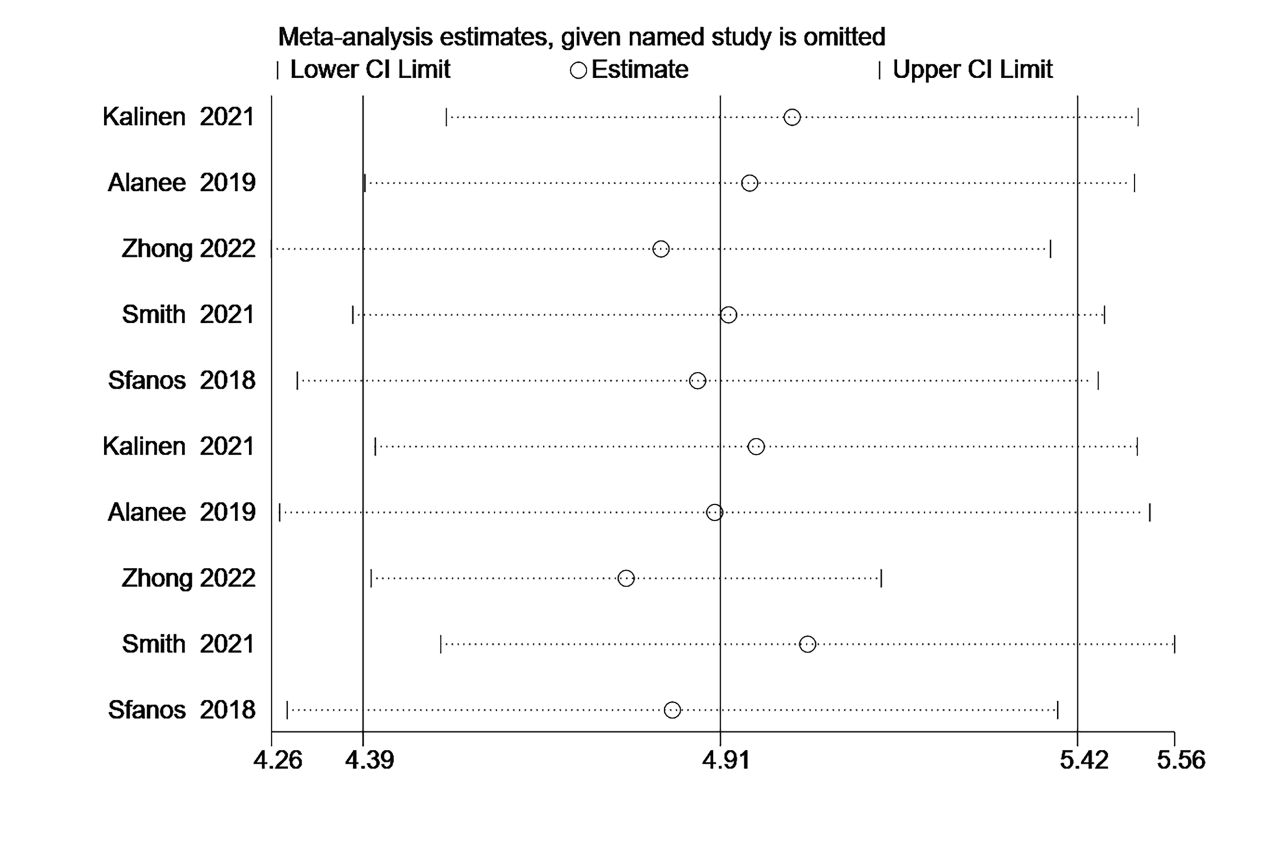


**Figure S4.** Sensitivity analysis of Simpson in subgroup of prostate cancer patient vs control.


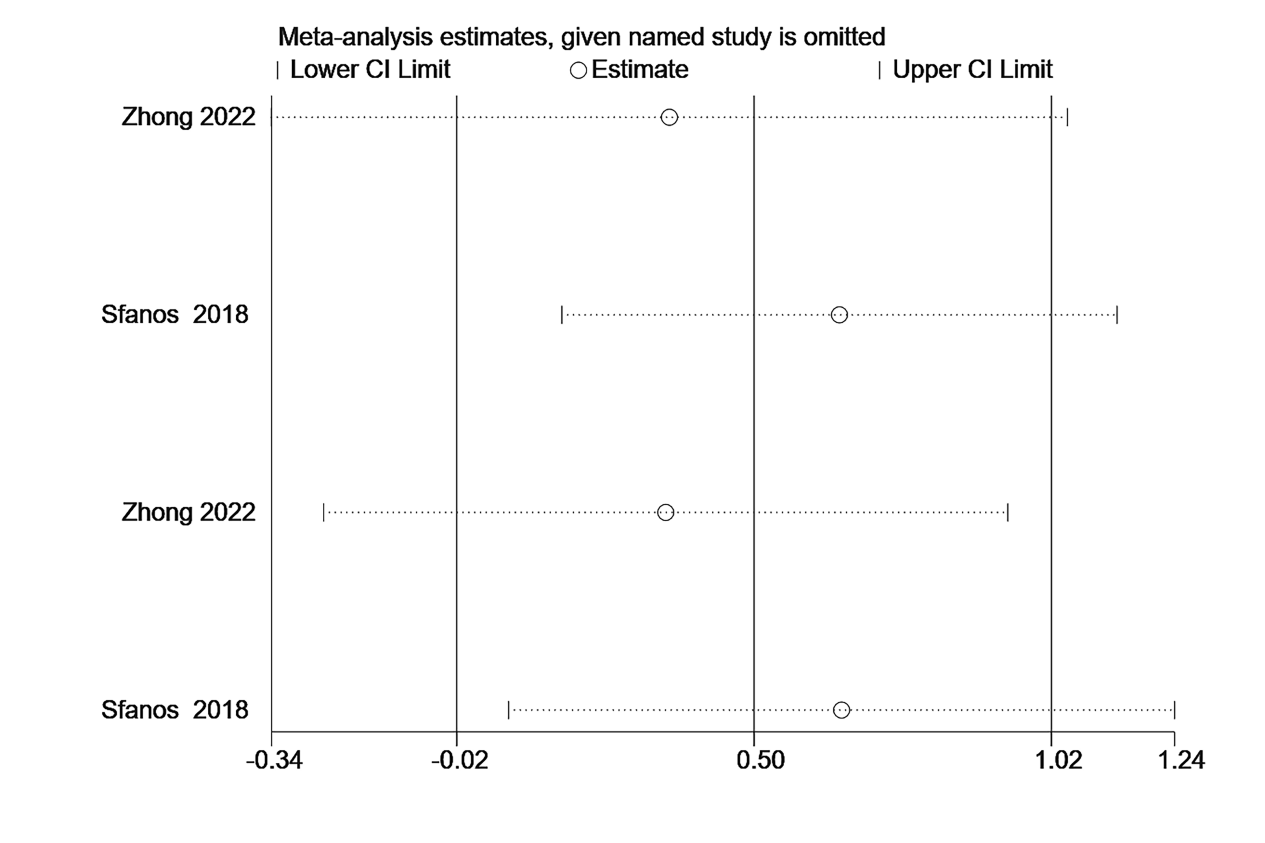

Supplement: Supplementary file 2 — Supplementary Material 2. [file 12885_2024_12018_MOESM2_ESM.zip › Additional file 2/Figure S1-4. Sensitivity analysis of alpha-diversity.docx]
