# Supplementary material for: Gut microbiota in patients with prostate cancer: a systematic review and meta-analysis
Source: BMC Cancer. 2024 Feb 24;24:261. doi: 10.1186/s12885-024-12018-x (PMC10893726; doi:10.1186/s12885-024-12018-x)

**Figure S16.** Forest plot of relative abundance of *Actinobacteria* in prostate patients and controls.


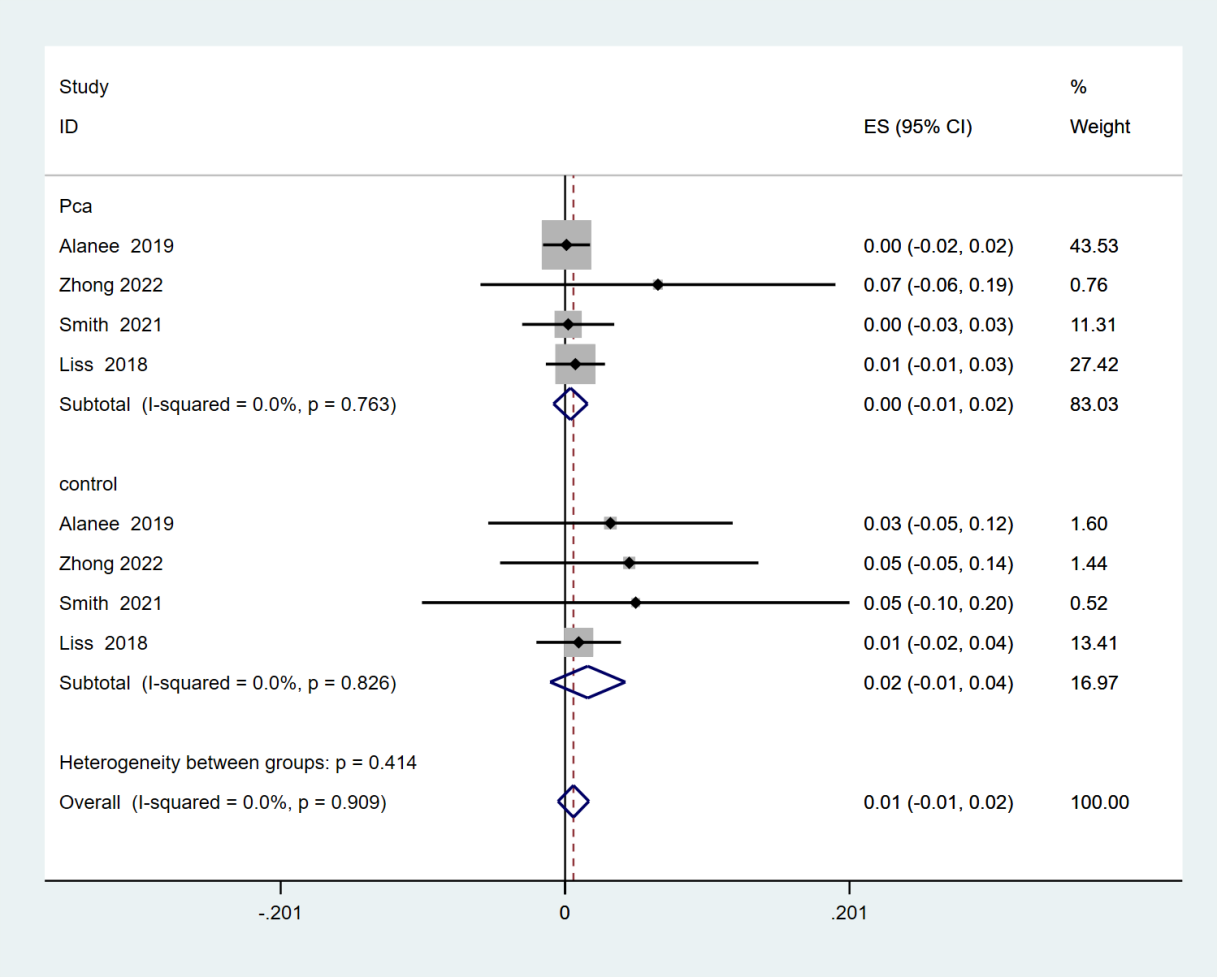


**Figure S17.** Forest plot of relative abundance of *Bacteroidia* in prostate patients and controls.


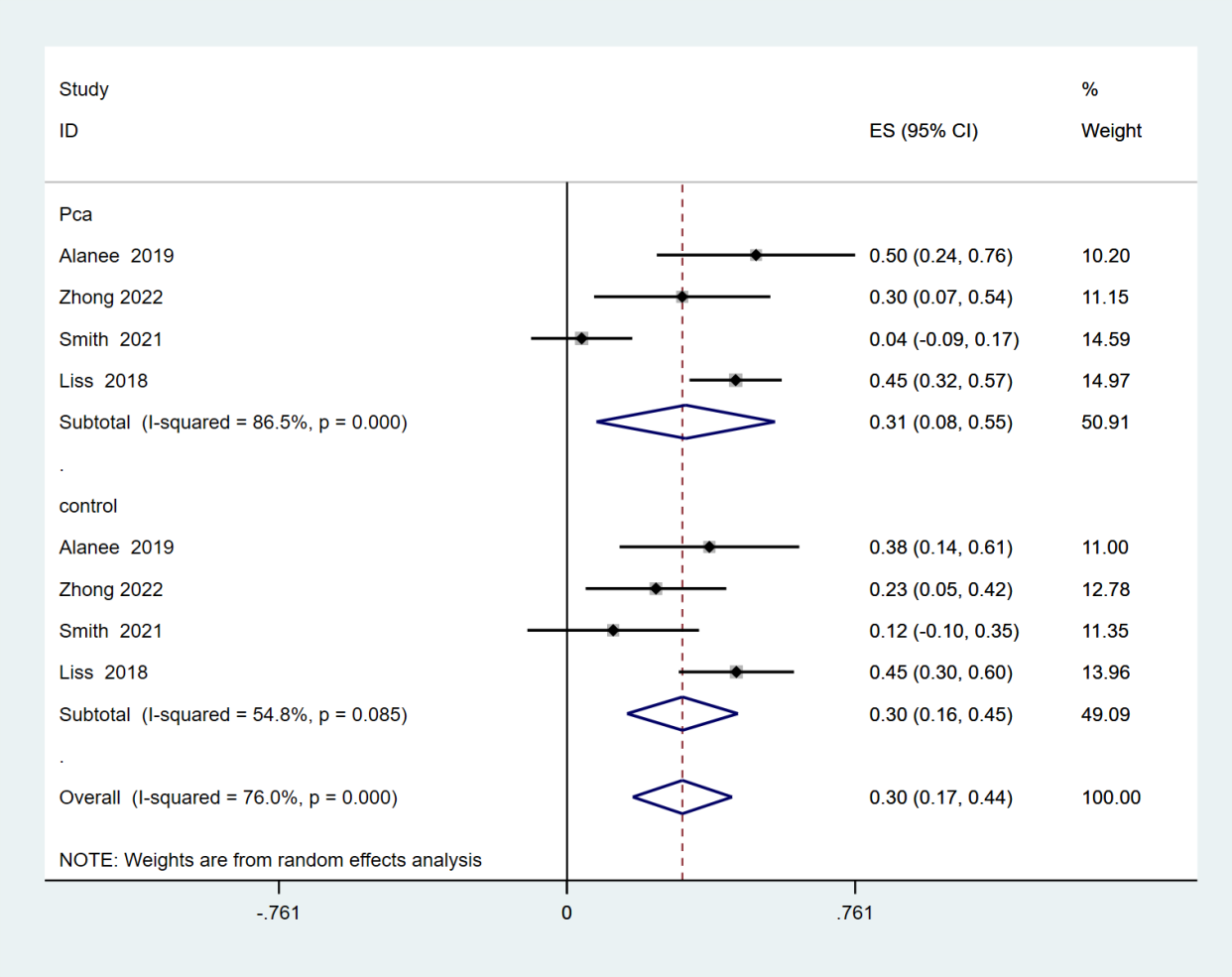


**Figure S18.** Forest plot of relative abundance of *Bacilli* in prostate patients and controls.


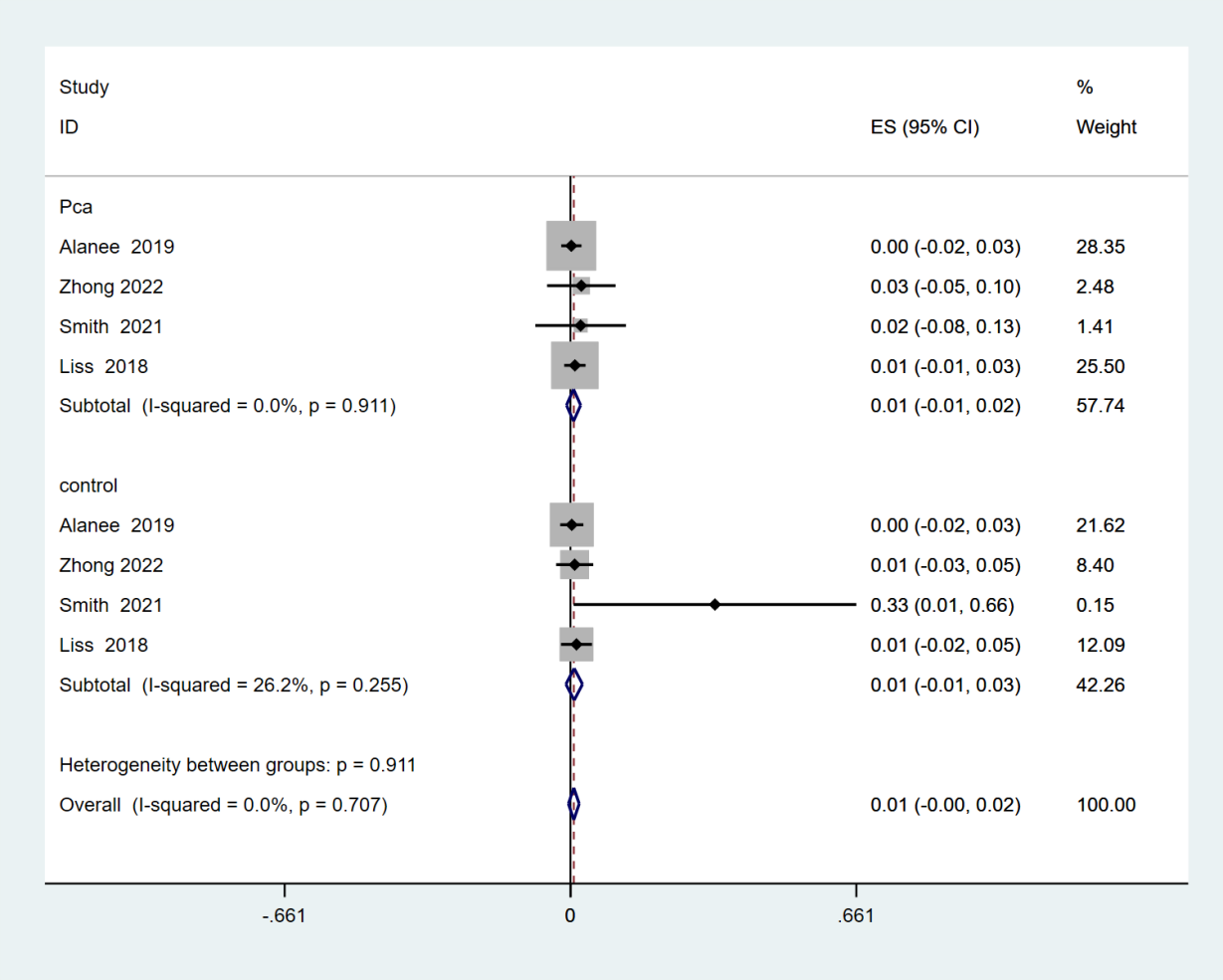


**Figure S19.** Forest plot of relative abundance of *Clostridia* in prostate patients and controls.


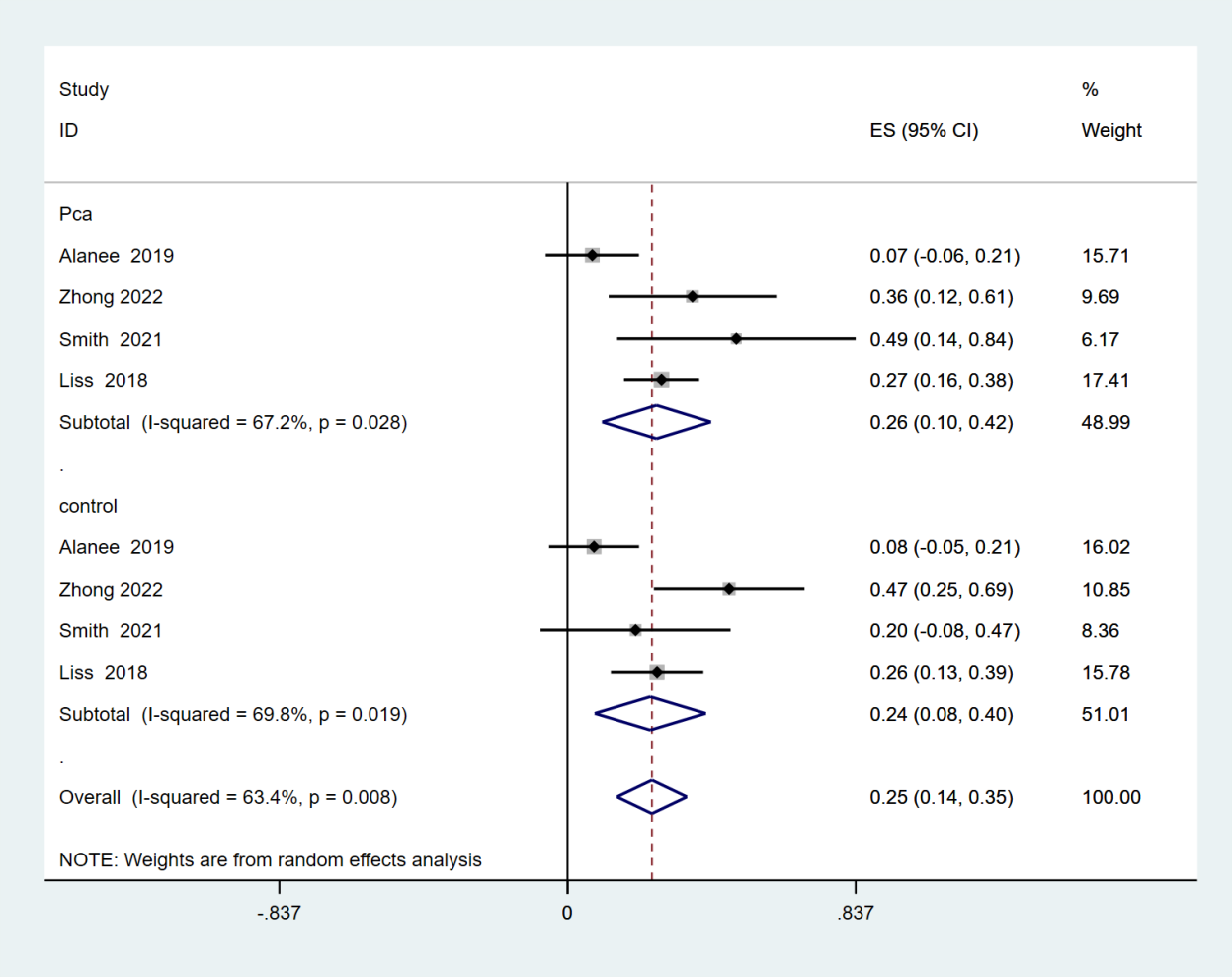


**Figure S20.** Forest plot of relative abundance of *Erysipelotrichia* in prostate patients and controls.


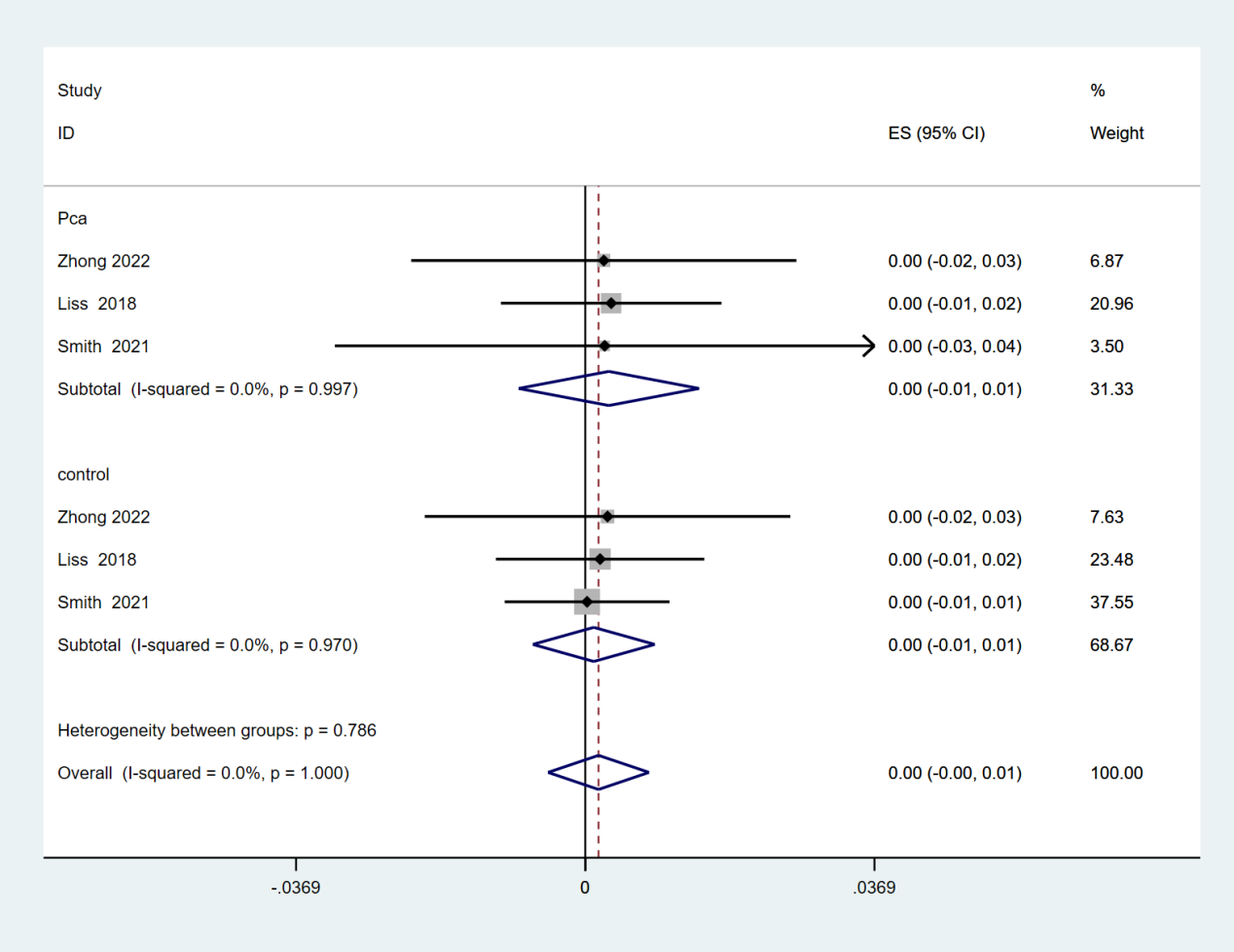


**Figure S21.** Forest plot of relative abundance of *Negativicutes* in prostate patients and controls.


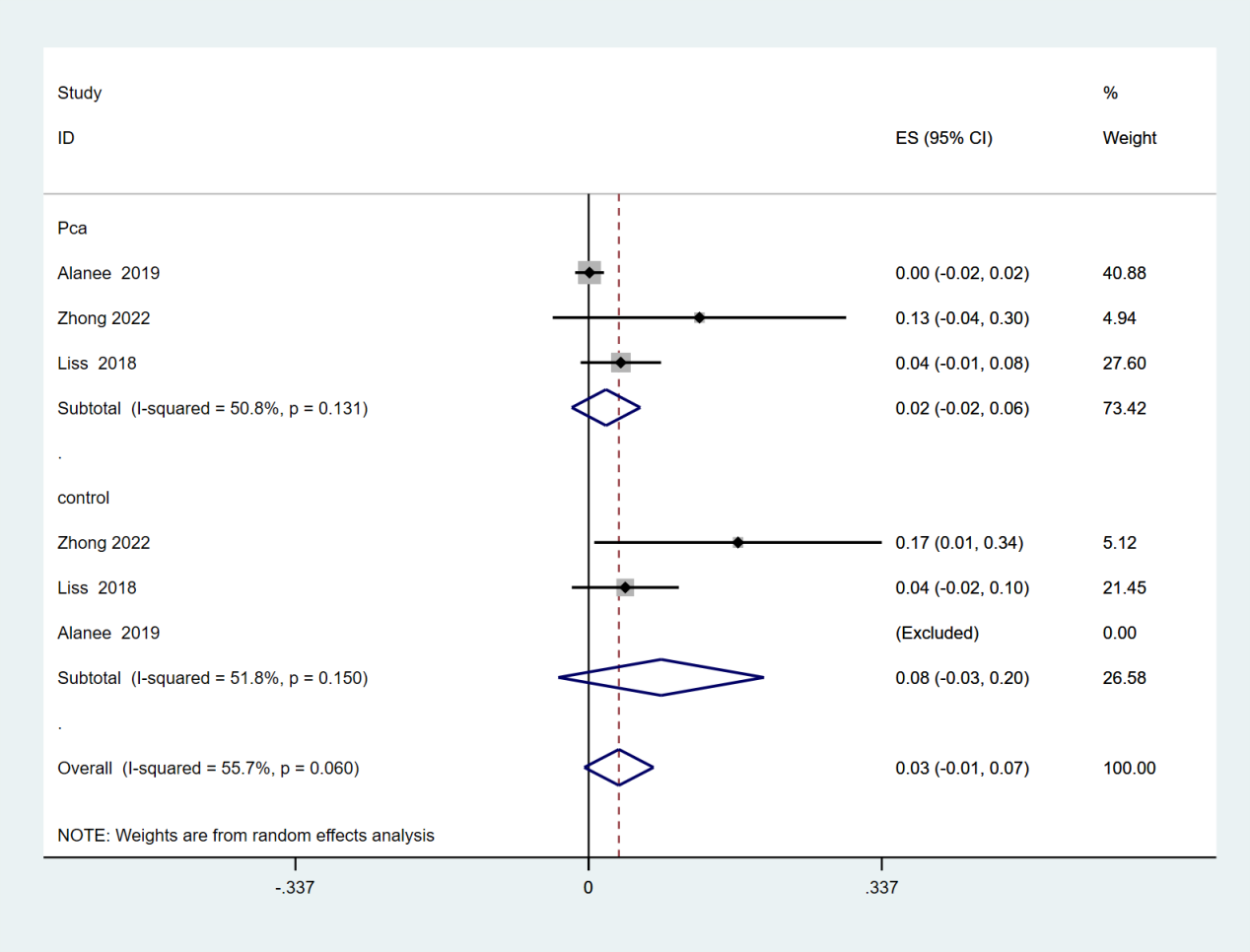


**Figure S22.** Forest plot of relative abundance of *Gammaproteobacteria* in prostate patients and controls.


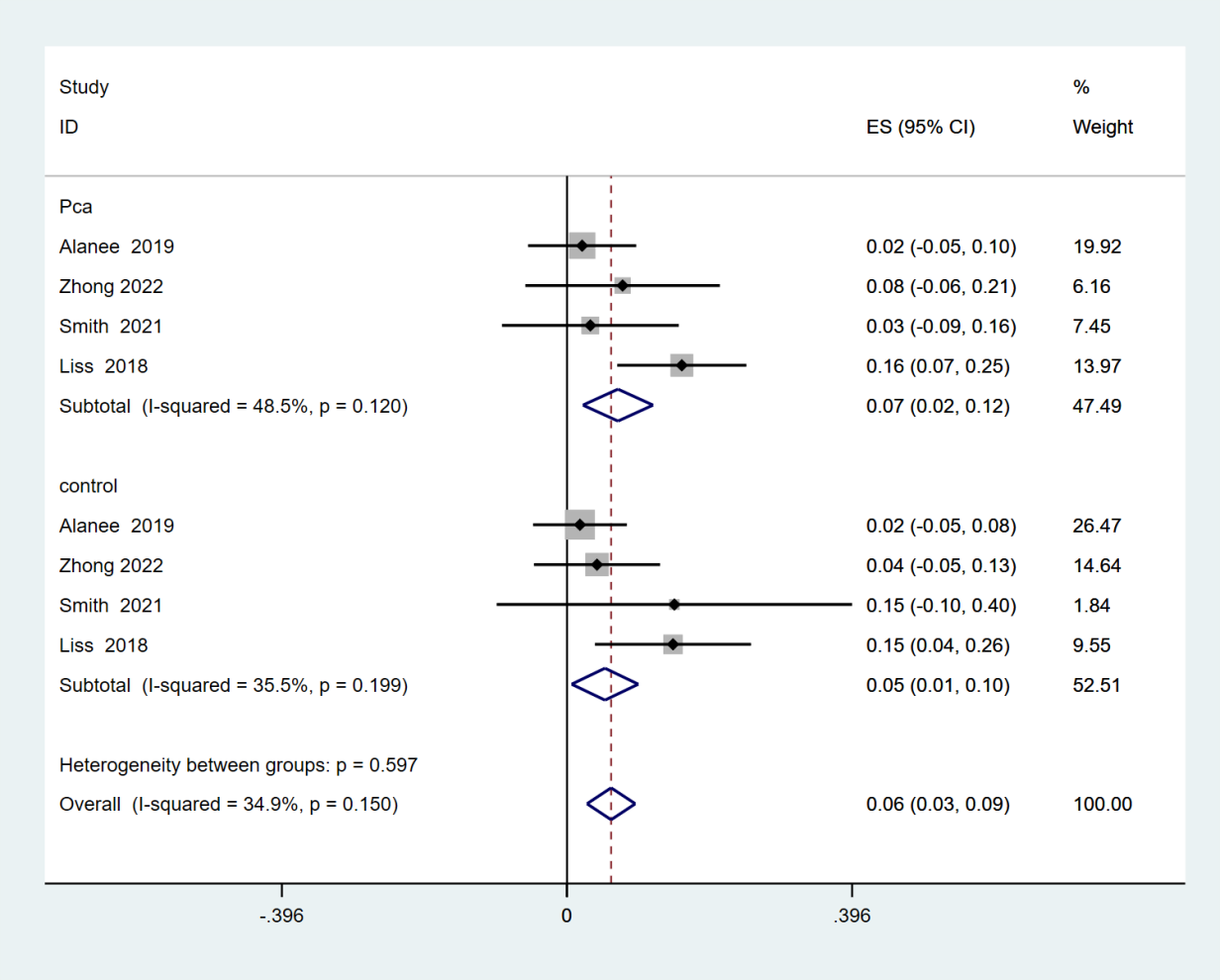


**Figure S23.** Forest plot of relative abundance of *Coriobacteriia* in prostate patients and controls.


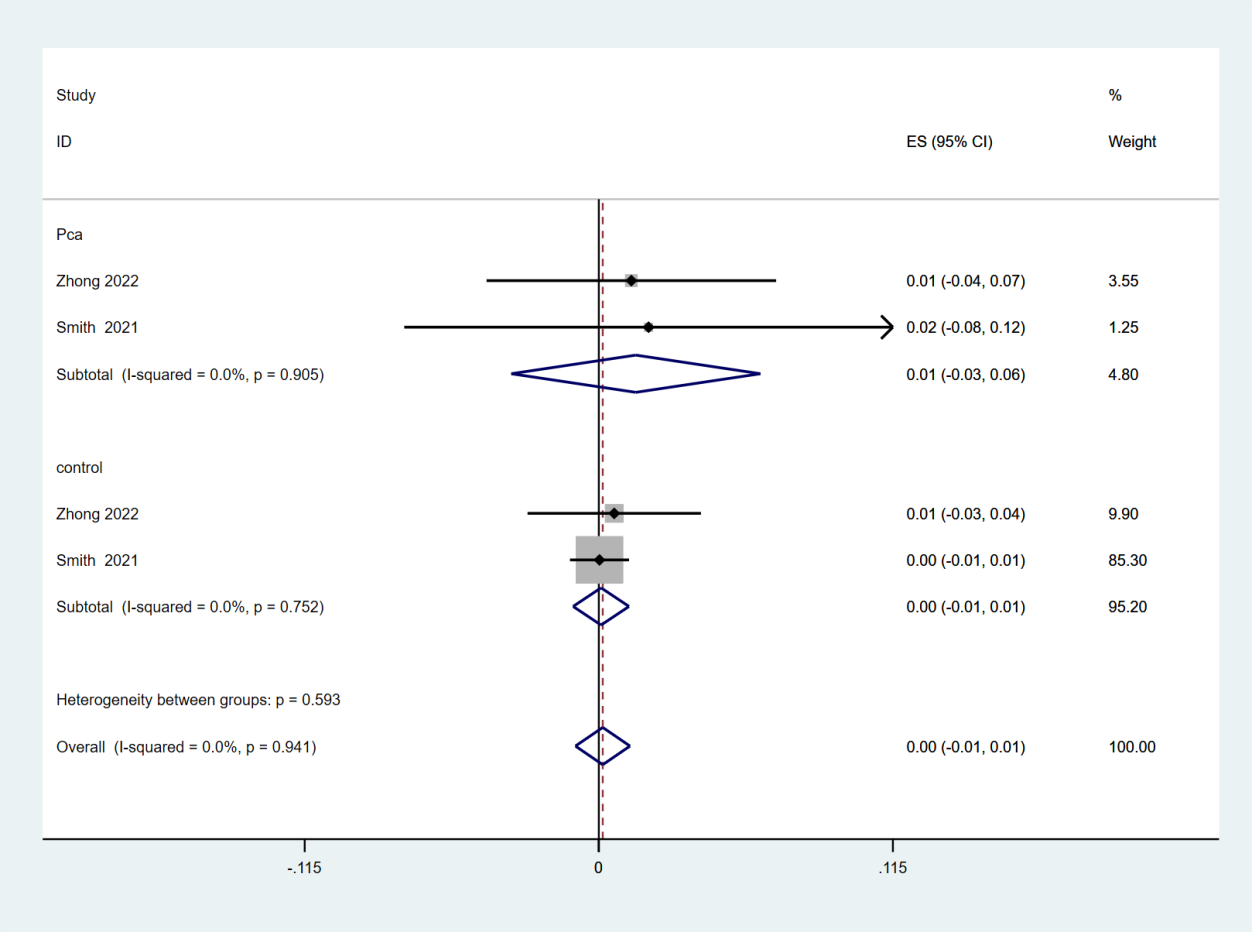


**Figure S24.** Forest plot of relative abundance of *Deltaproteobacteria* in prostate patients and controls.


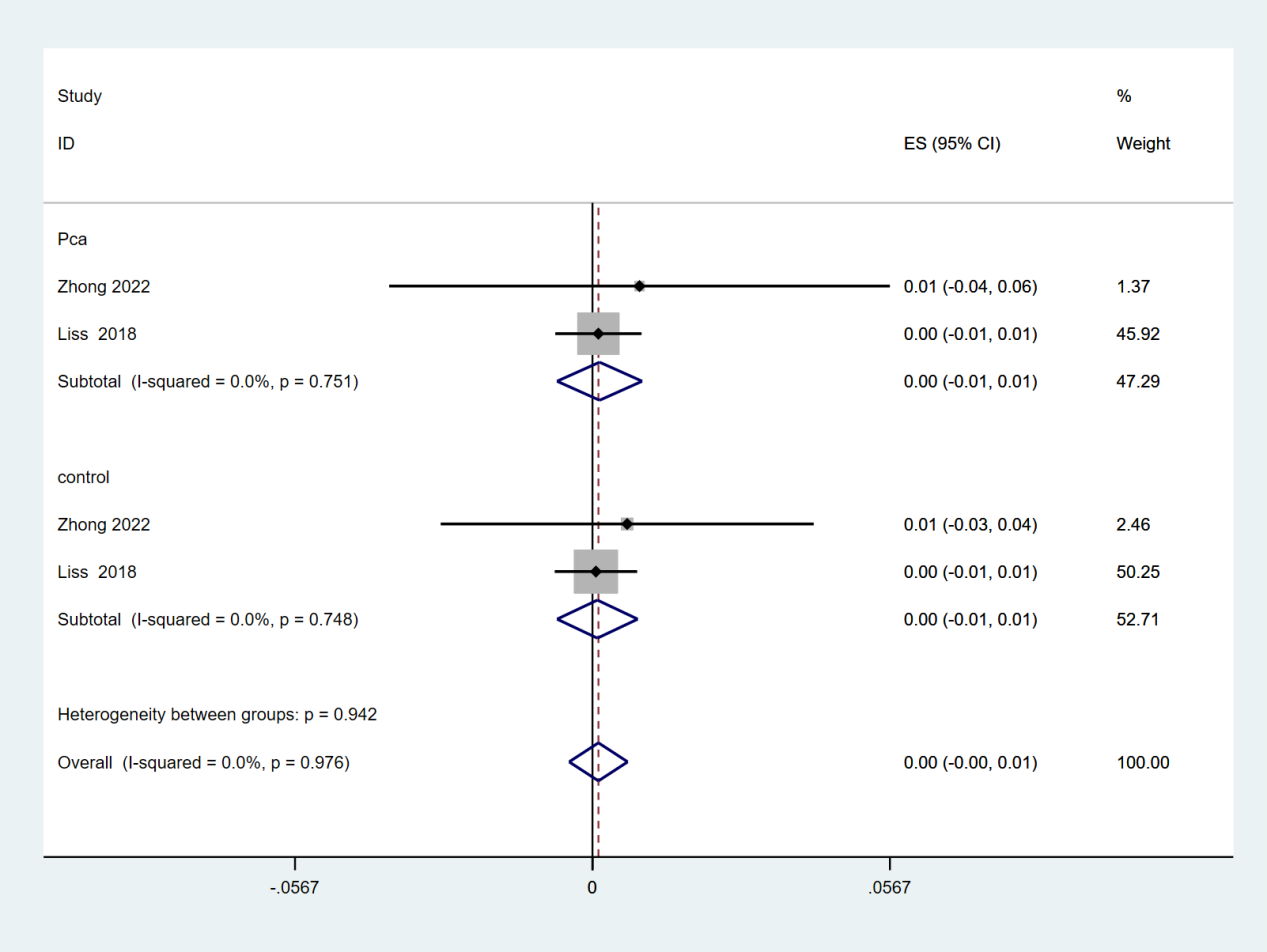


**Figure S25.** Forest plot of relative abundance of *Verrucomicrobiae* in prostate patients and controls.


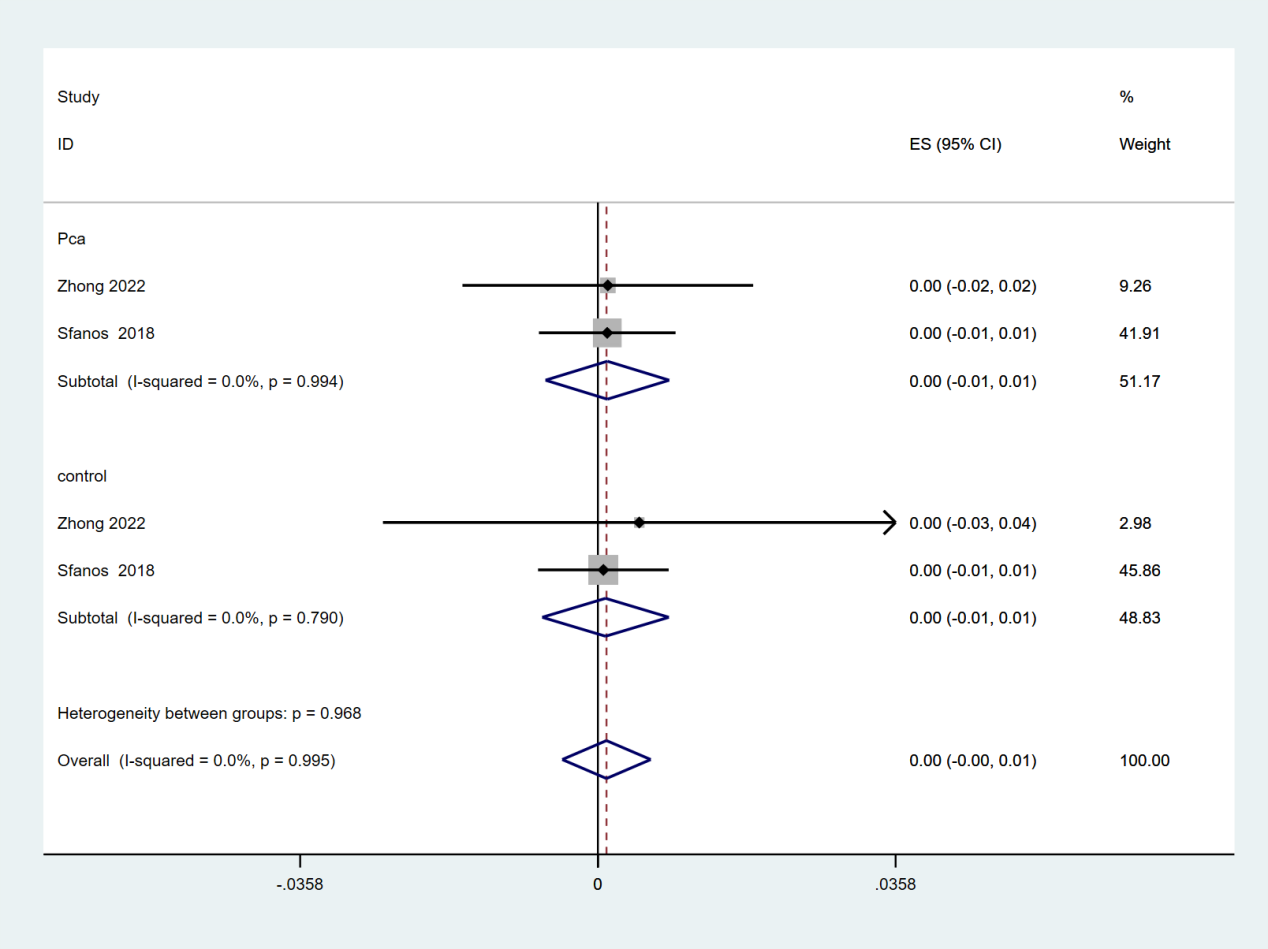


**Figure S26.** Forest plot of relative abundance of *Fusobacteria* in prostate patients and controls.


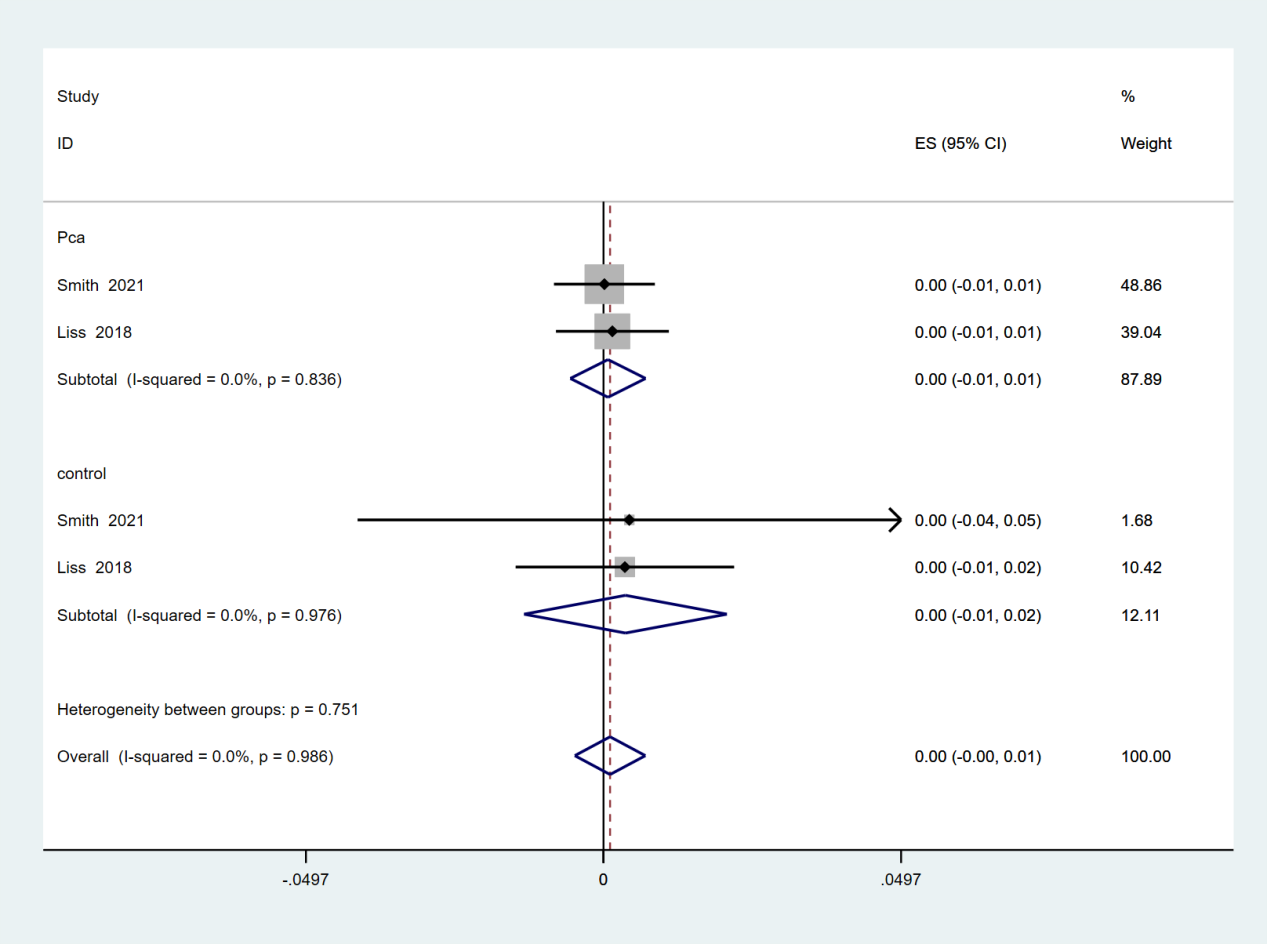


**Figure S27.** Forest plot of relative abundance of *Alphaproteobacteria* in prostate patients and controls.


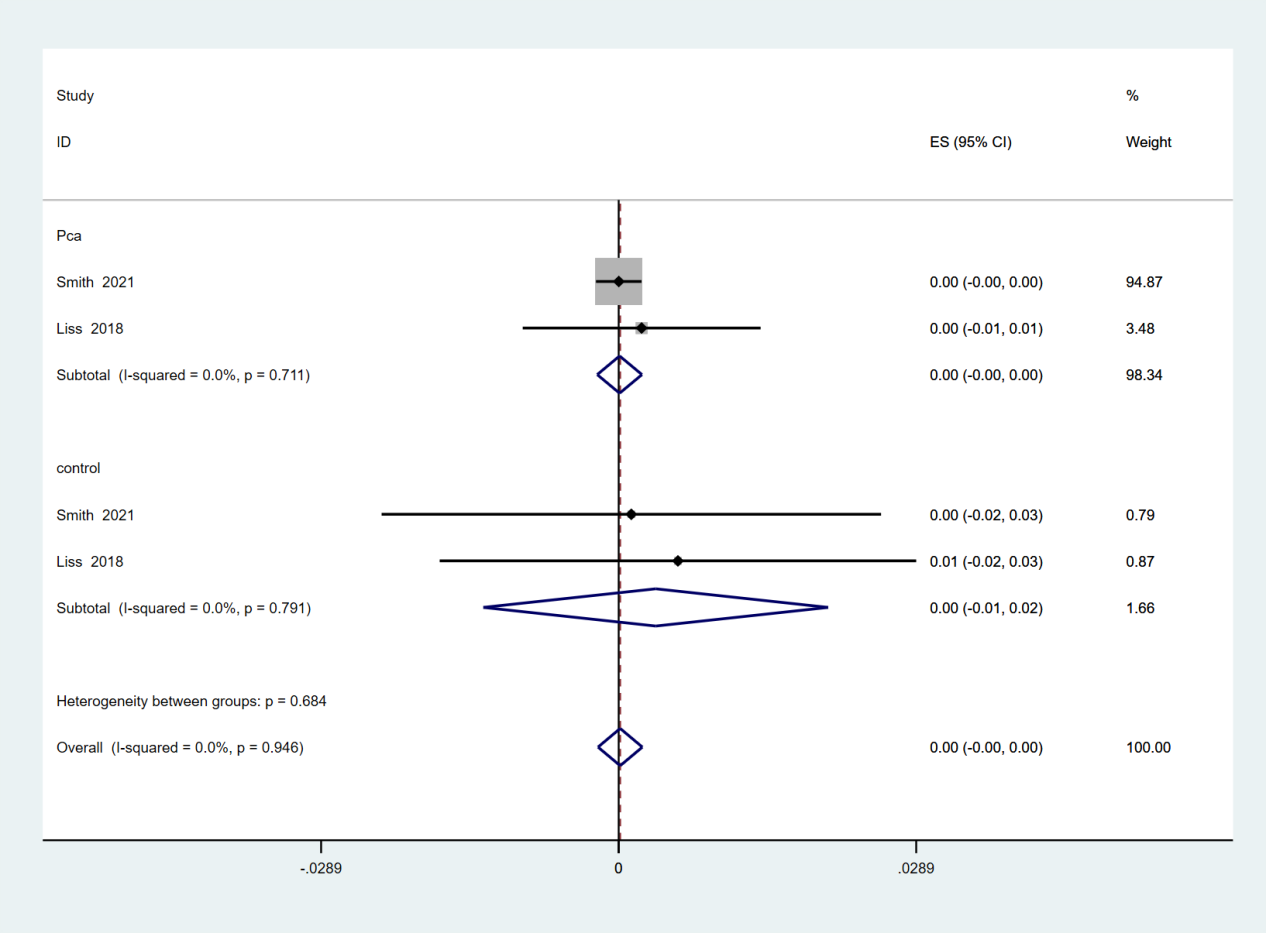


**Figure S28.** Forest plot of relative abundance of *Betaproteobacteria* in prostate patients and controls.


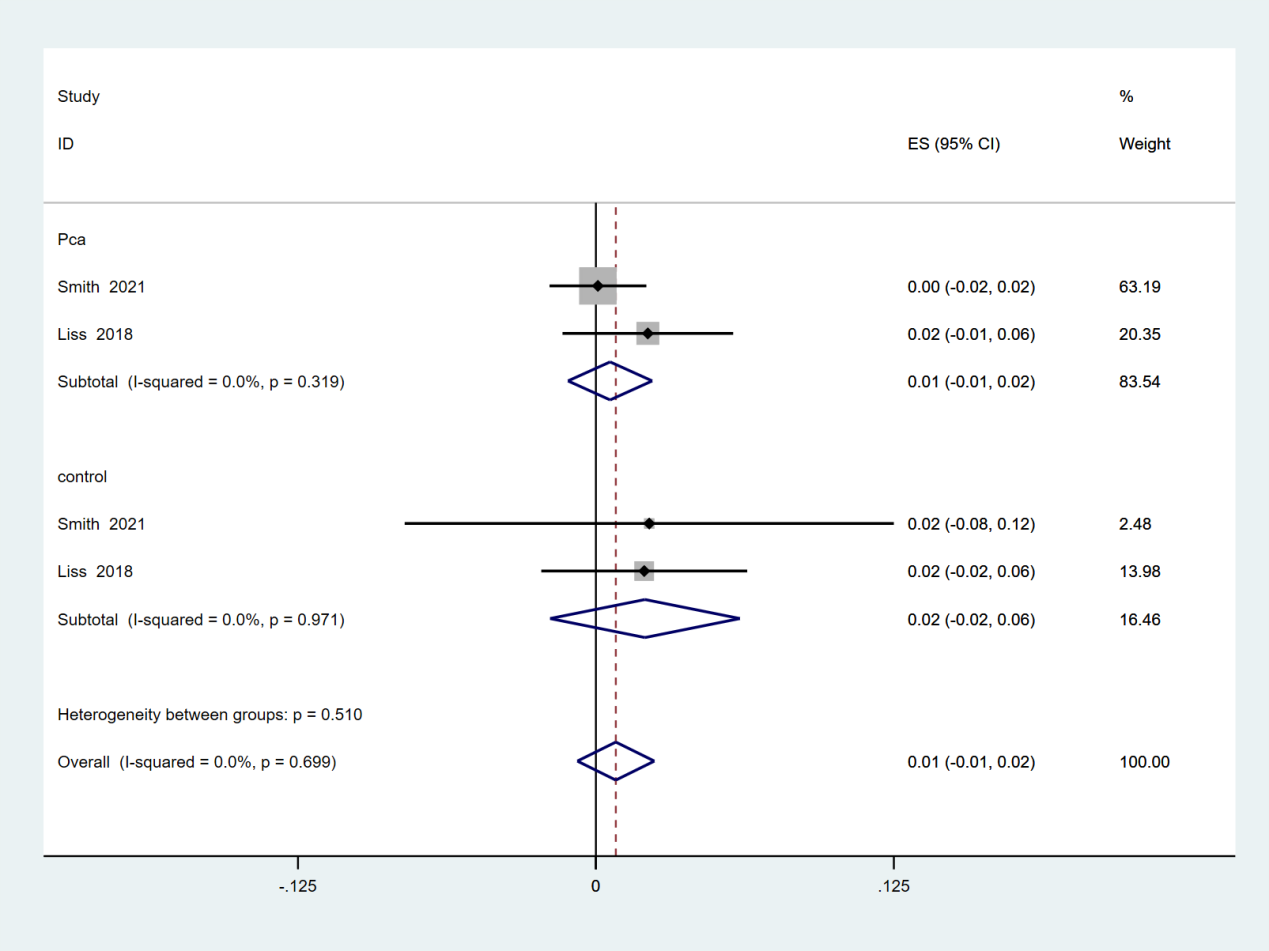


**Figure S29.** Forest plot of relative abundance of *Epsilonproteobacteria* in prostate patients and controls.


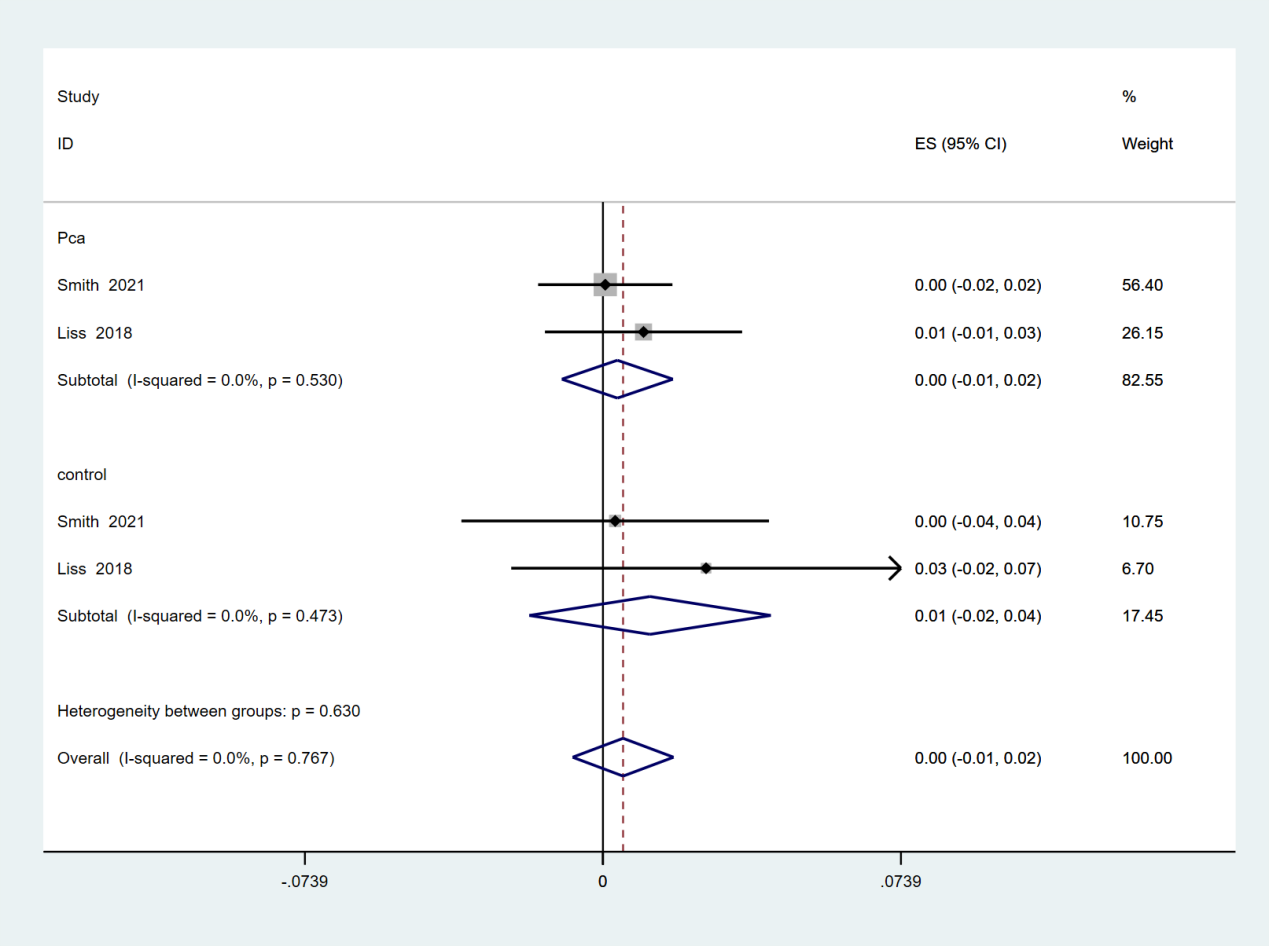


**Figure S30.** Forest plot of relative abundance of *Synergistia* in prostate patients and controls.


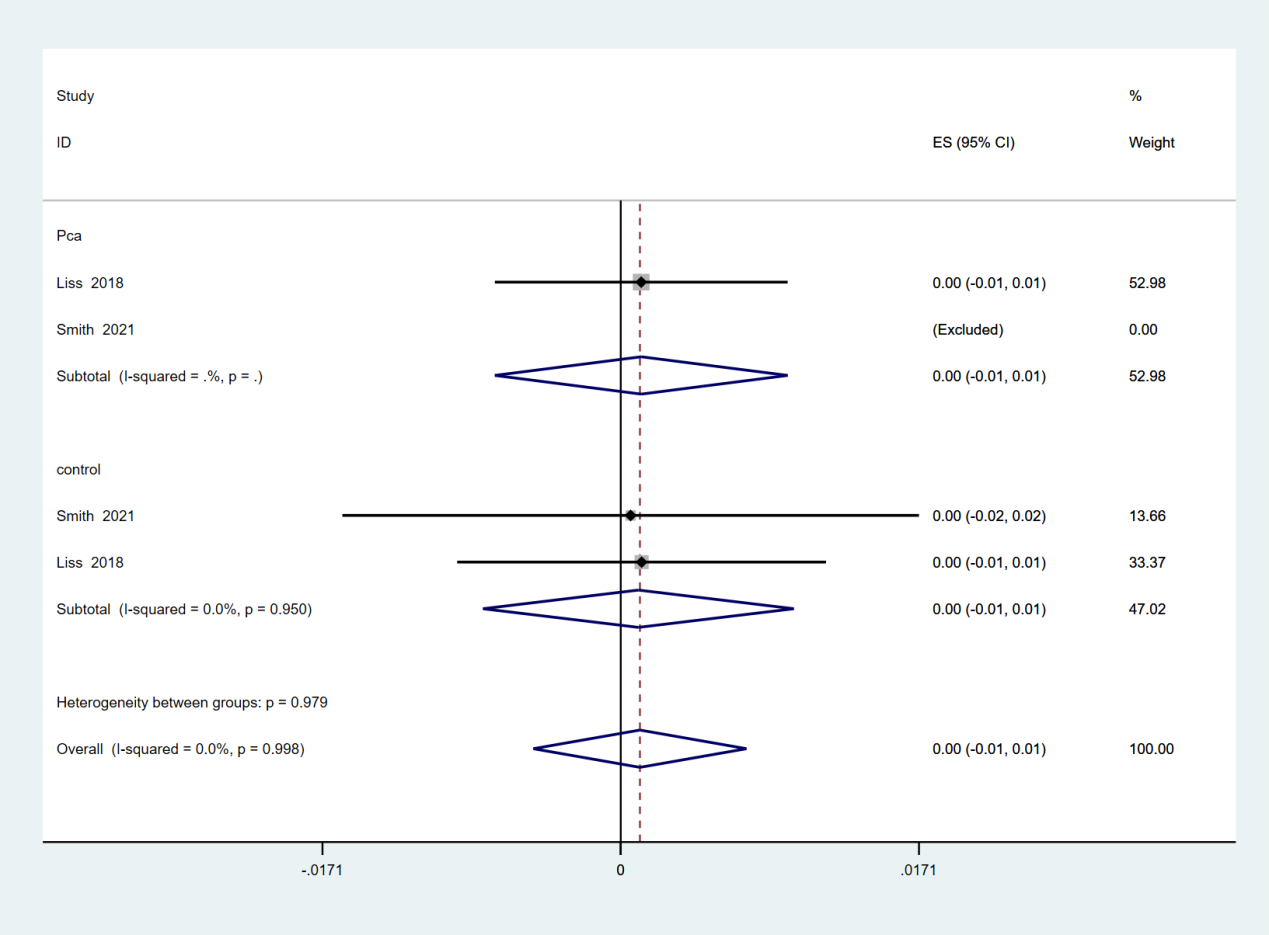


**Figure S31.** Forest plot of relative abundance of *Spirochaetes* in prostate patients and controls.


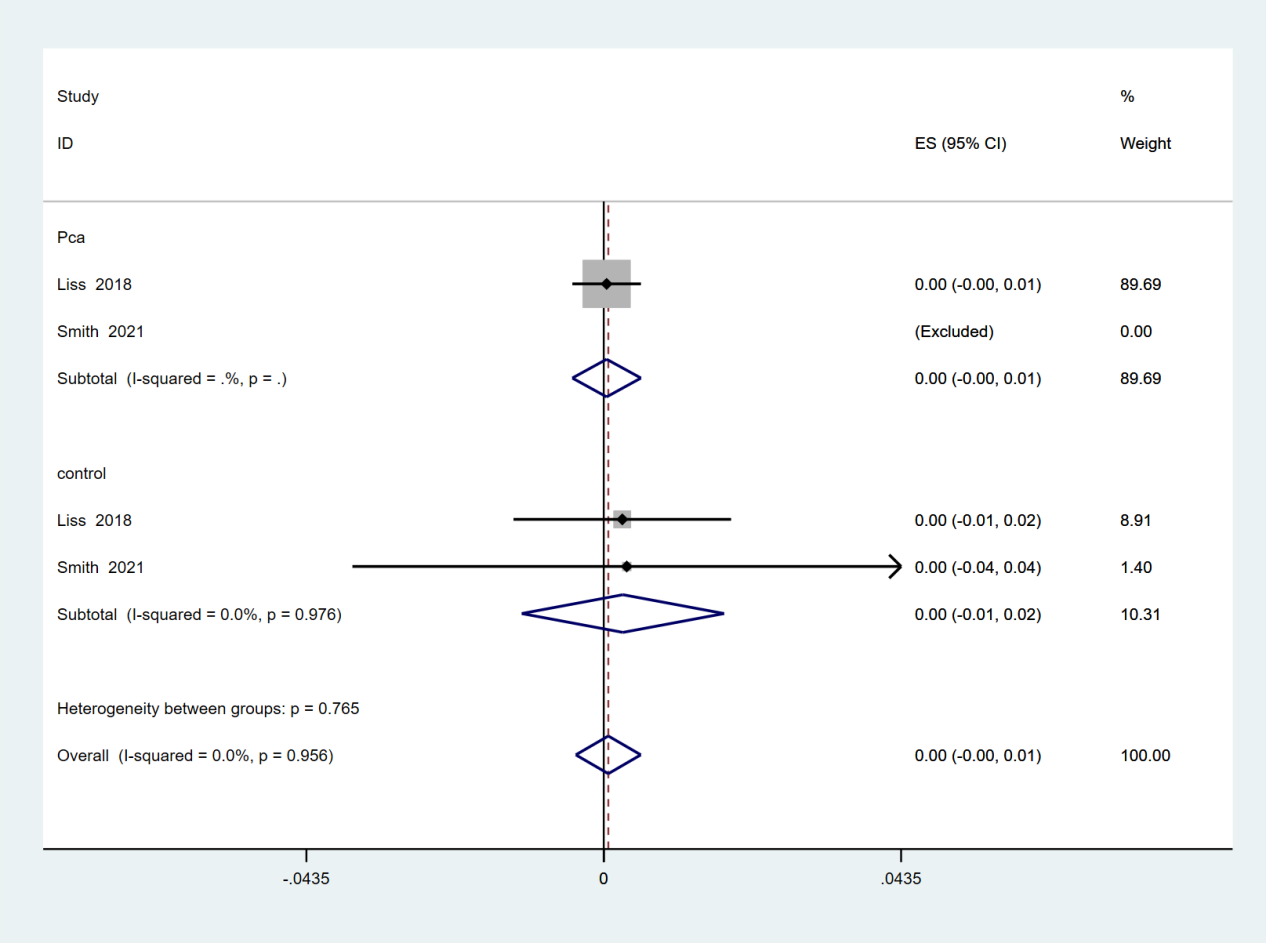

Supplement: Supplementary file 3 — Supplementary Material 3. [file 12885_2024_12018_MOESM3_ESM.zip › Additional file 3/Figure S16-31. Forest plot of relative abundance of GM in at class level.docx]
