# Supplementary material for: Gut microbiota in patients with prostate cancer: a systematic review and meta-analysis
Source: BMC Cancer. 2024 Feb 24;24:261. doi: 10.1186/s12885-024-12018-x (PMC10893726; doi:10.1186/s12885-024-12018-x)

**Supplementary Figure 32.** Sensitivity analysis of relative abundance of *Bacteroidia* in subgroup of prostat cancer patient vs control.


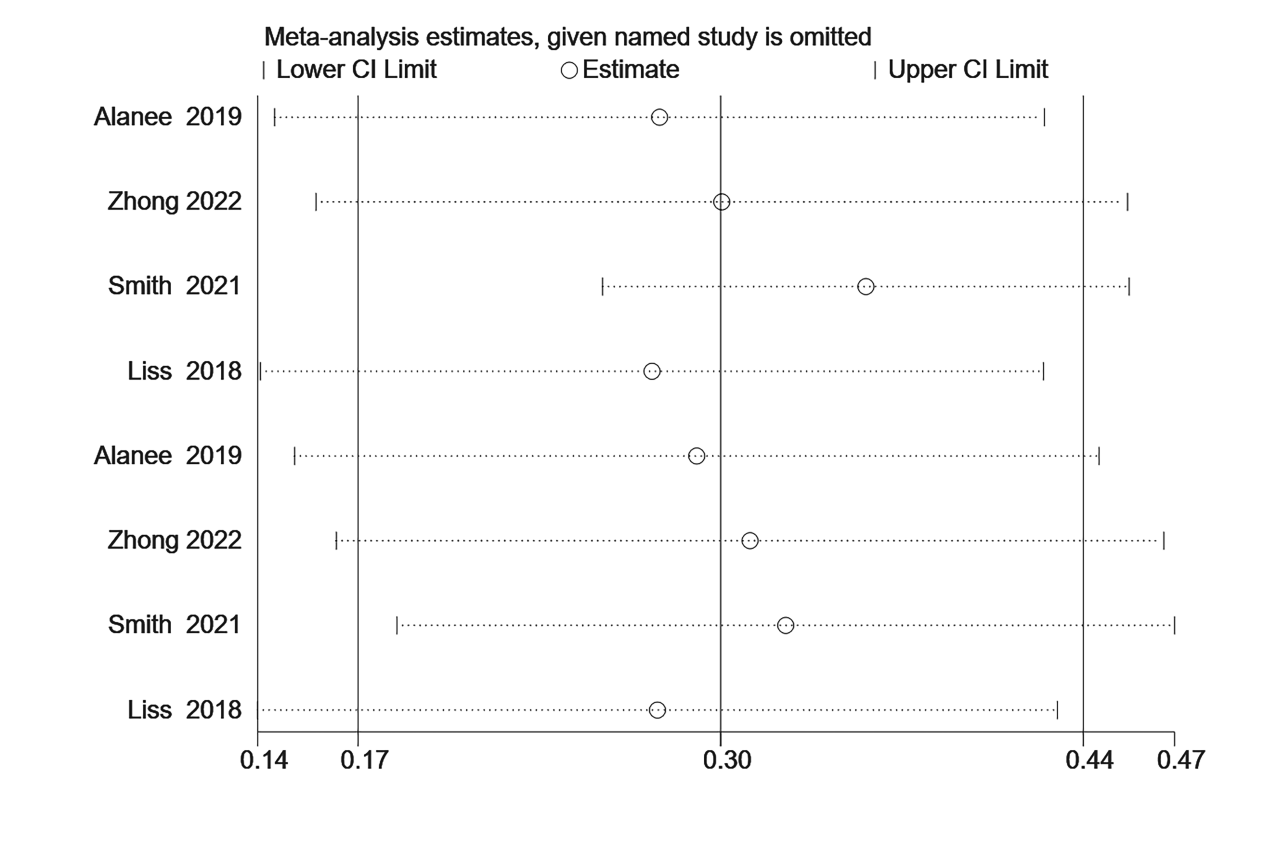


**Supplementary Figure 33.** Sensitivity analysis of relative abundance of *Clostridia* in subgroup of prostat cancer patient vs control.


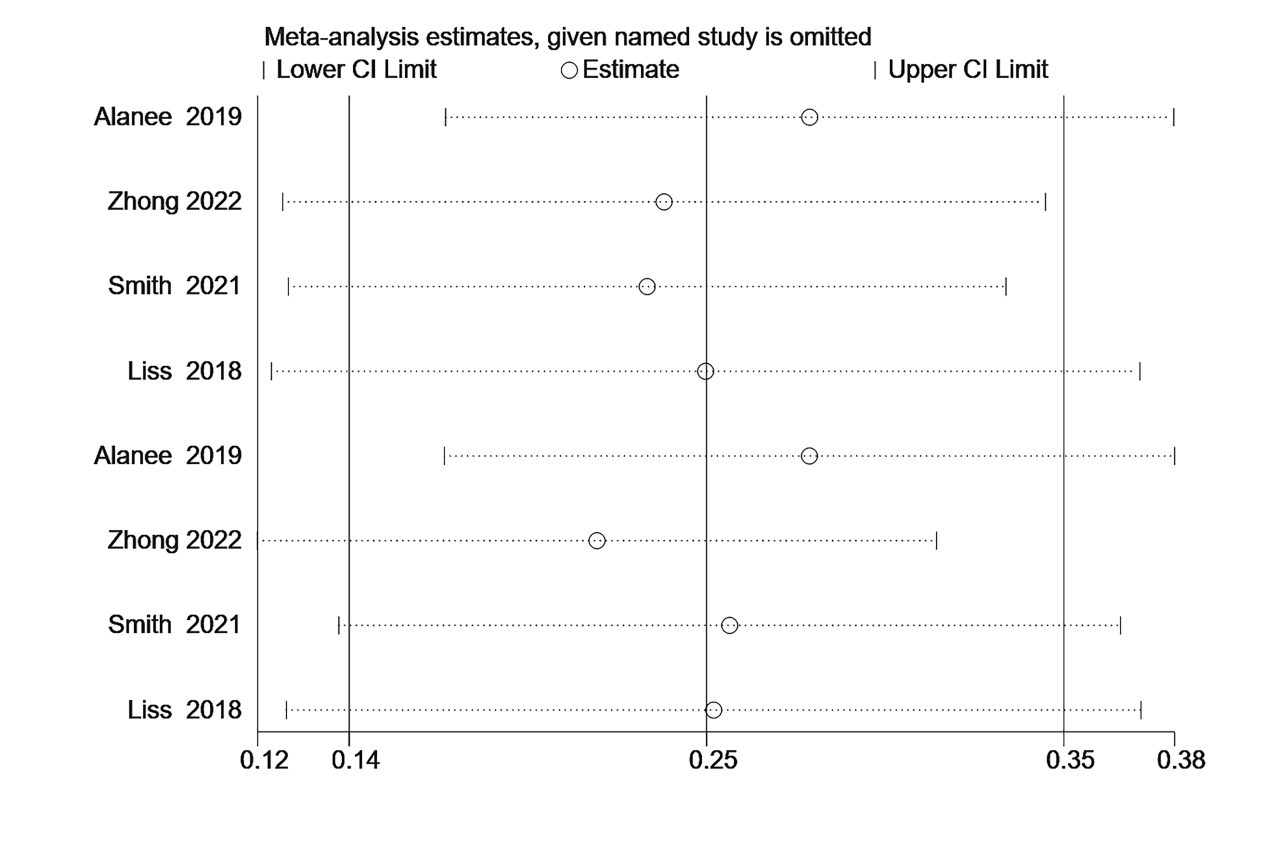


**Supplementary Figure 34.** Sensitivity analysis of relative abundance of *Negativicutes* in subgroup of prostat cancer patient vs control.


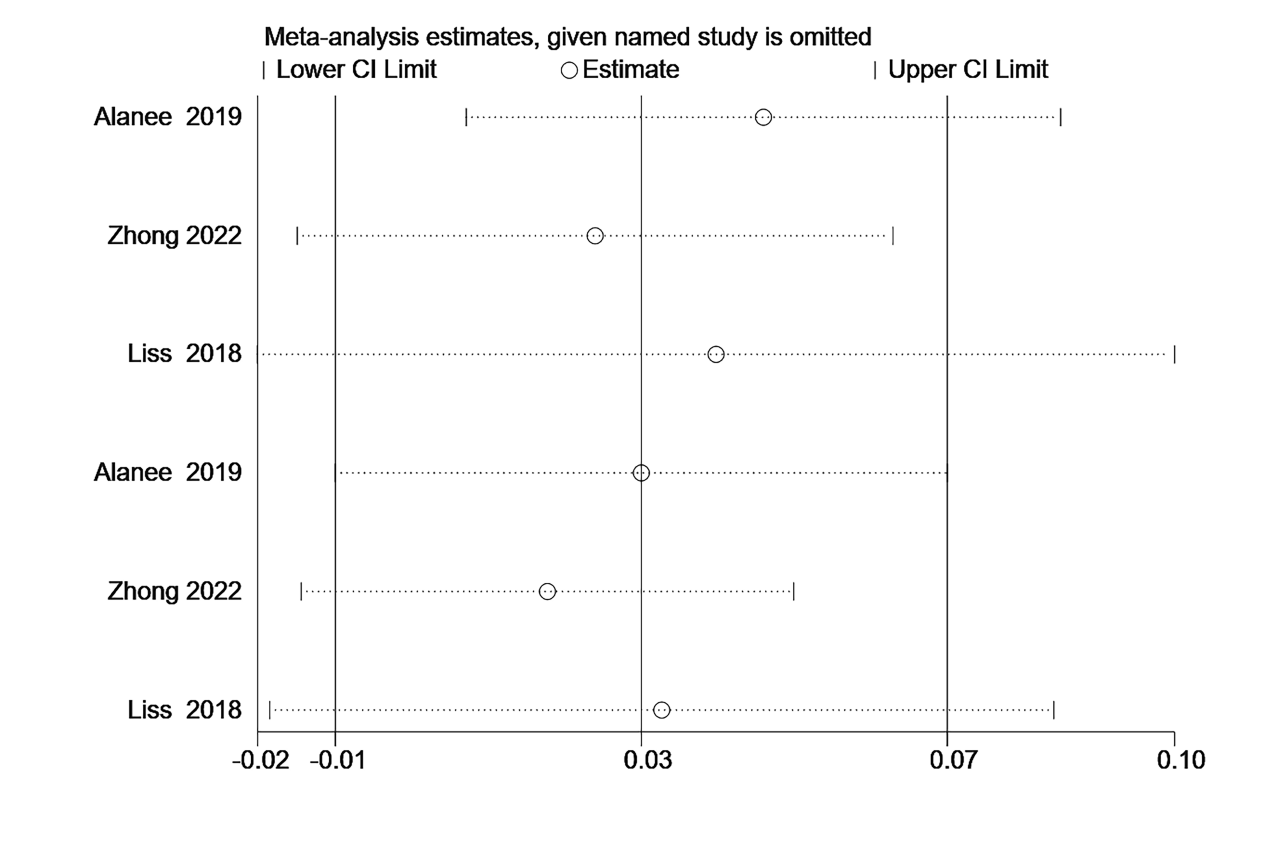

Supplement: Supplementary file 3 — Supplementary Material 3. [file 12885_2024_12018_MOESM3_ESM.zip › Additional file 3/Figure S32-34. Sensitivity analysis of relative abundance of GM in at class level.docx]
