# Supplementary material for: Gut microbiota in patients with prostate cancer: a systematic review and meta-analysis
Source: BMC Cancer. 2024 Feb 24;24:261. doi: 10.1186/s12885-024-12018-x (PMC10893726; doi:10.1186/s12885-024-12018-x)

**Figure S35.** Forest plot of relative abundance of *Bacteroidales* in prostate patients and controls.


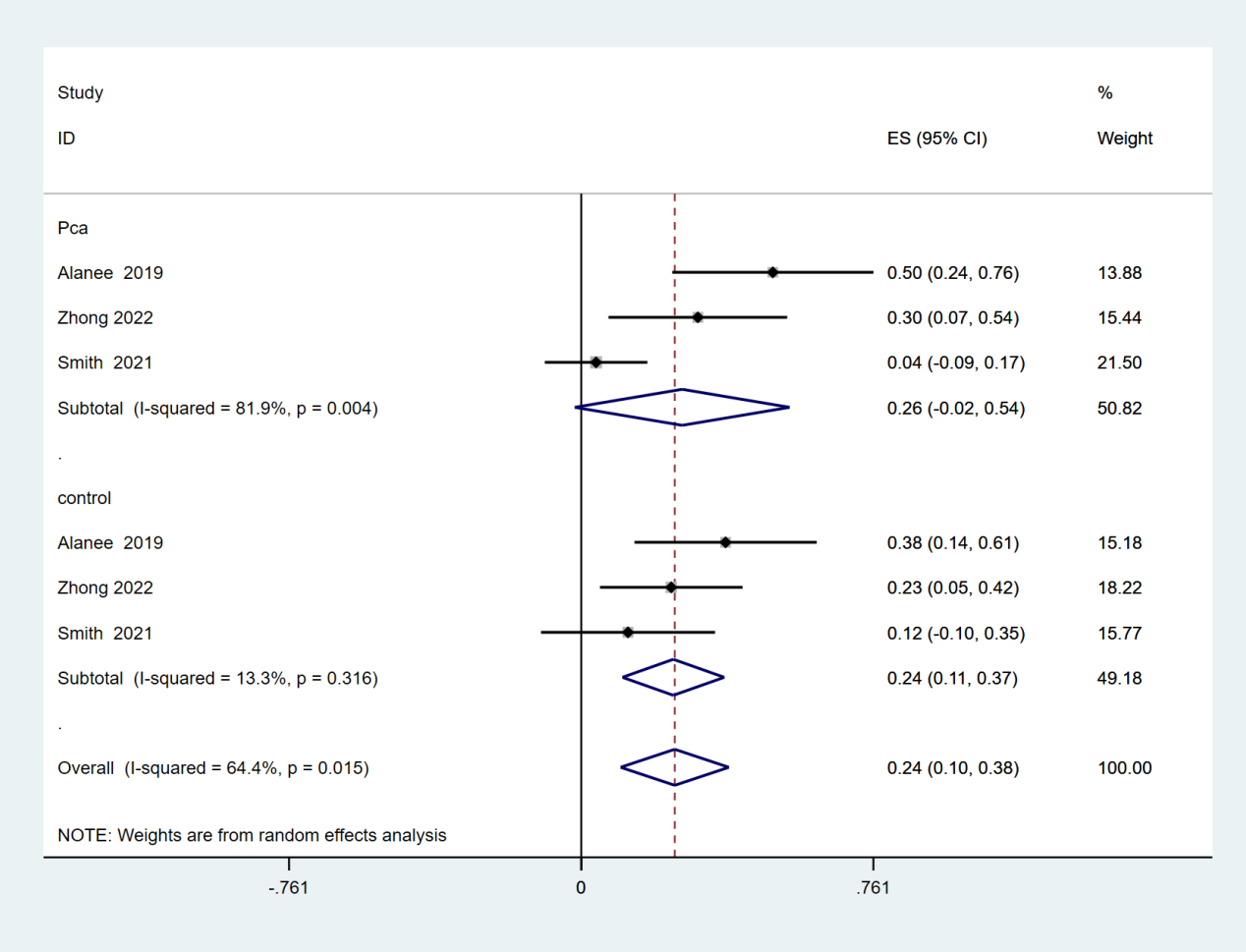


**Figure S36.** Forest plot of relative abundance of *Lactobacillales* in prostate patients and controls.


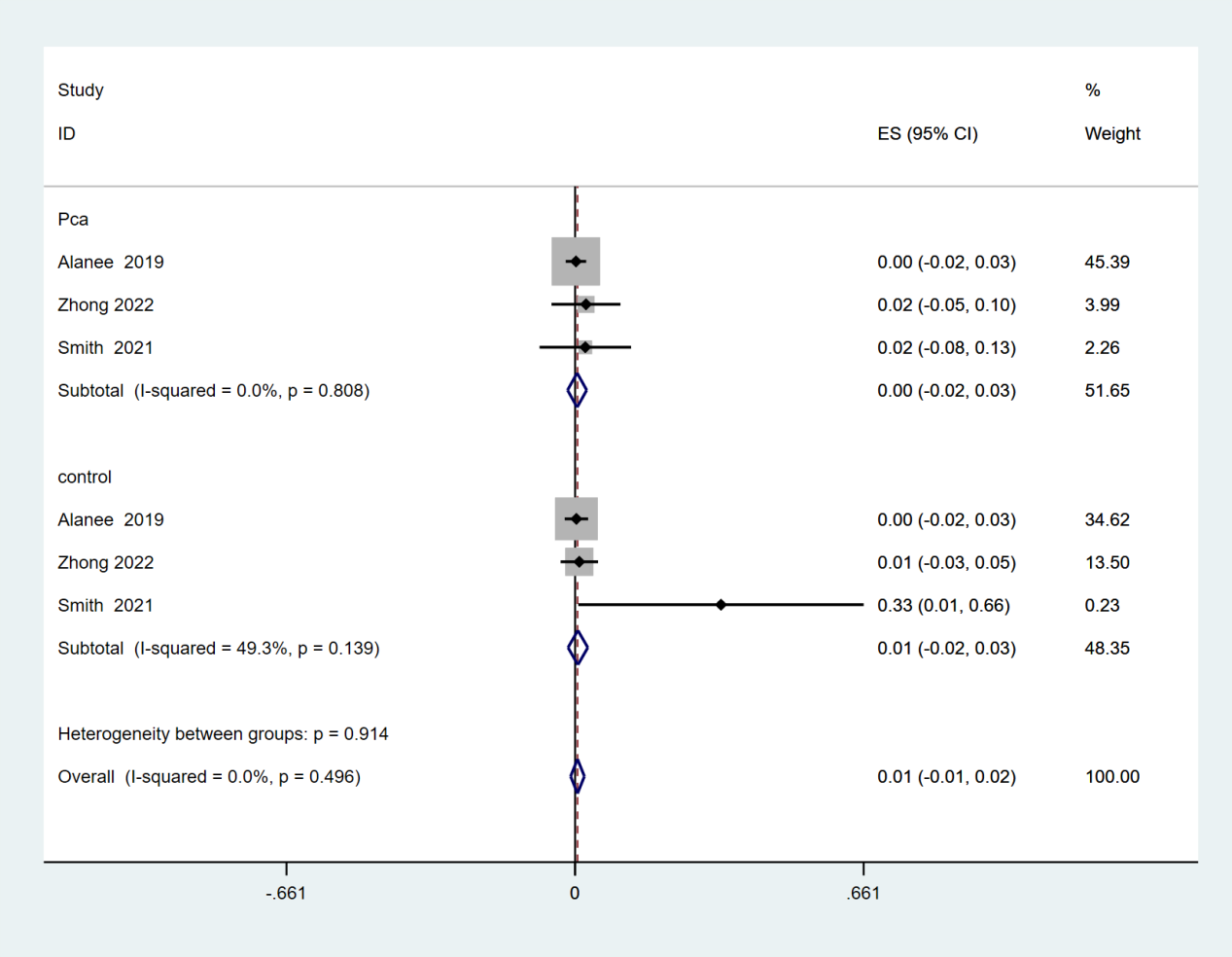


**Figure S37.** Forest plot of relative abundance of *Clostridiales* in prostate patients and controls.


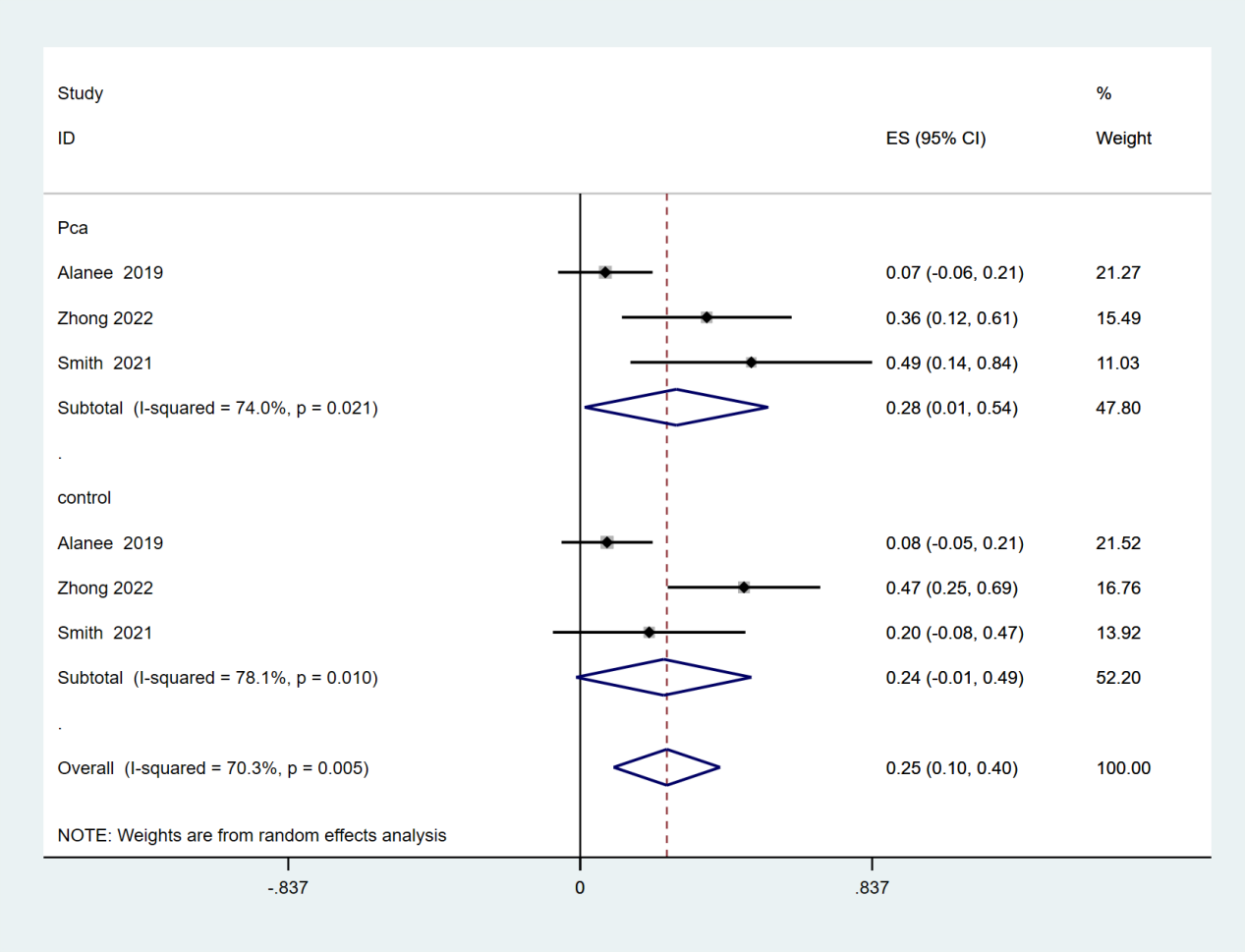


**Figure S38.** Forest plot of relative abundance of *Selenomonadales* in prostate patients and controls.


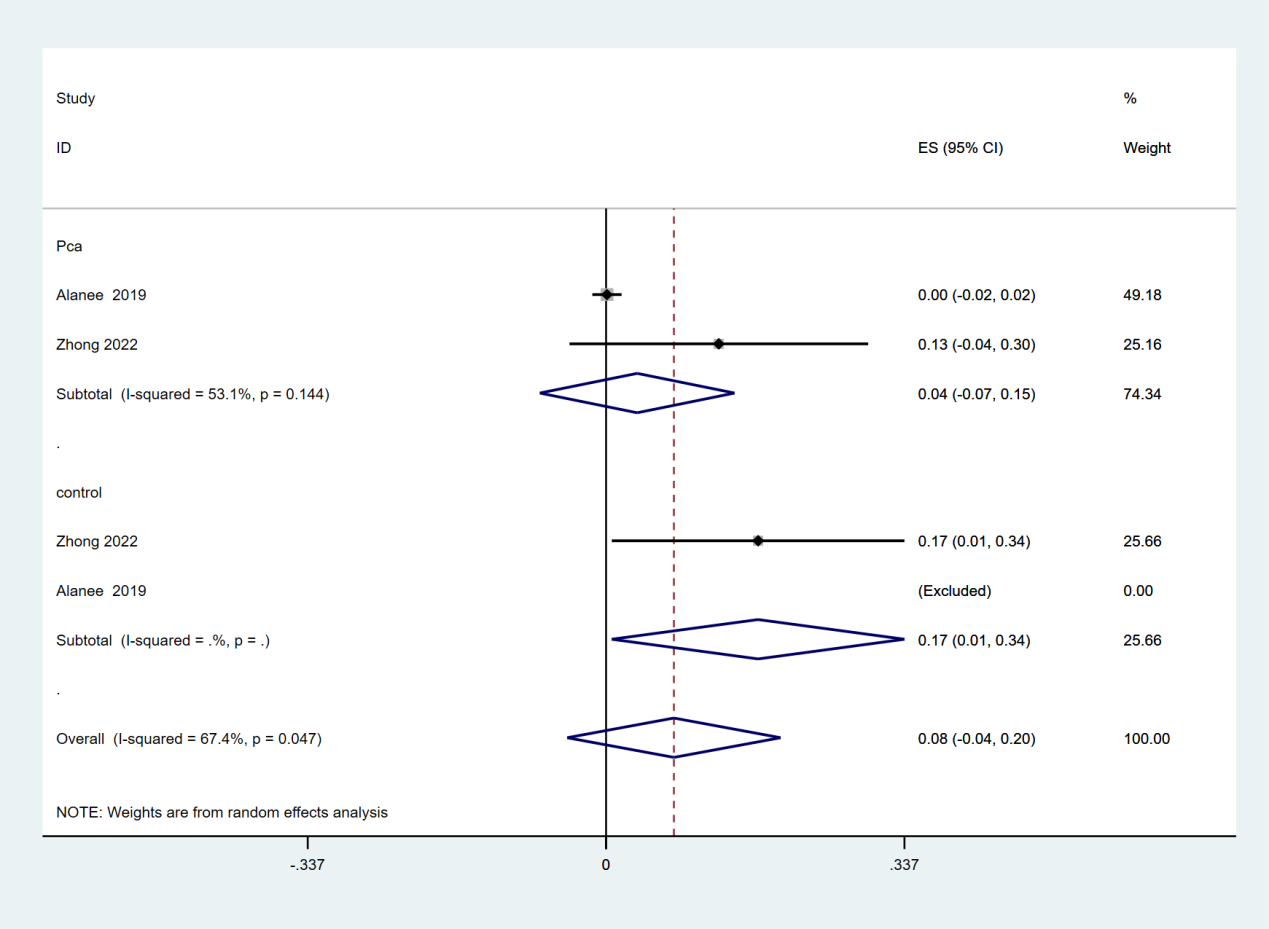


**Figure S39.** Forest plot of relative abundance of *Enterobacteriales* in prostate patients and controls.


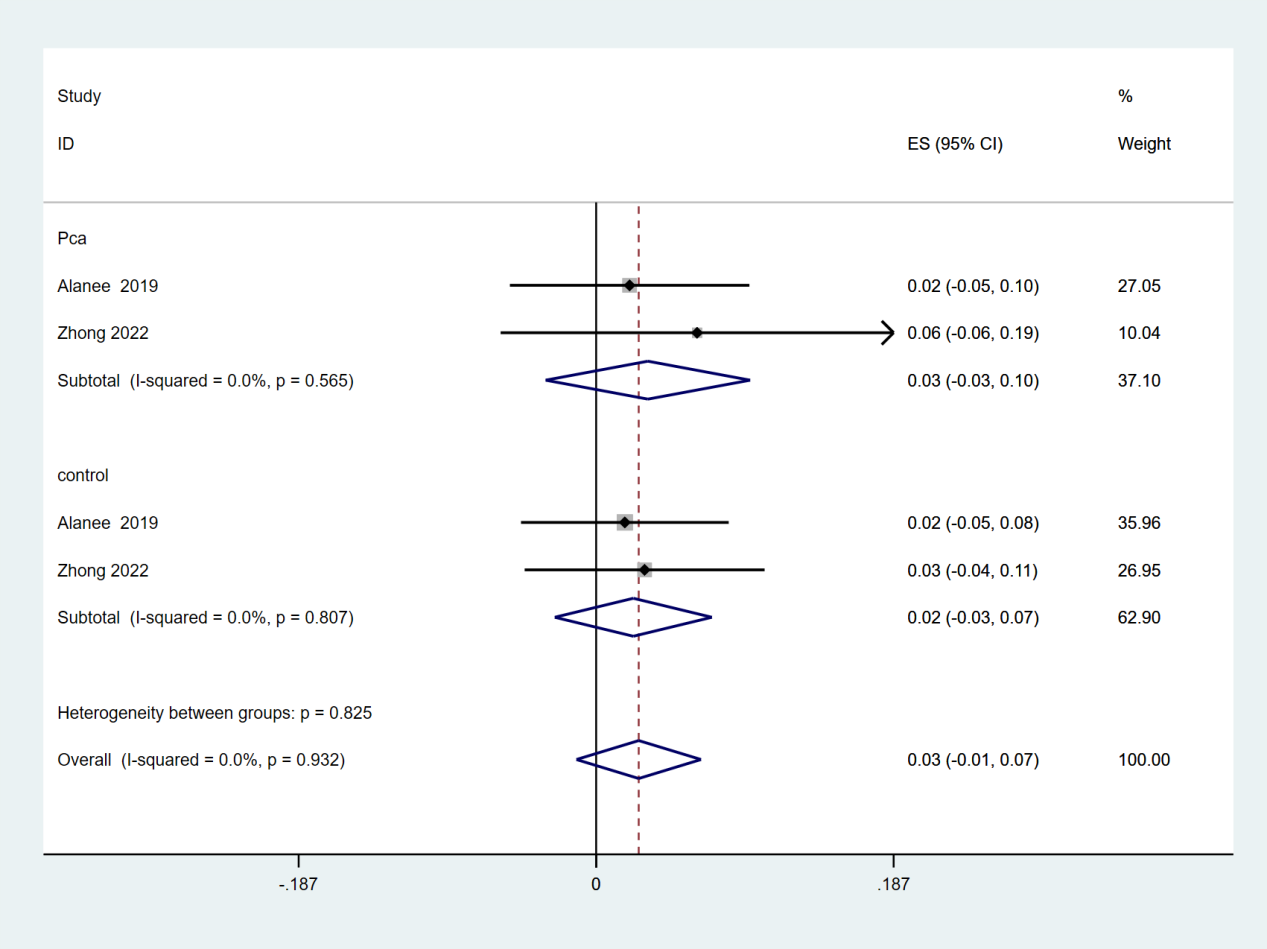


**Figure S40.** Forest plot of relative abundance of *Actinomycetales* in prostate patients and controls.


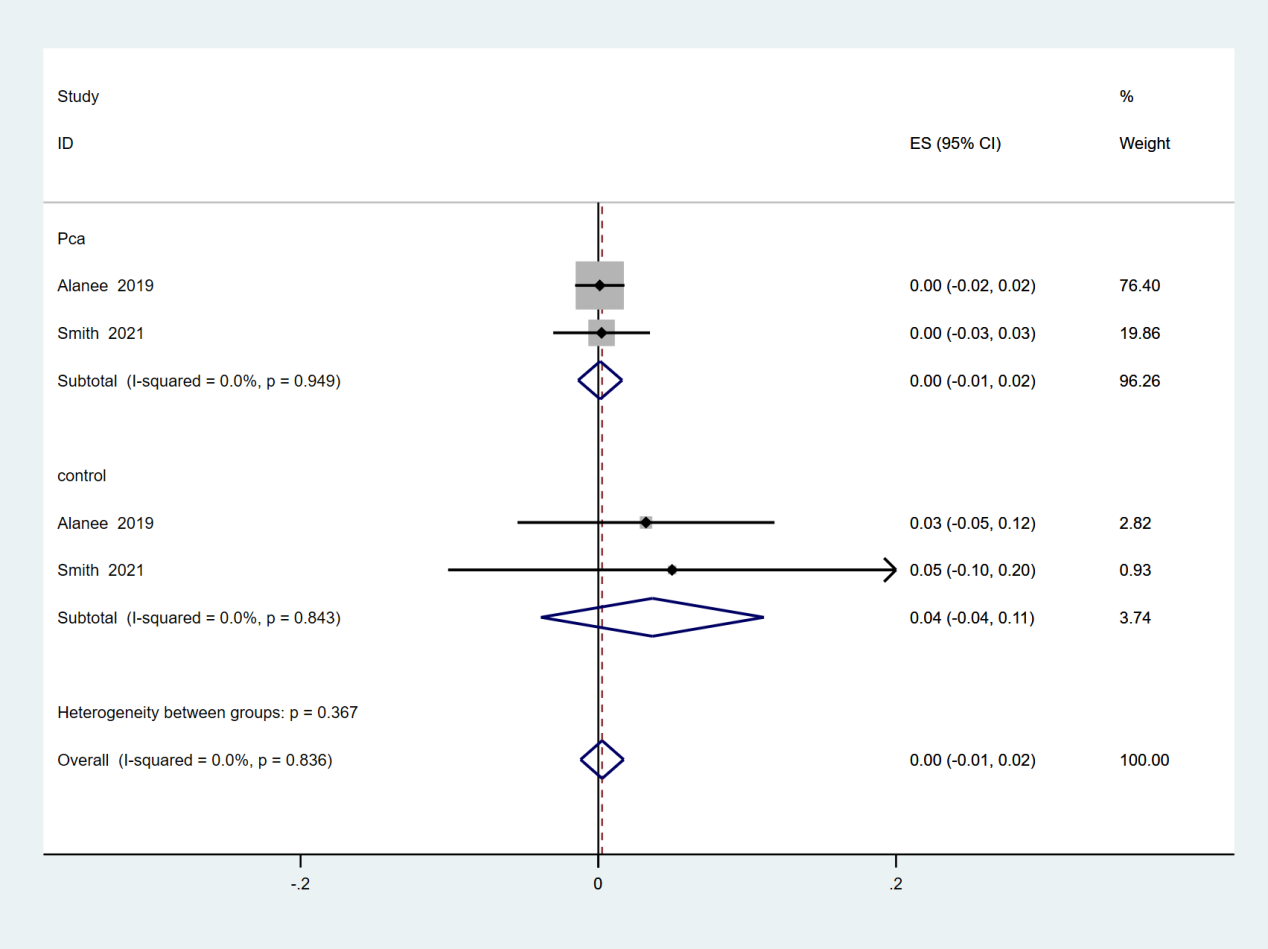


**Figure S41.** Forest plot of relative abundance of *Bifidobacteriales* in prostate patients and controls.


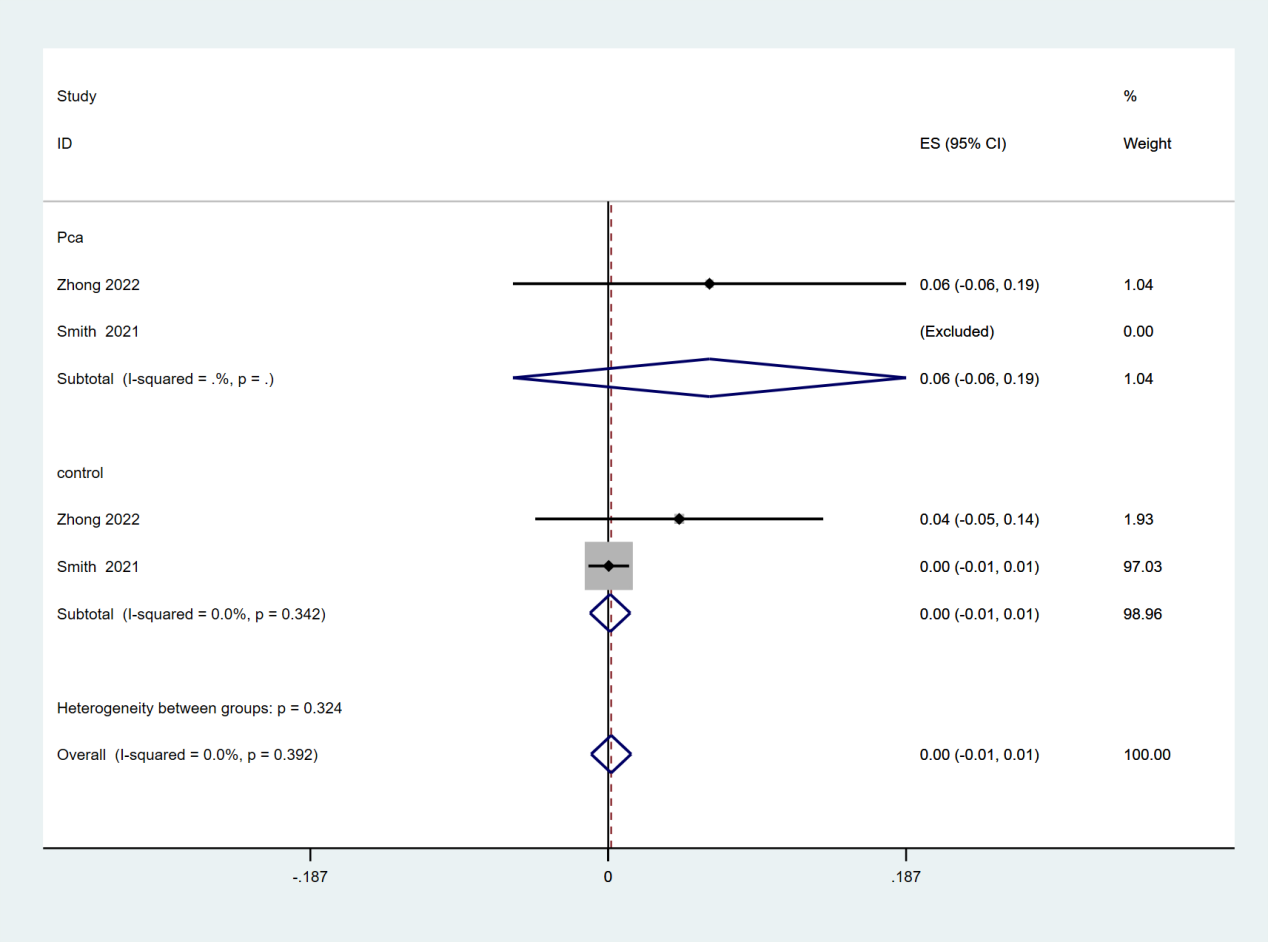


**Figure S42.** Forest plot of relative abundance of *Coriobacteriales* in prostate patients and controls.


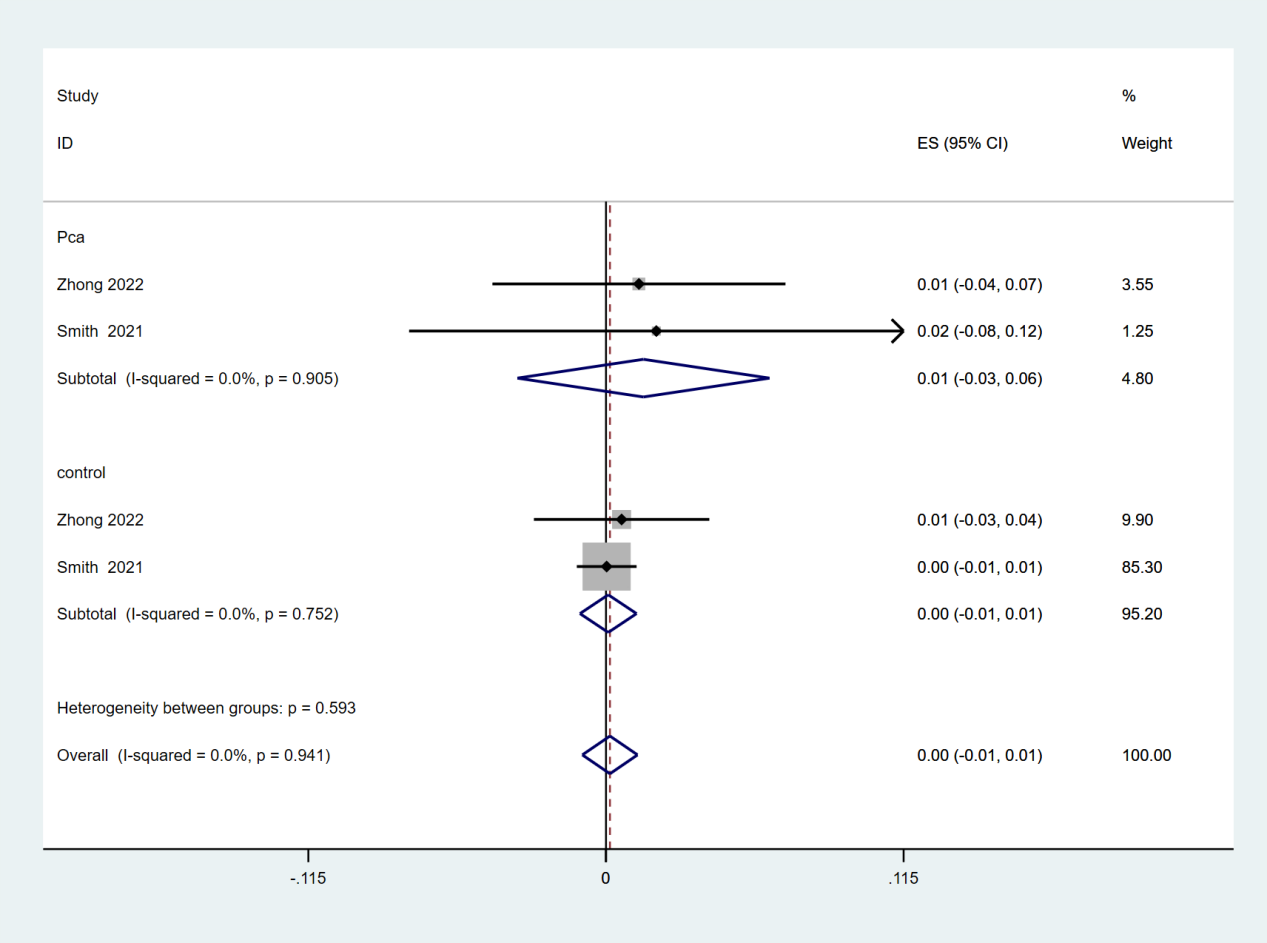

Supplement: Supplementary file 3 — Supplementary Material 3. [file 12885_2024_12018_MOESM3_ESM.zip › Additional file 3/Figure S35-42. Forest plot of relative abundance of GM in at order level.docx]
