# Supplementary material for: Gut microbiota in patients with prostate cancer: a systematic review and meta-analysis
Source: BMC Cancer. 2024 Feb 24;24:261. doi: 10.1186/s12885-024-12018-x (PMC10893726; doi:10.1186/s12885-024-12018-x)

**Figure S46.** Forest plot of relative abundance of *Corynebacteriaceae* in prostate patients and controls.


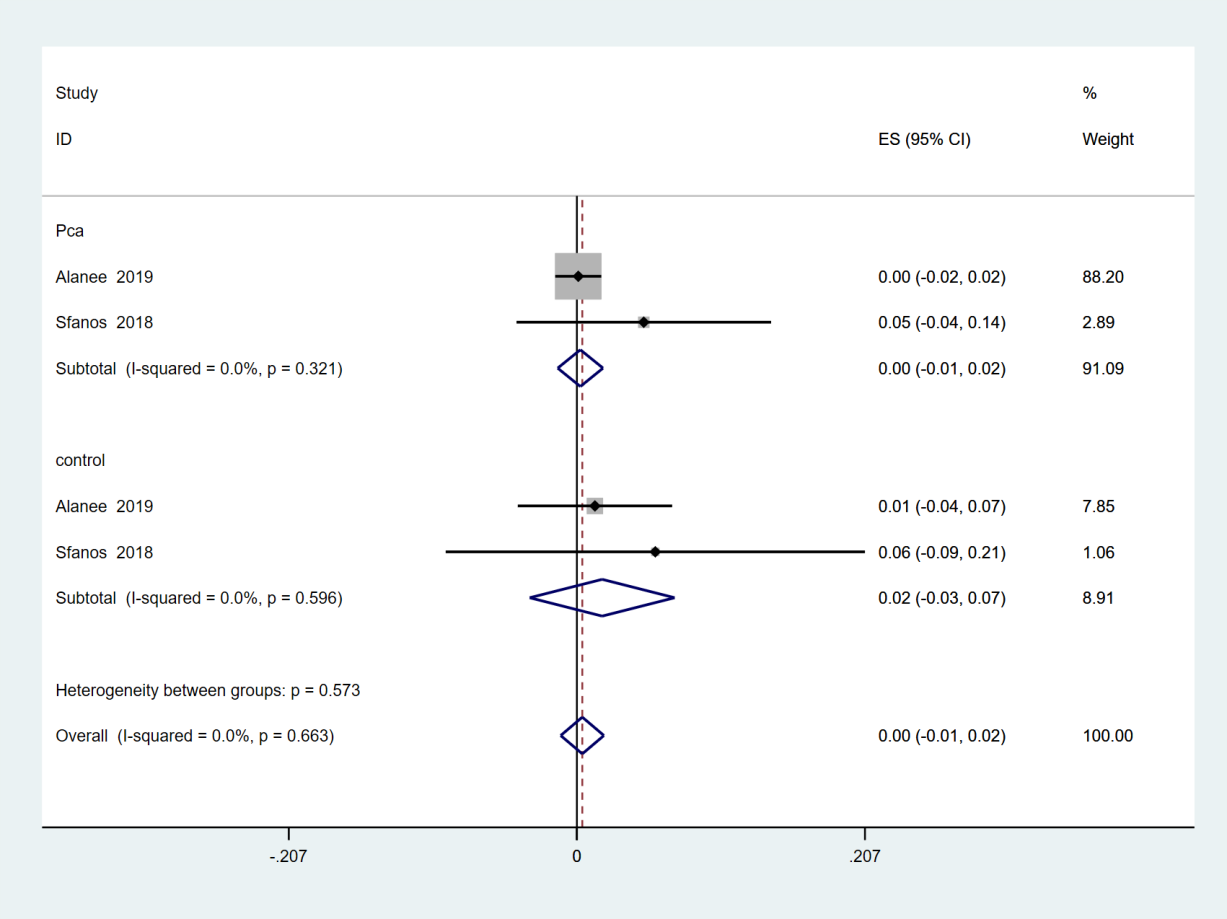


**Figure S47.** Forest plot of relative abundance of *Prevotellaceae* in prostate patients and controls.


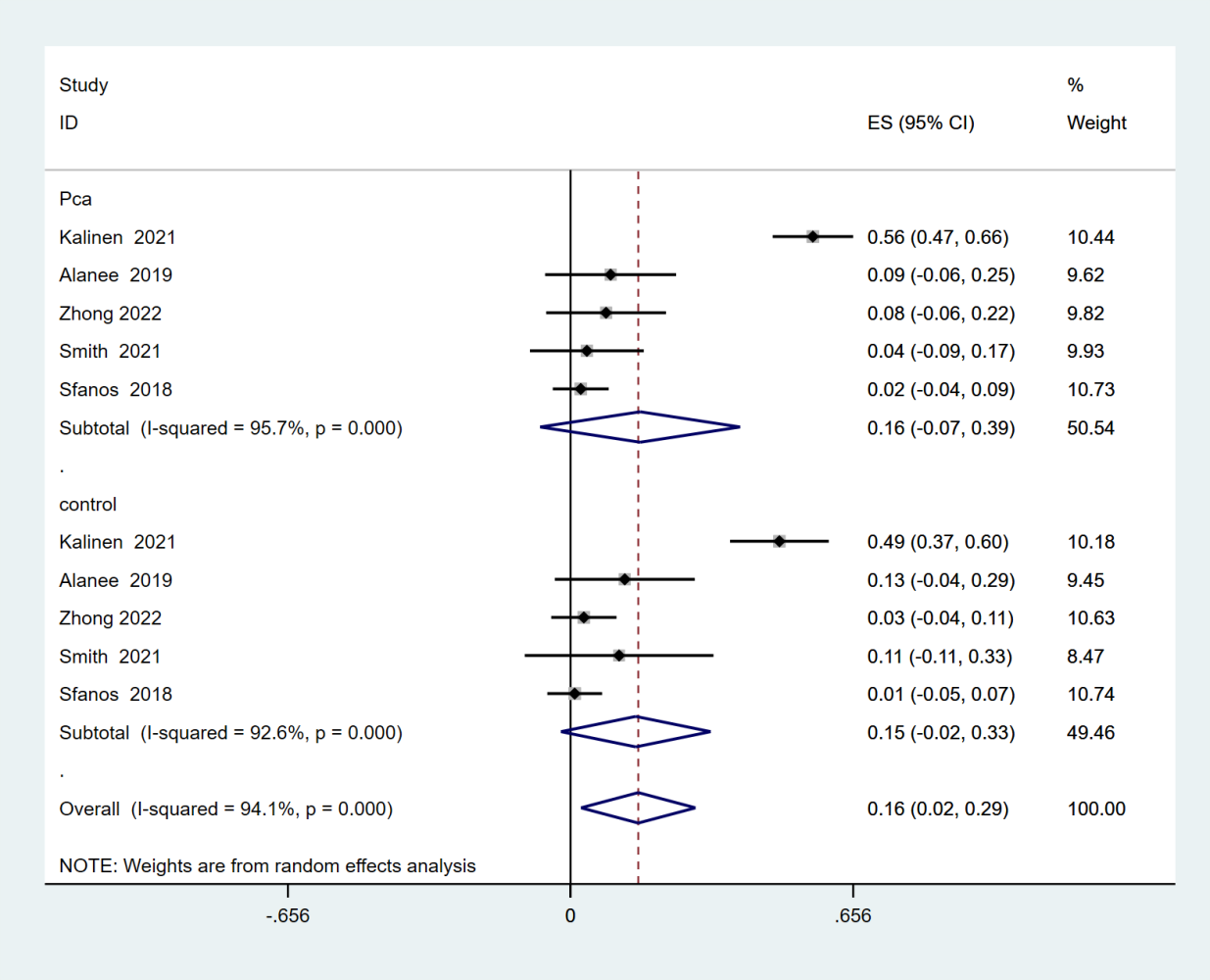


**Figure S48.** Forest plot of relative abundance of *Streptococcaceae* in prostate patients and controls.


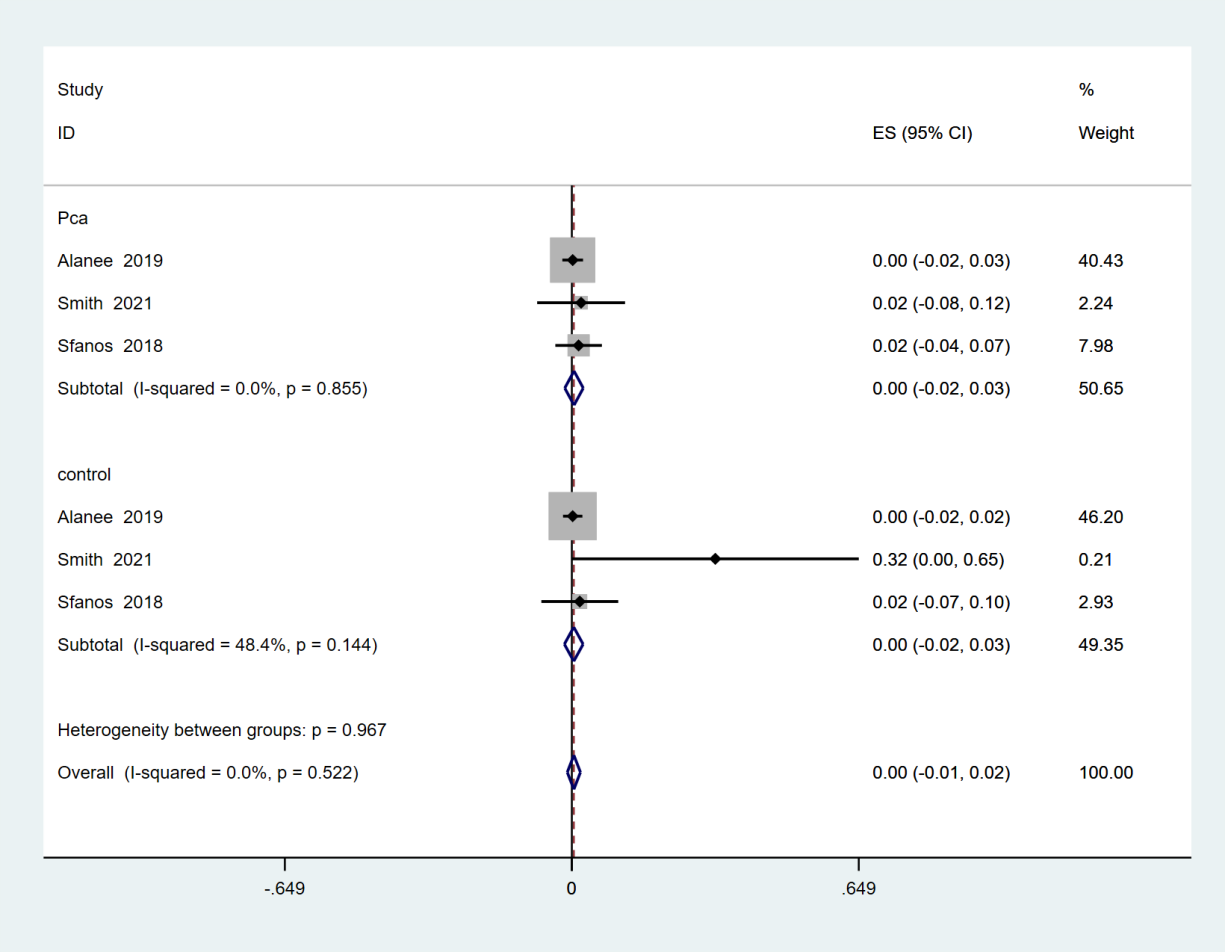


**Figure S49.** Forest plot of relative abundance of *Lachnospiraceae* in prostate patients and controls.


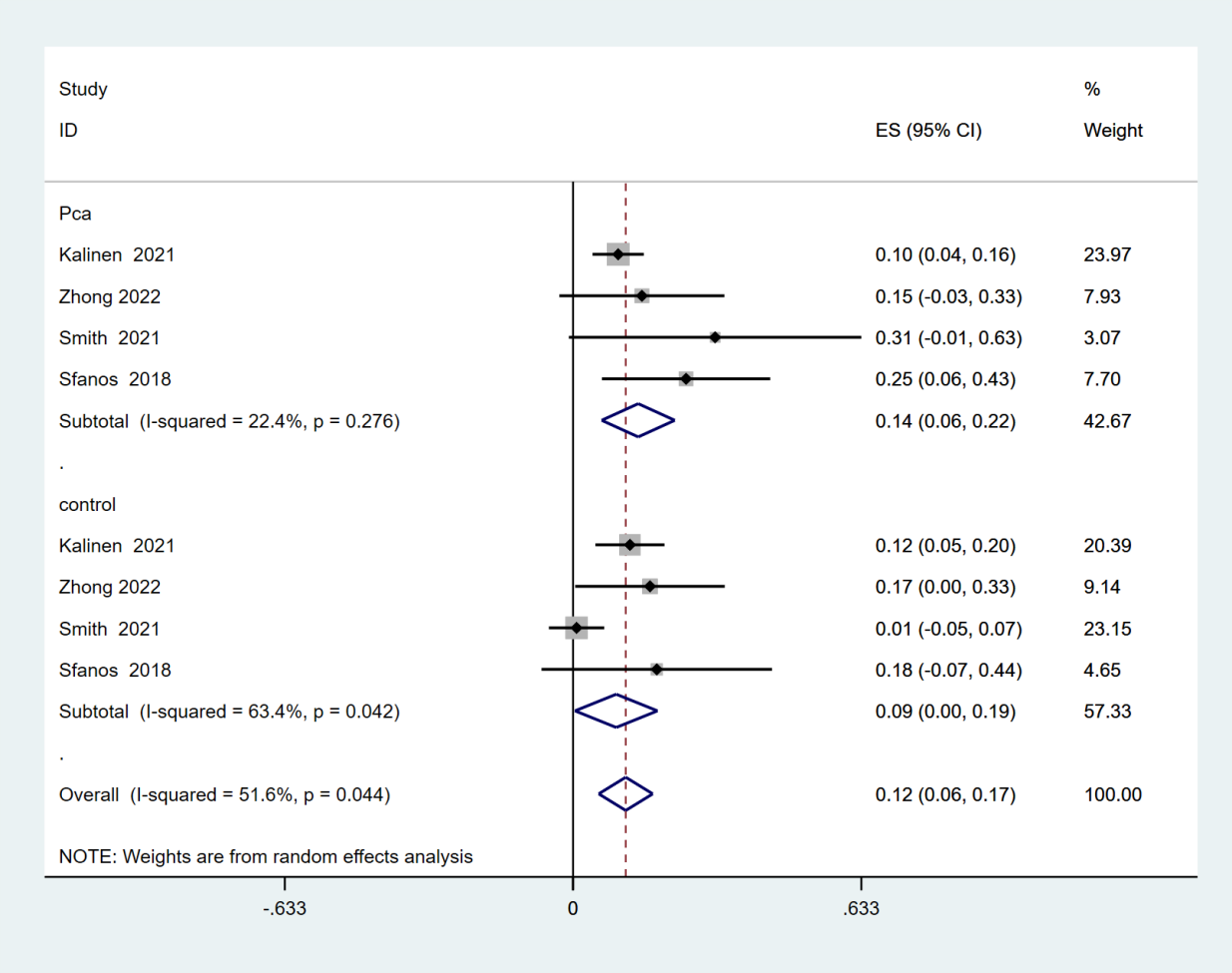


**Figure S50.** Forest plot of relative abundance of *Ruminococcaceae* in prostate patients and controls.


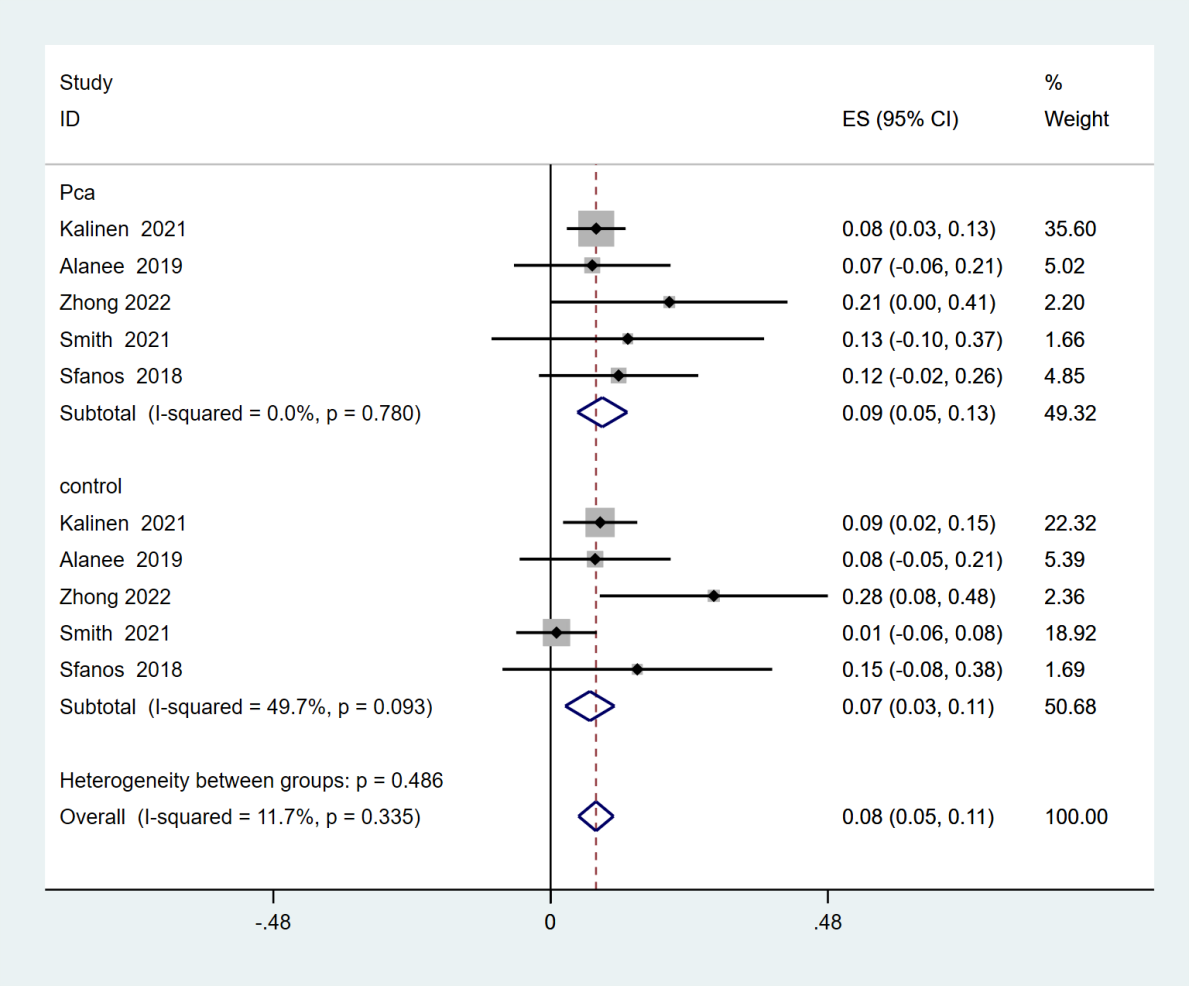


**Figure S51.** Forest plot of relative abundance of *Erysipelotrichaceae* in prostate patients and controls.


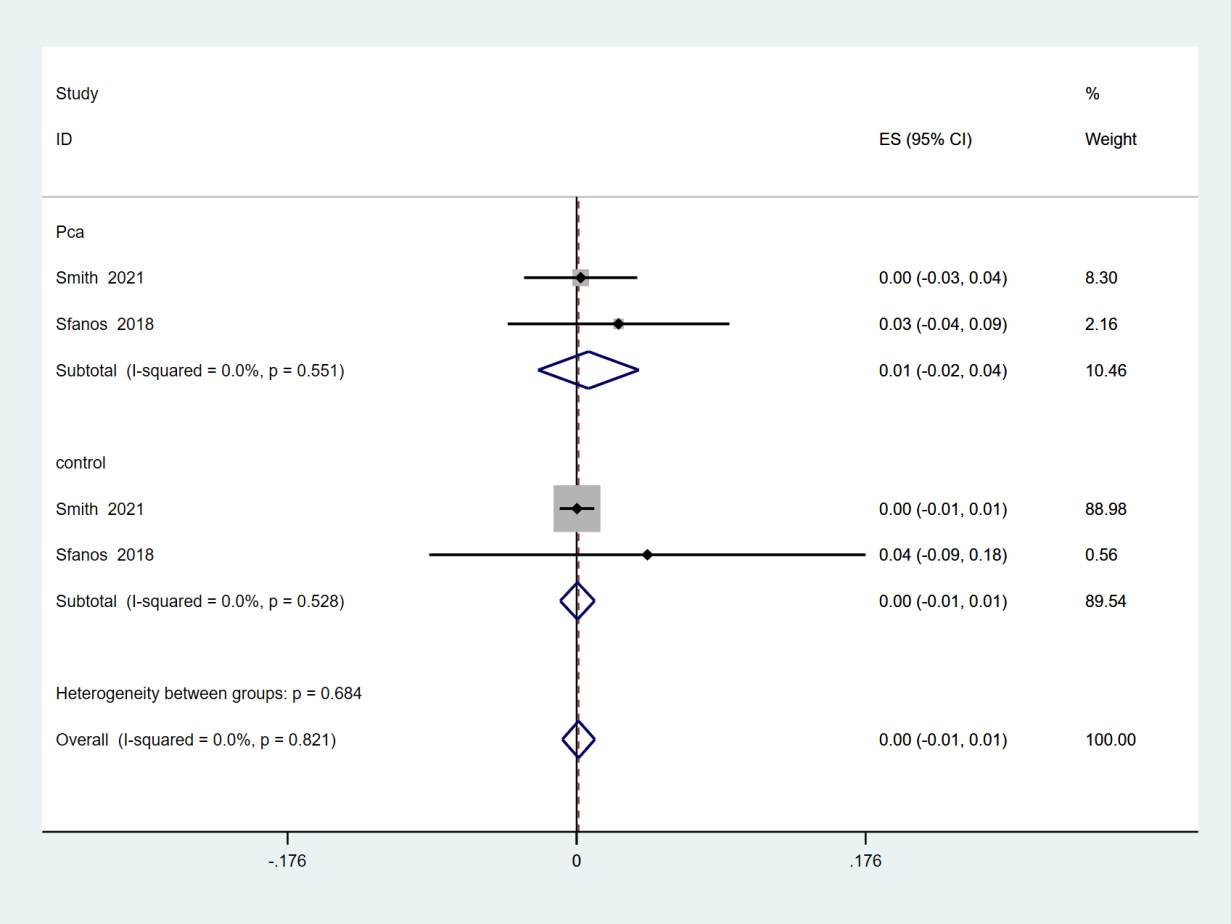


**Figure S52.** Forest plot of relative abundance of *Acidaminococcaceae* in prostate patients and controls.


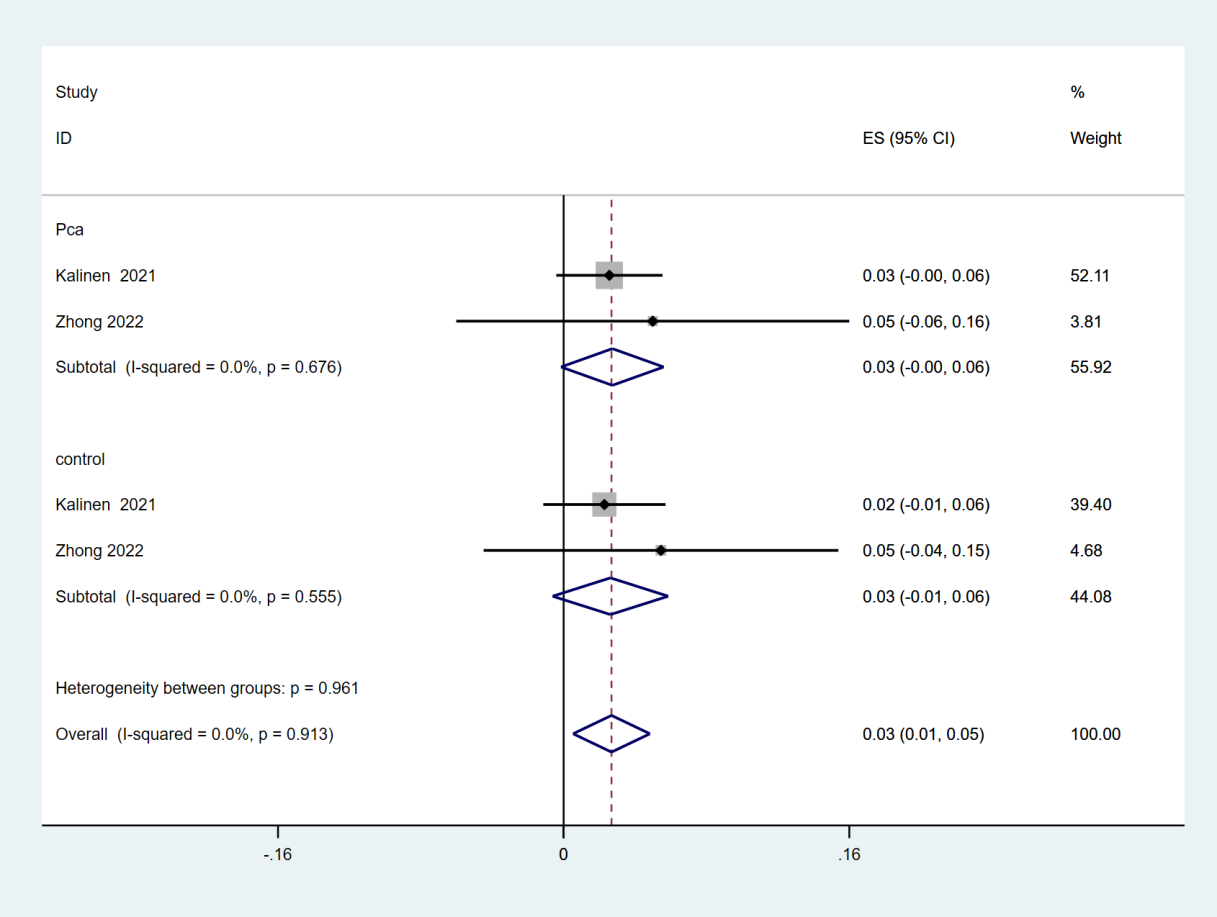


**Figure S53.** Forest plot of relative abundance of *Veillonellaceae* in prostate patients and controls.


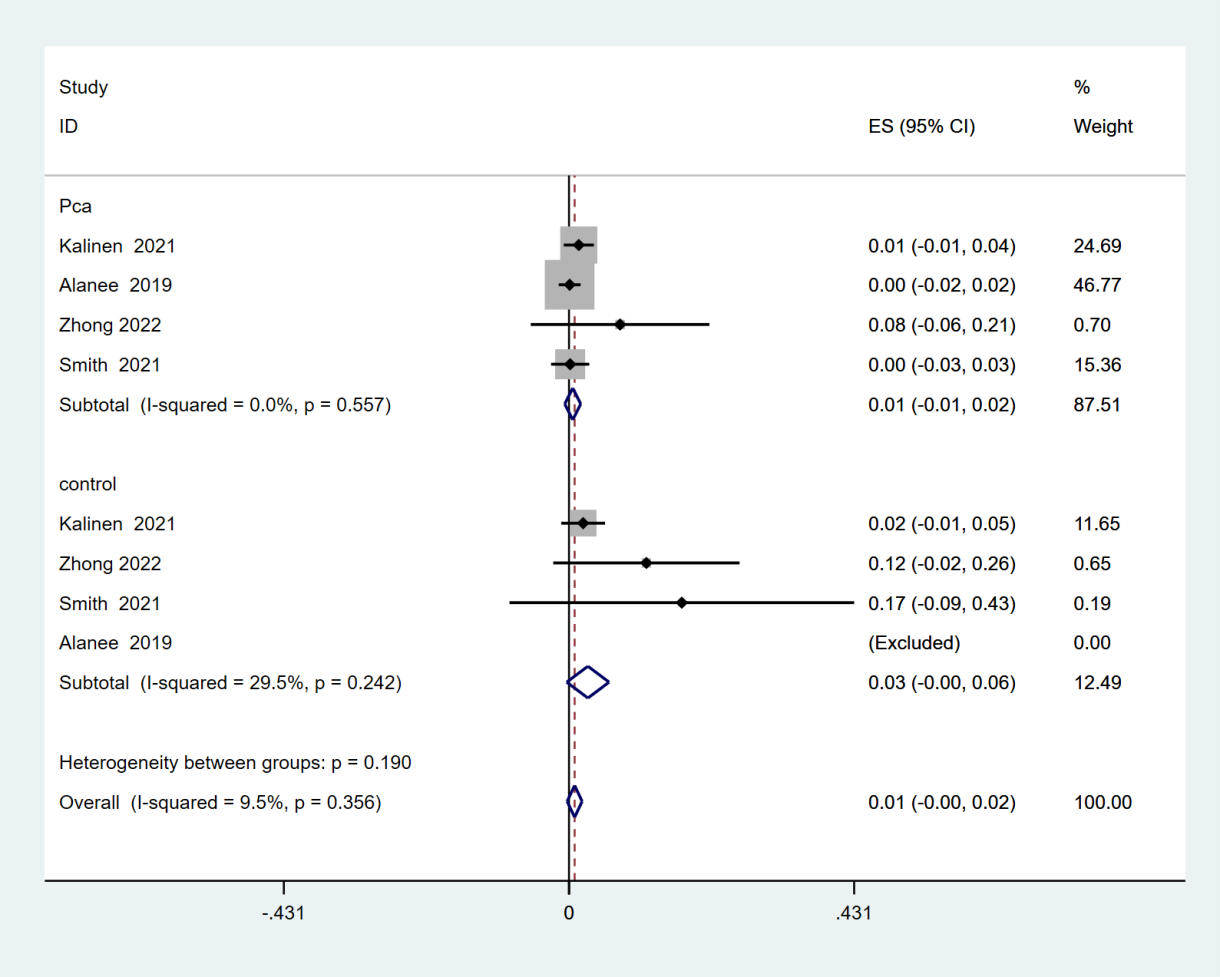


**Figure S54.** Forest plot of relative abundance of *Burkholderiaceae* in prostate patients and controls.


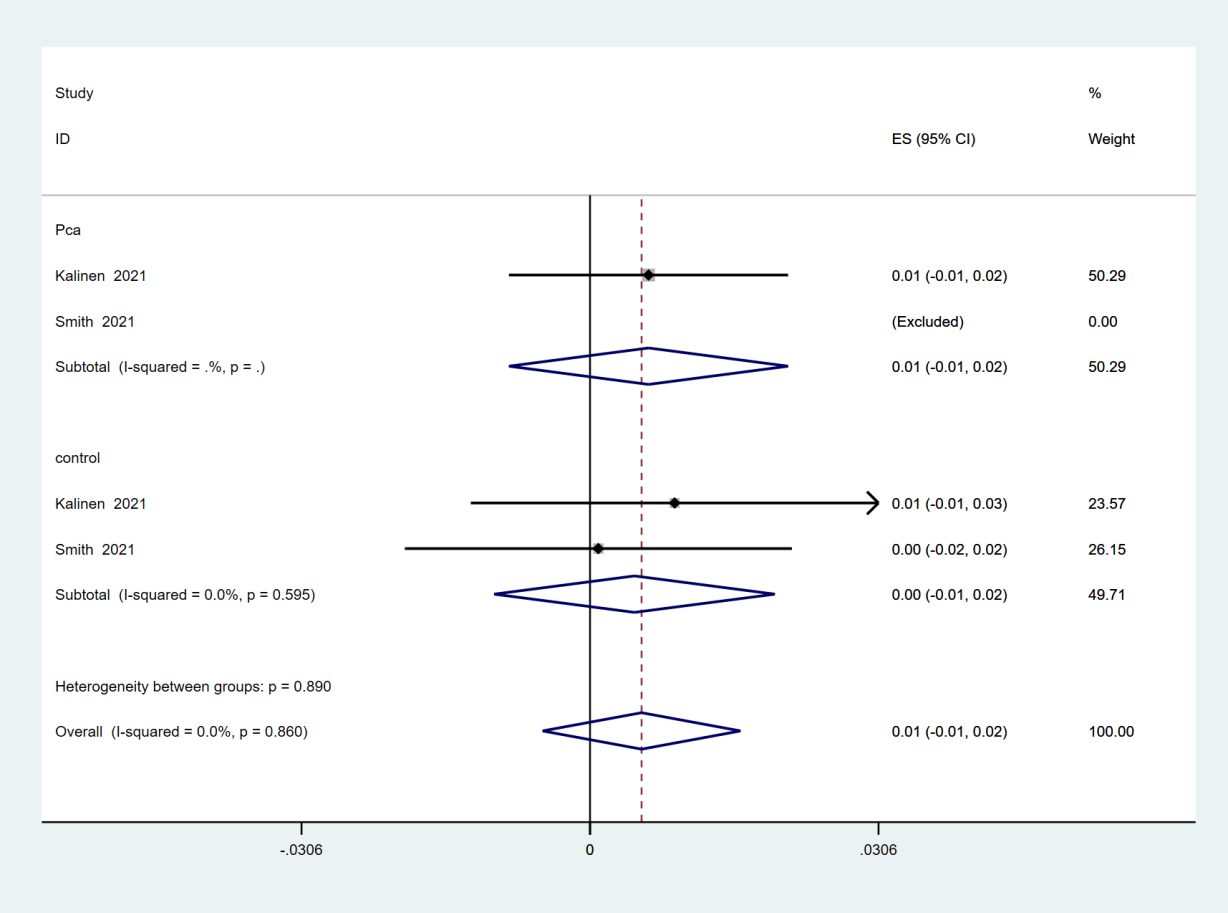


**Figure S55.** Forest plot of relative abundance of *Enterobacteriaceae* in prostate patients and controls.


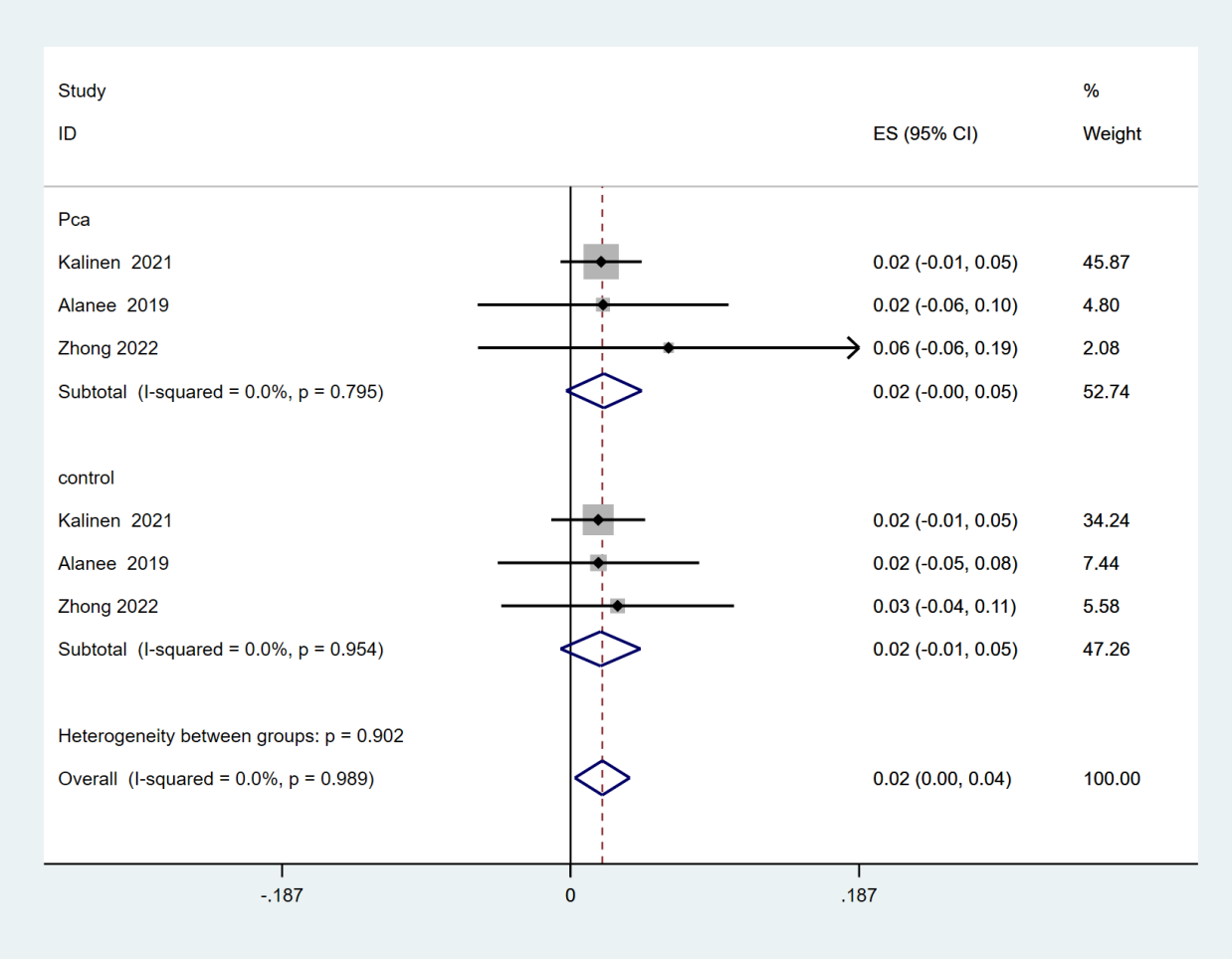


**Figure S56.** Forest plot of relative abundance of *Bacteroidaceae* in prostate patients and controls.


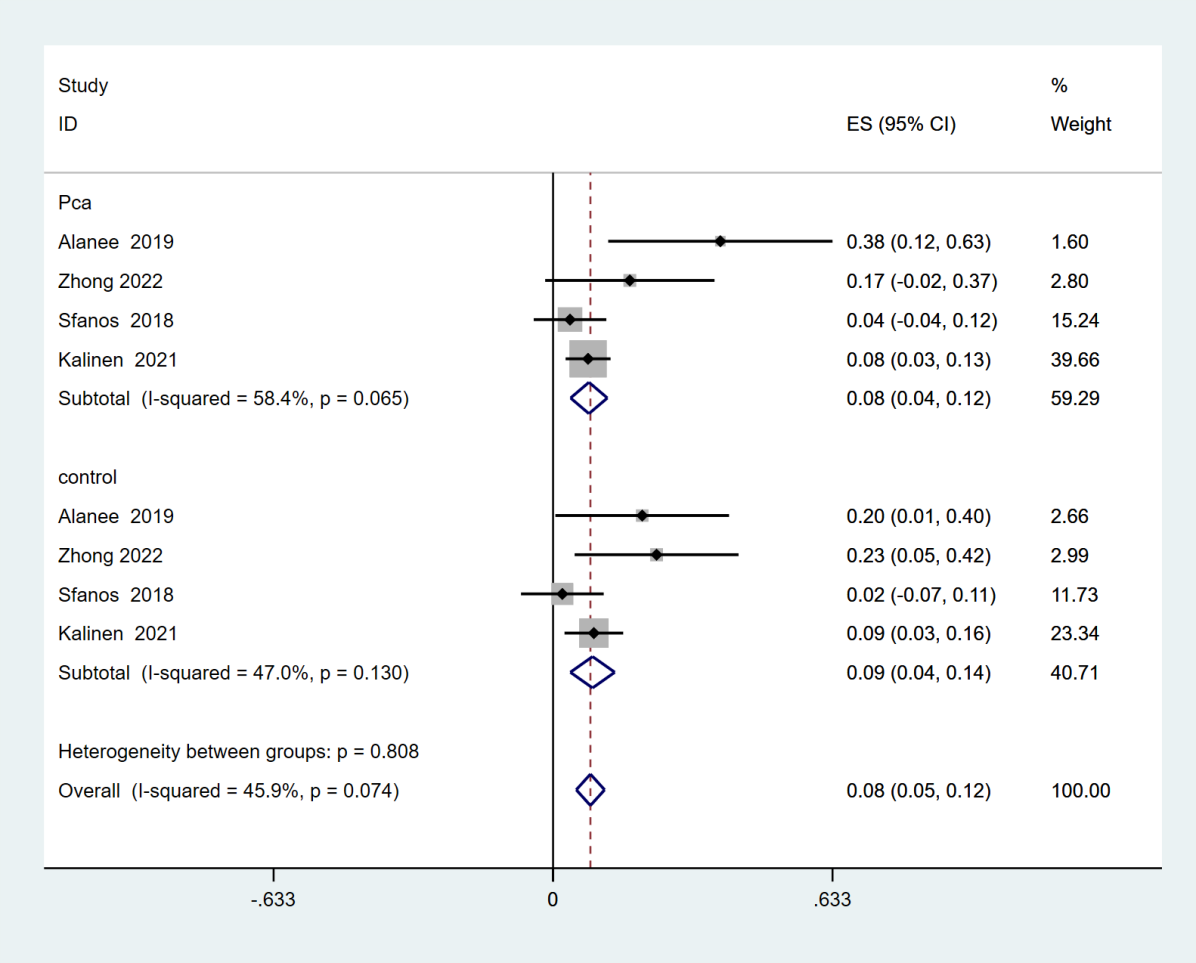


**Figure S57.** Forest plot of relative abundance of *Bifidobacteriaceae* in prostate patients and controls.


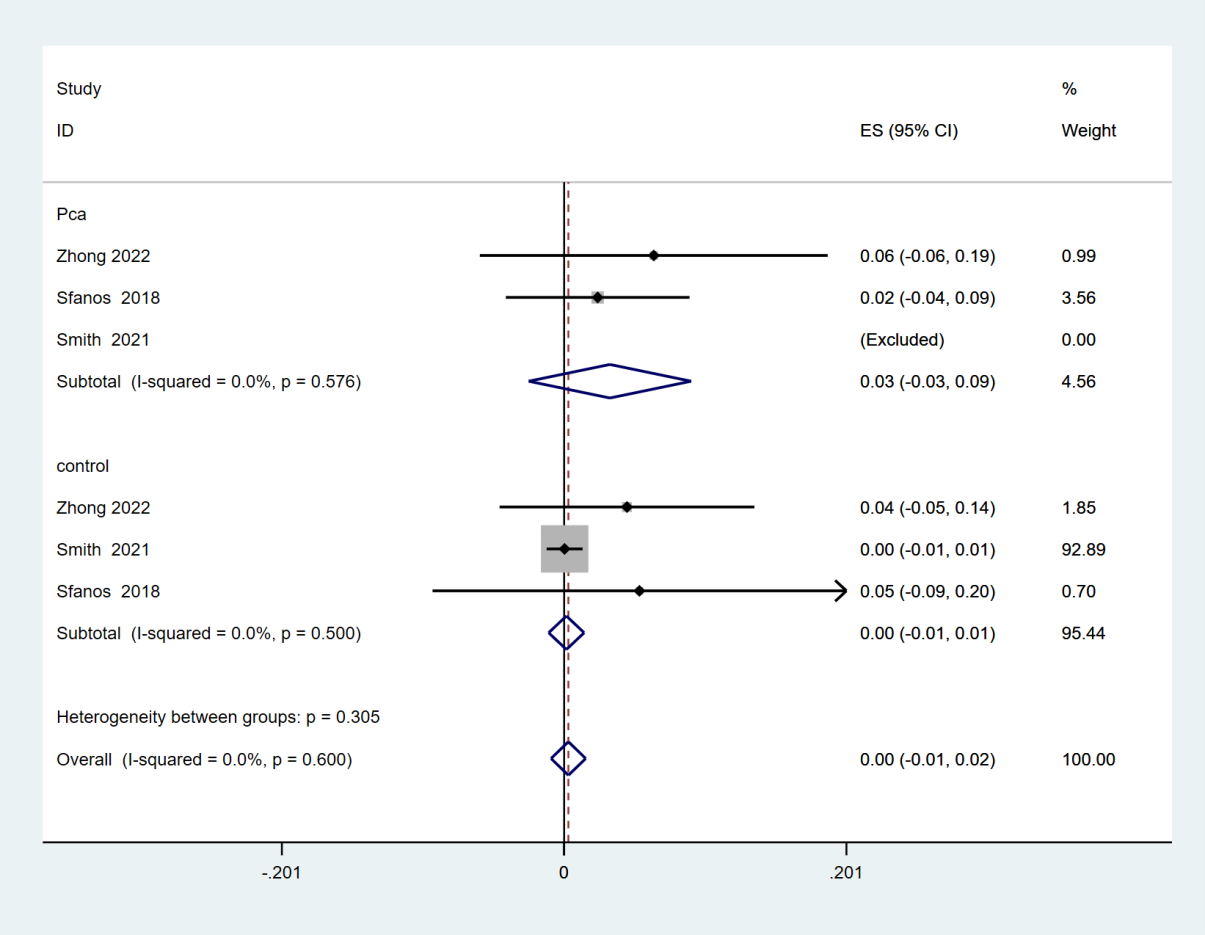


**Figure S58.** Forest plot of relative abundance of *Actinomycetaceae* in prostate patients and controls.


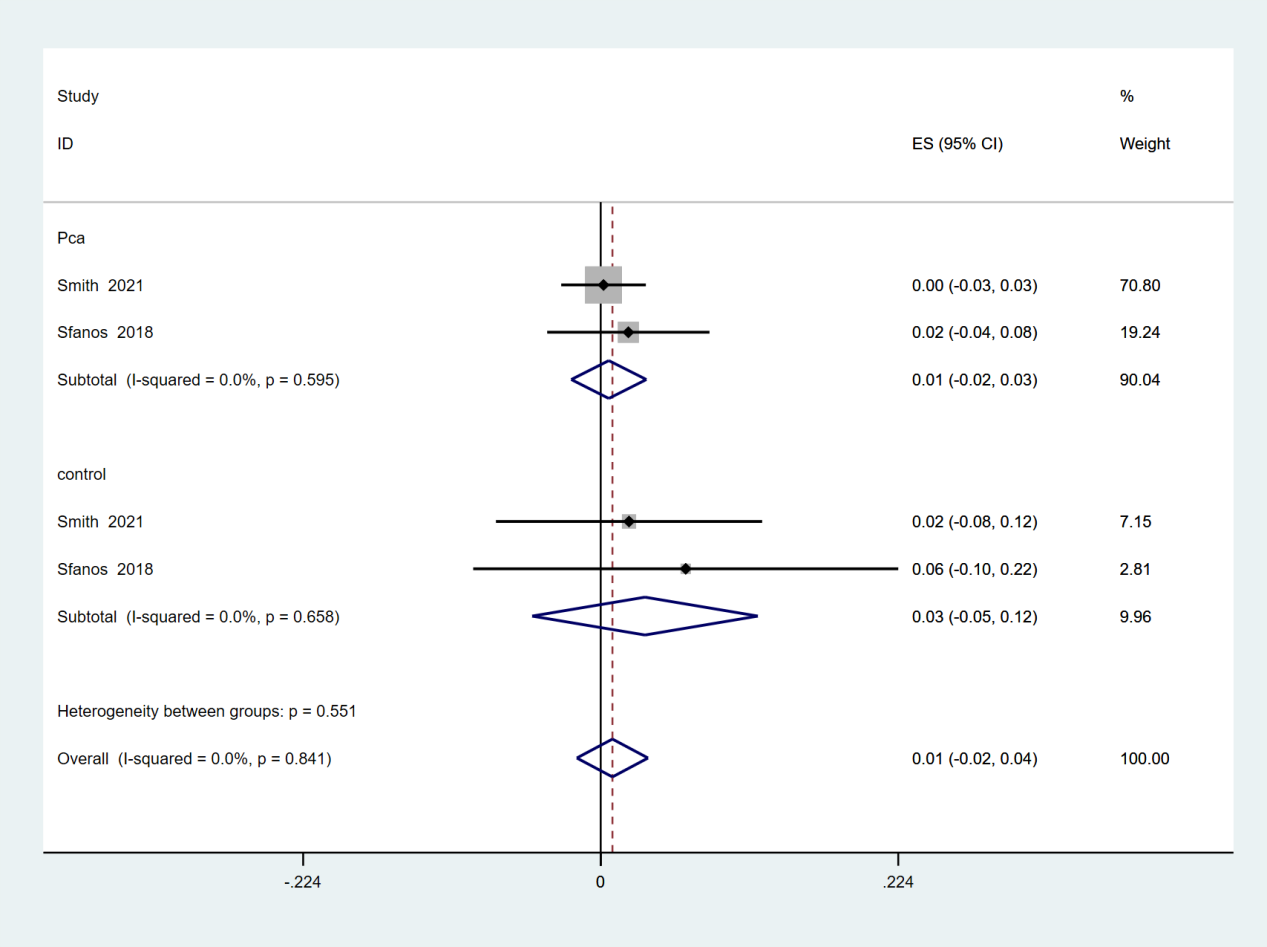

Supplement: Supplementary file 3 — Supplementary Material 3. [file 12885_2024_12018_MOESM3_ESM.zip › Additional file 3/Figure S46-58. Forest plot of relative abundance of GM in at family level.docx]
