# Supplementary material for: Gut microbiota in patients with prostate cancer: a systematic review and meta-analysis
Source: BMC Cancer. 2024 Feb 24;24:261. doi: 10.1186/s12885-024-12018-x (PMC10893726; doi:10.1186/s12885-024-12018-x)

**Figure S5.** Forest plot of relative abundance of *Actinobacteria* in prostate patients and controls.


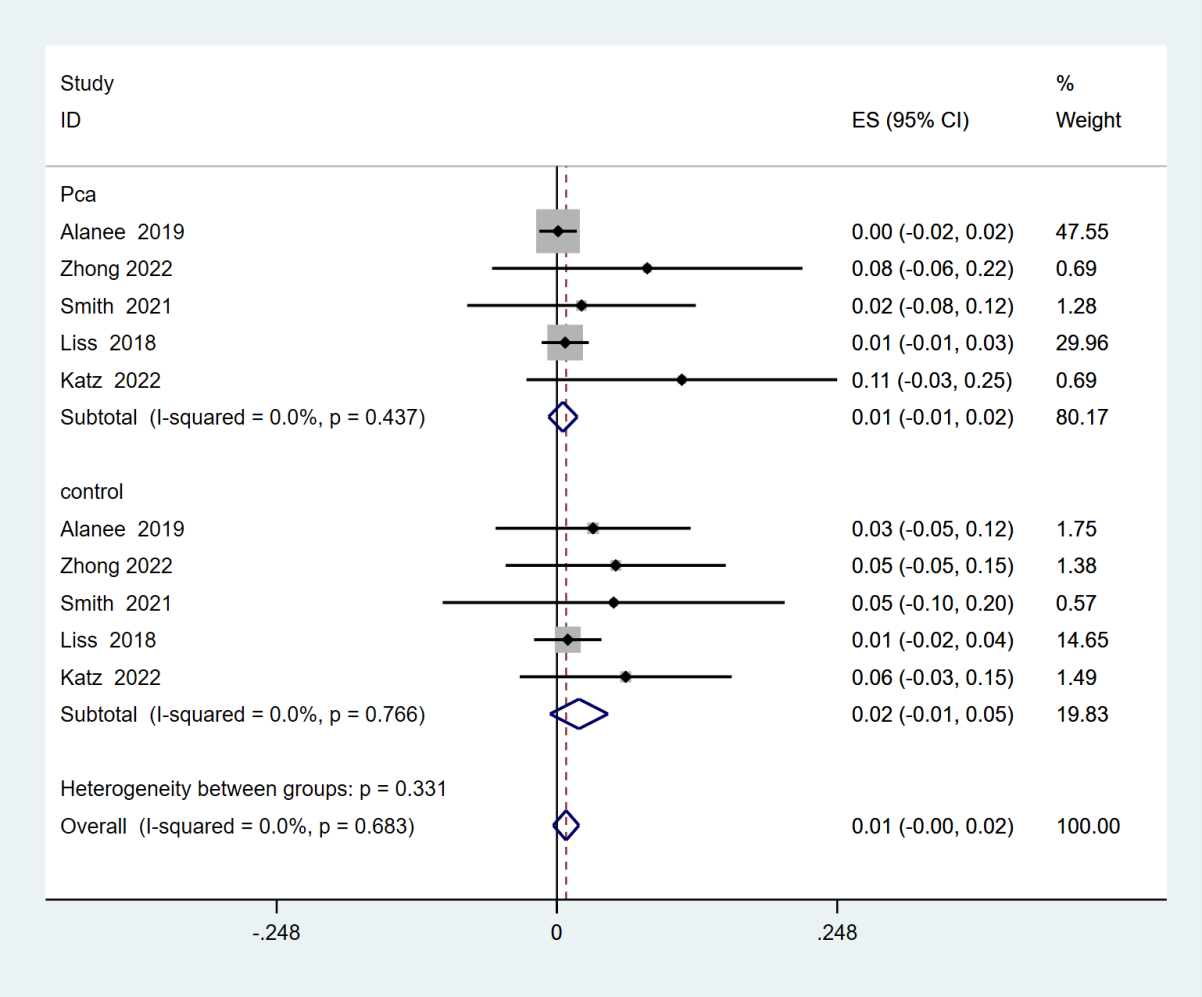


**Figure S6.** Forest plot of relative abundance of *Bacteroidetes* in prostate patients and controls.


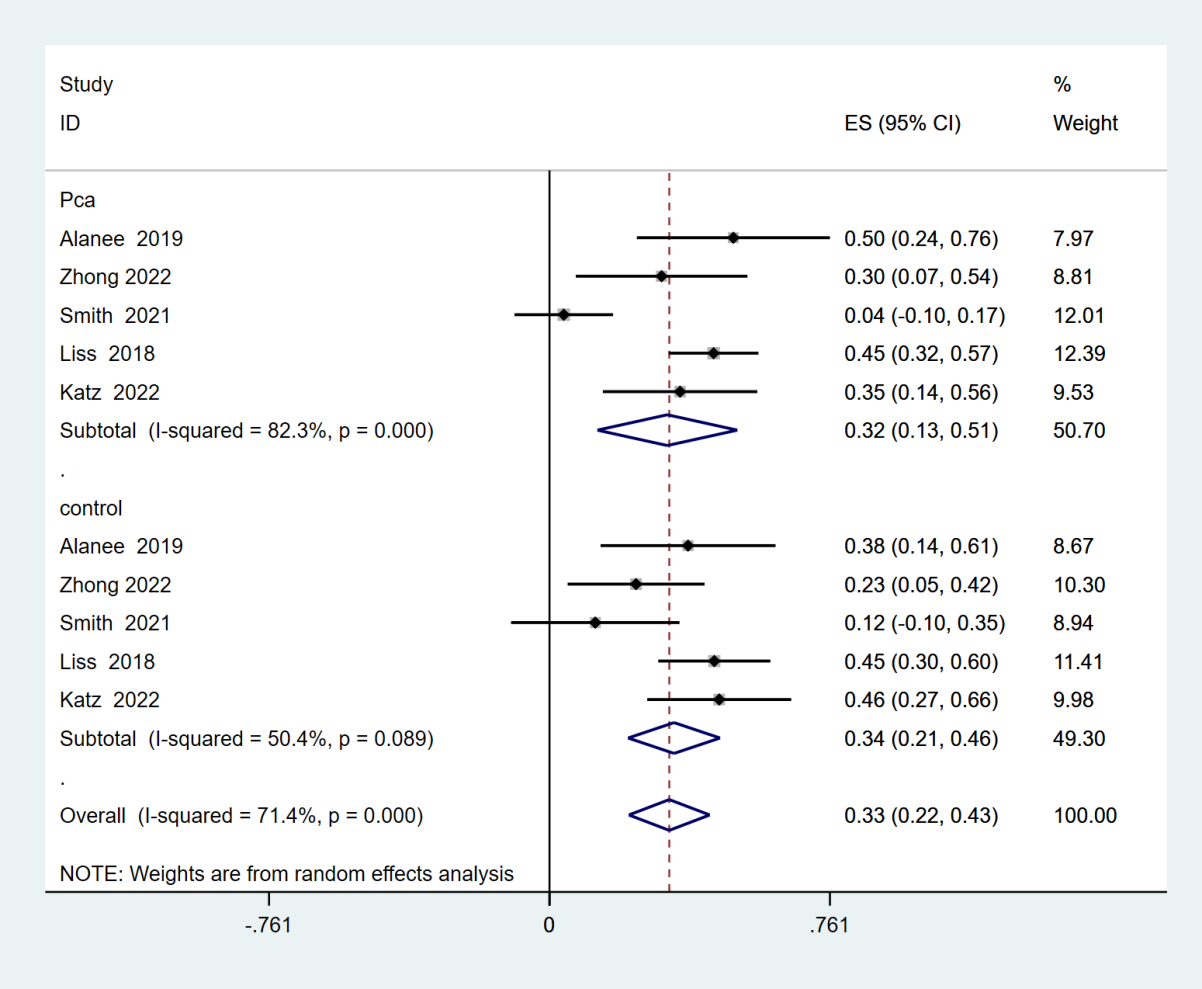


**Figure S7.** Forest plot of relative abundance of *Cyanobacteria* in prostate patients and controls.


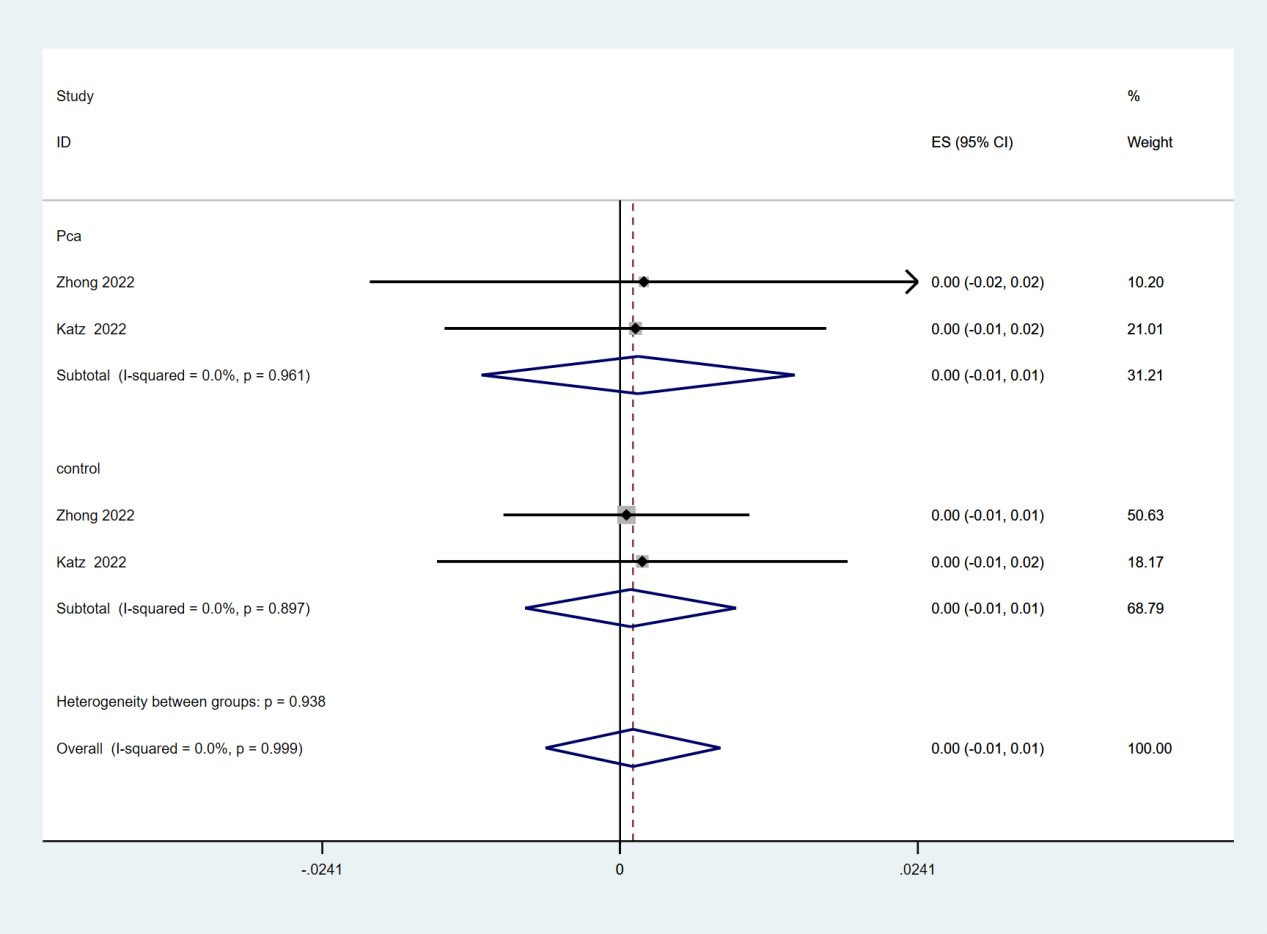


**Figure S8.** Forest plot of relative abundance of *Firmicutes* in prostate patients and controls.


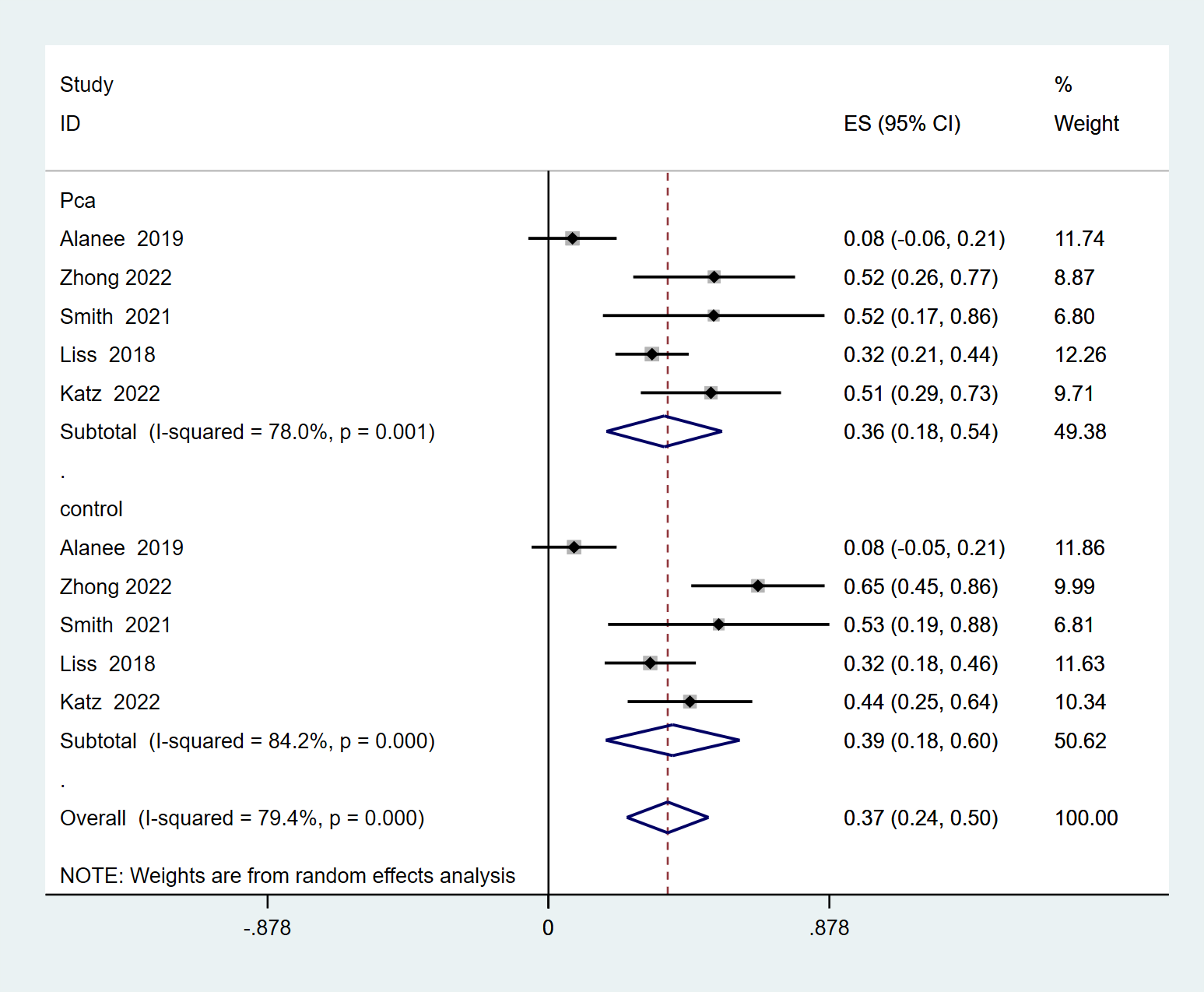


**Figure S9.** Forest plot of relative abundance of *Proteobacteria* in prostate patients and controls.


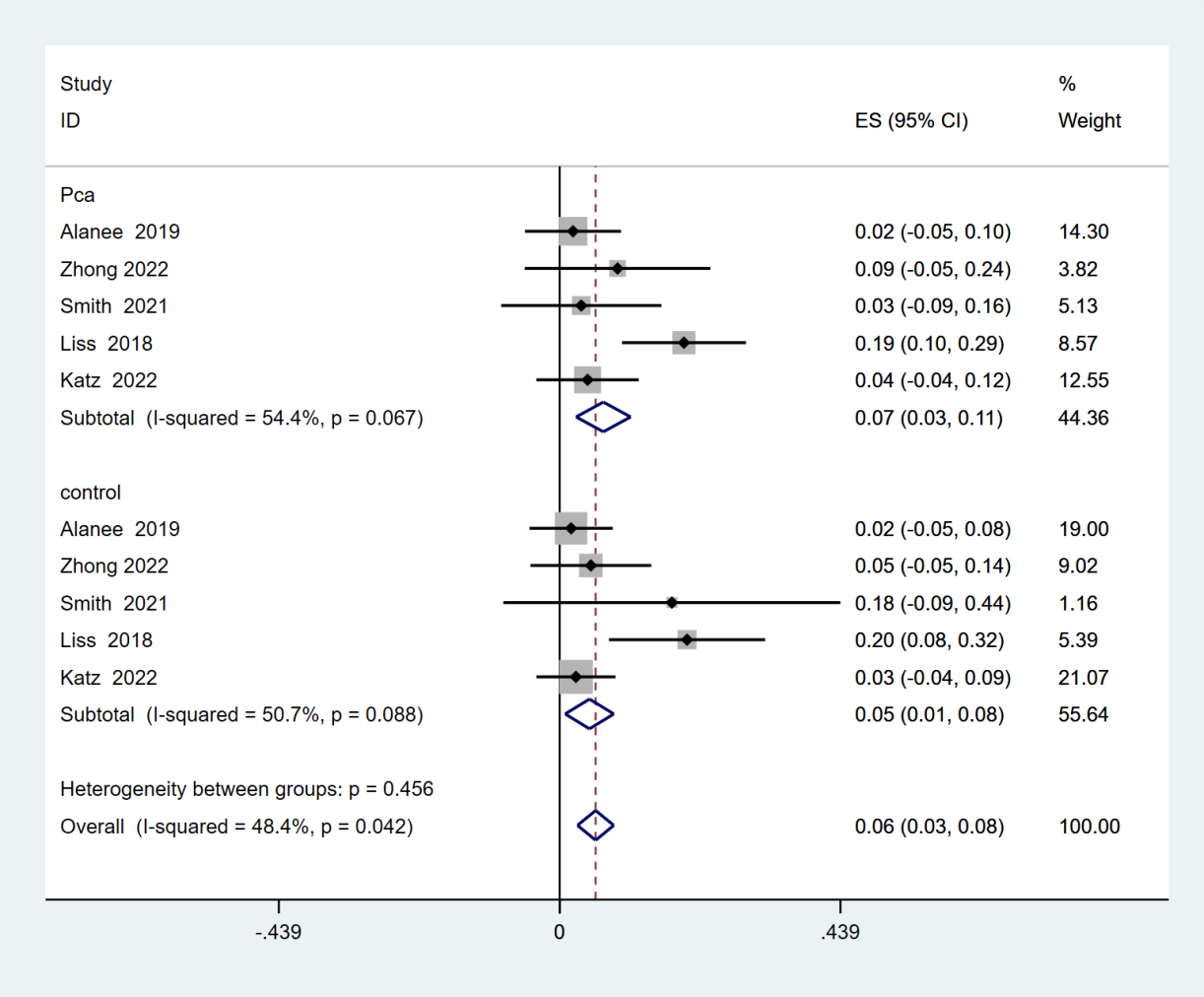


**Figure S10.** Forest plot of relative abundance of *Verrucomicrobia* in prostate patients and controls.


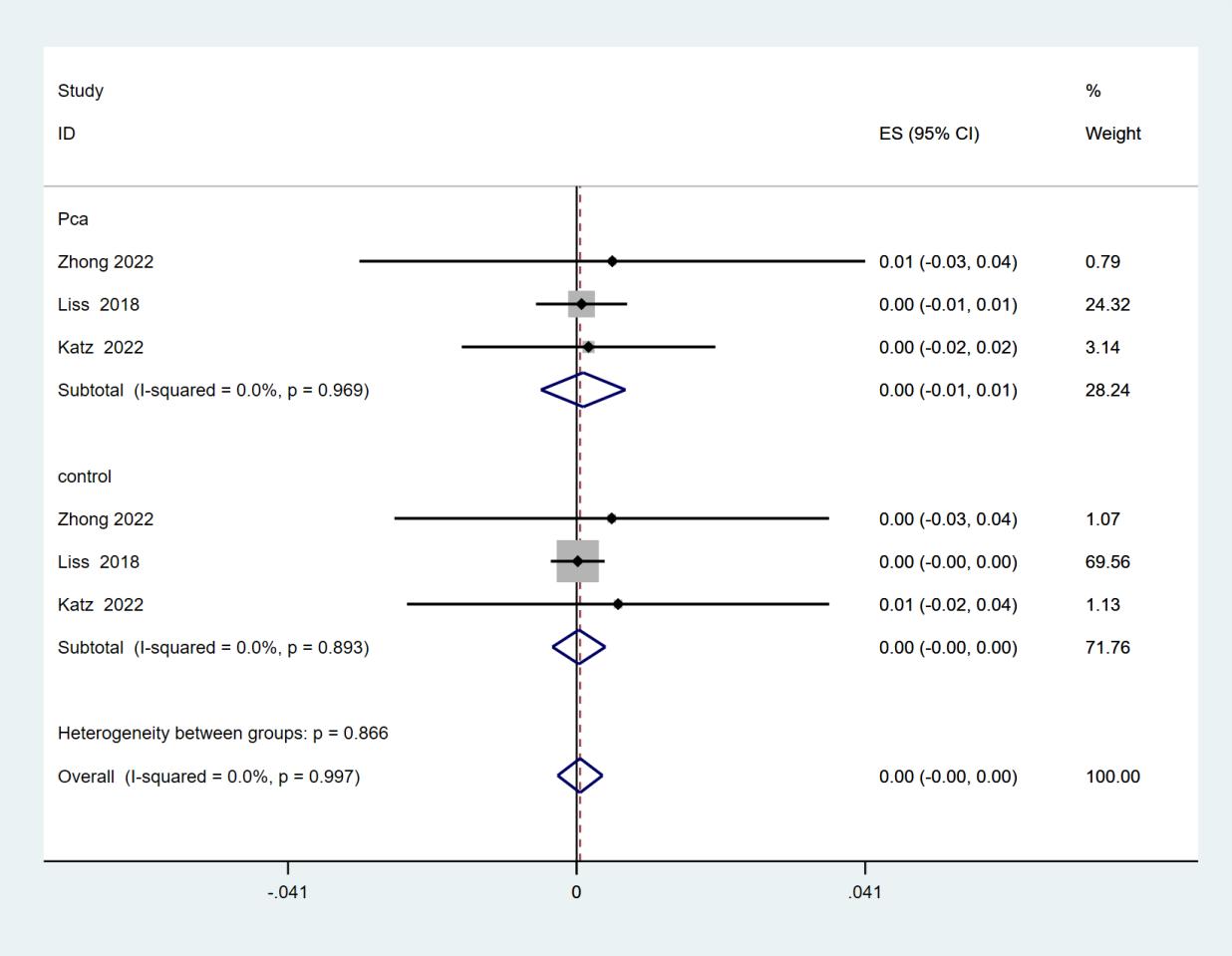


**Figure S11.** Forest plot of relative abundance of *Fusobacteria* in prostate patients and controls.


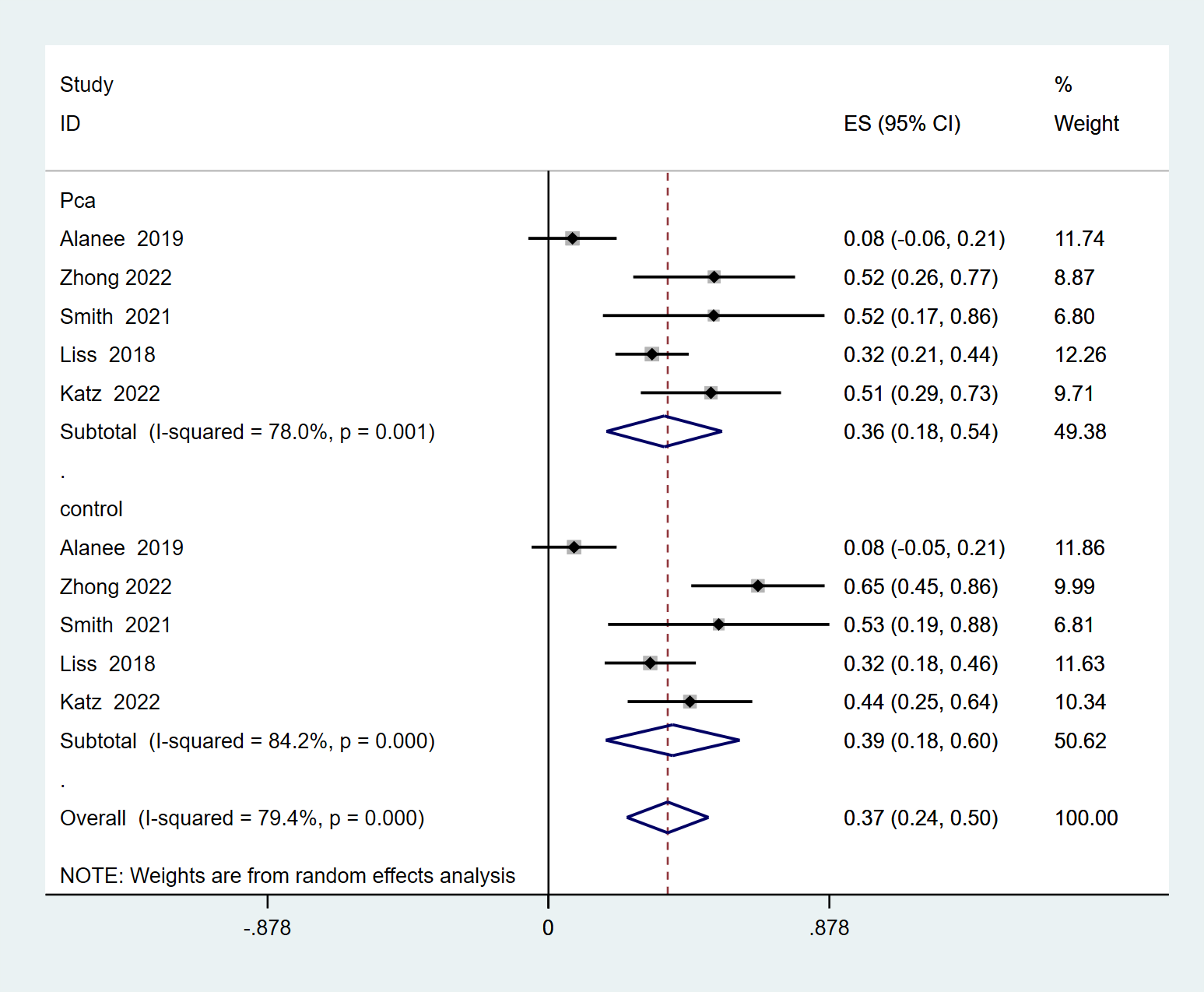


**Figure S12.** Forest plot of relative abundance of *Synergistetes* in prostate patients and controls.


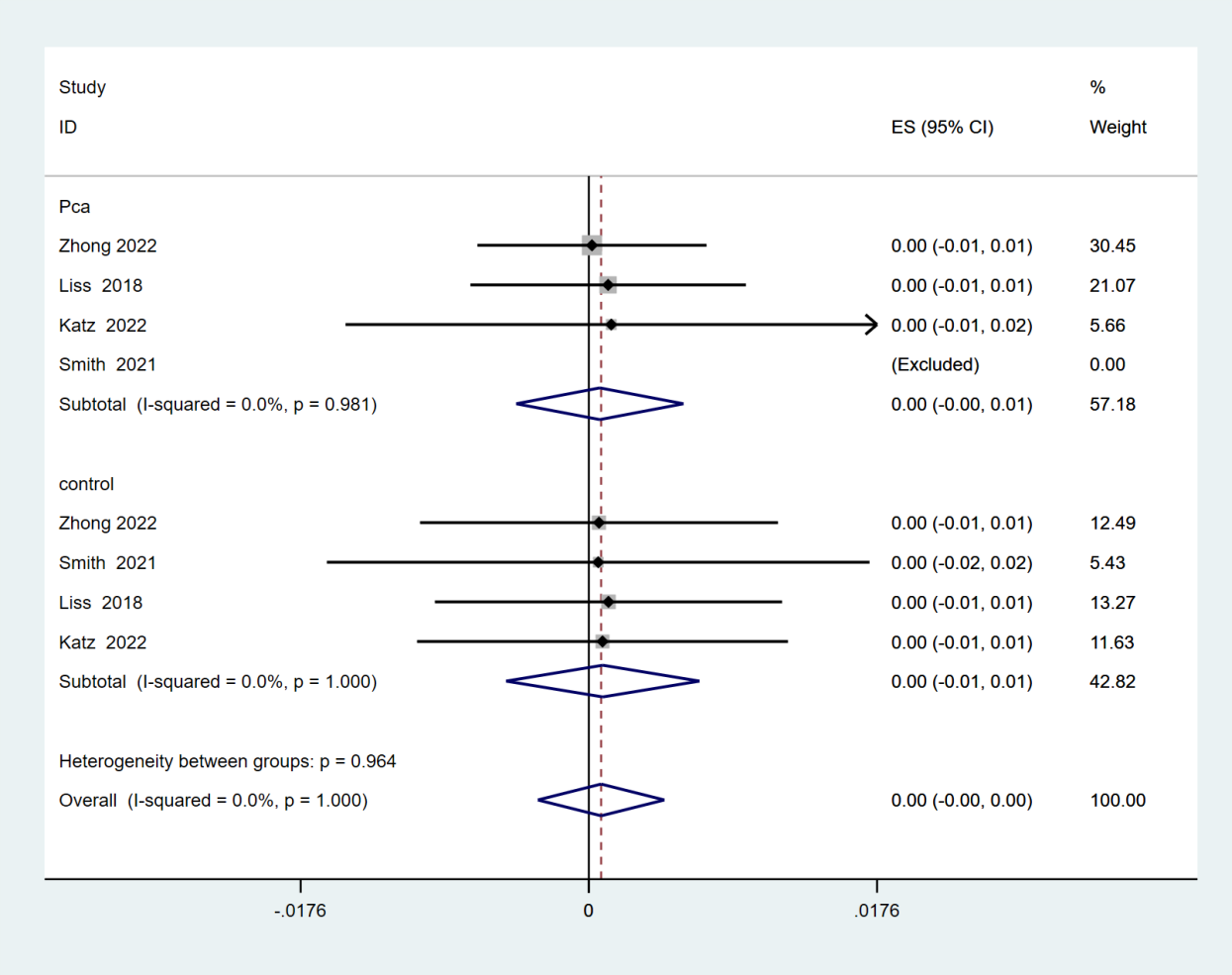


**Figure S13.** Forest plot of relative abundance of *Spirochaetes* in prostate patients and controls.


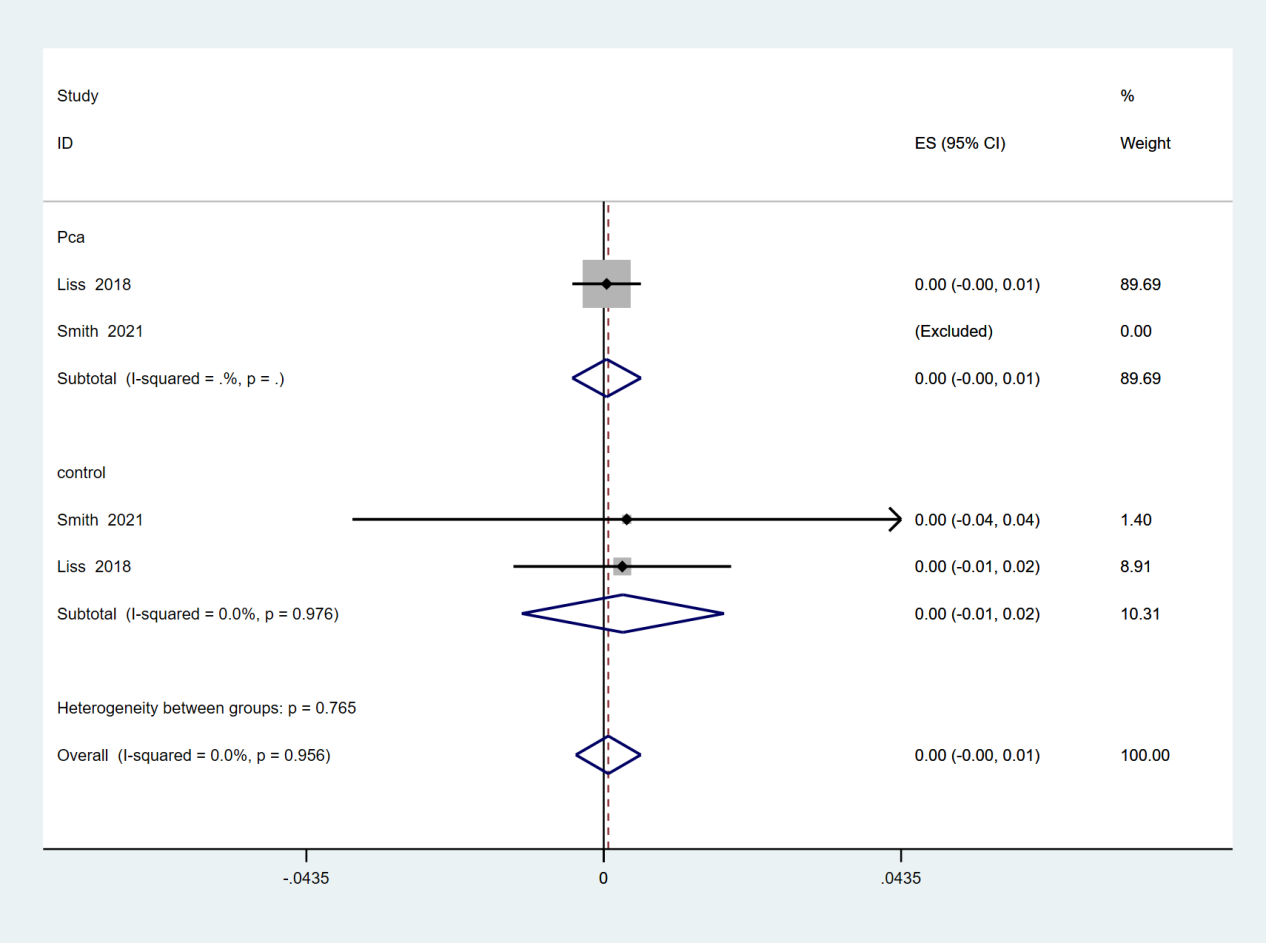

Supplement: Supplementary file 3 — Supplementary Material 3. [file 12885_2024_12018_MOESM3_ESM.zip › Additional file 3/Figure S5-13. Forest plot of relative abundance of GM in at phylum level.docx]
