# Supplementary material for: Gut microbiota in patients with prostate cancer: a systematic review and meta-analysis
Source: BMC Cancer. 2024 Feb 24;24:261. doi: 10.1186/s12885-024-12018-x (PMC10893726; doi:10.1186/s12885-024-12018-x)

**Figure S61.** Forest plot of relative abundance of *Prevotella* in prostate patients and controls.


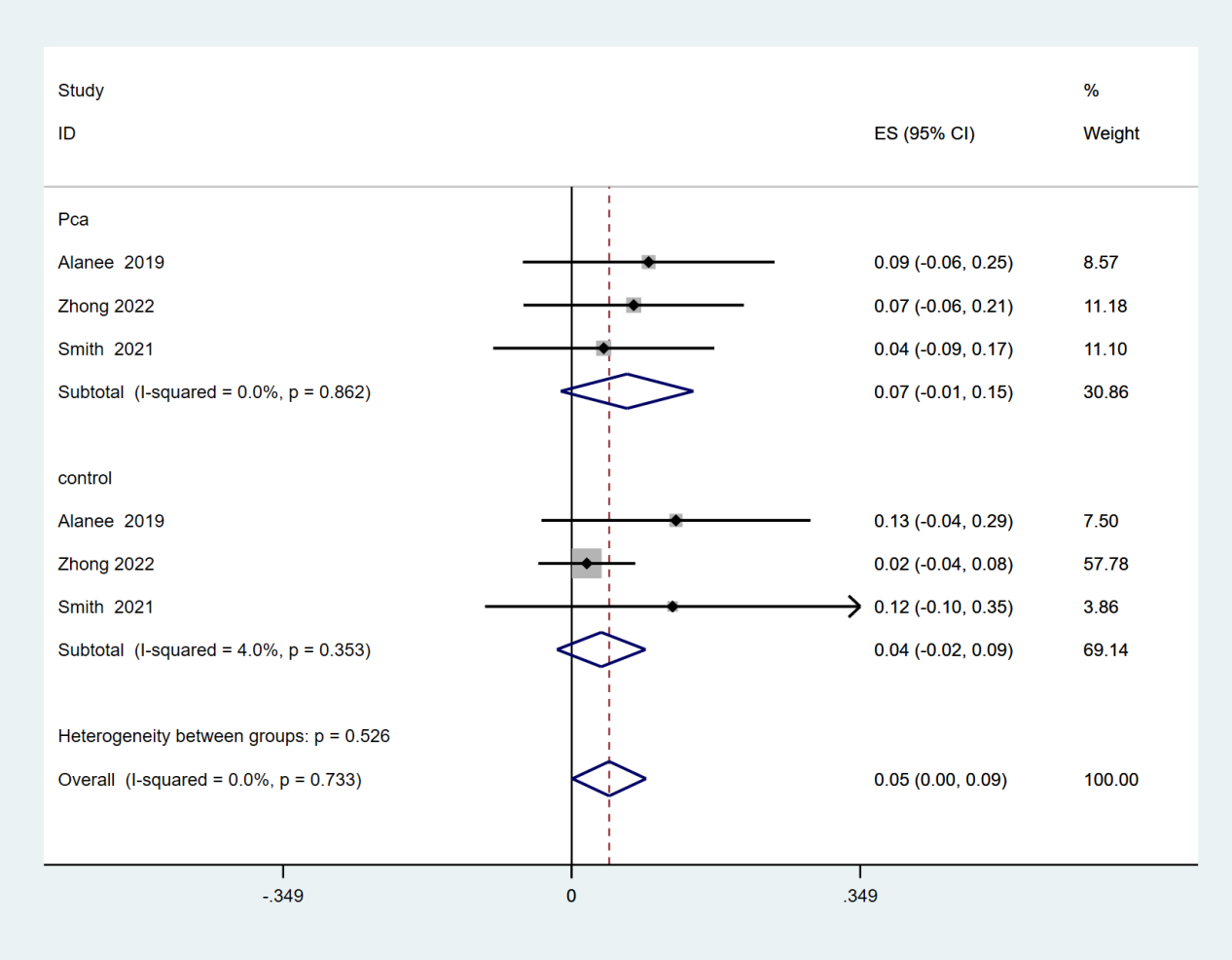


**Figure S62.** Forest plot of relative abundance of *Escherichia -Shigella* in prostate patients and controls.


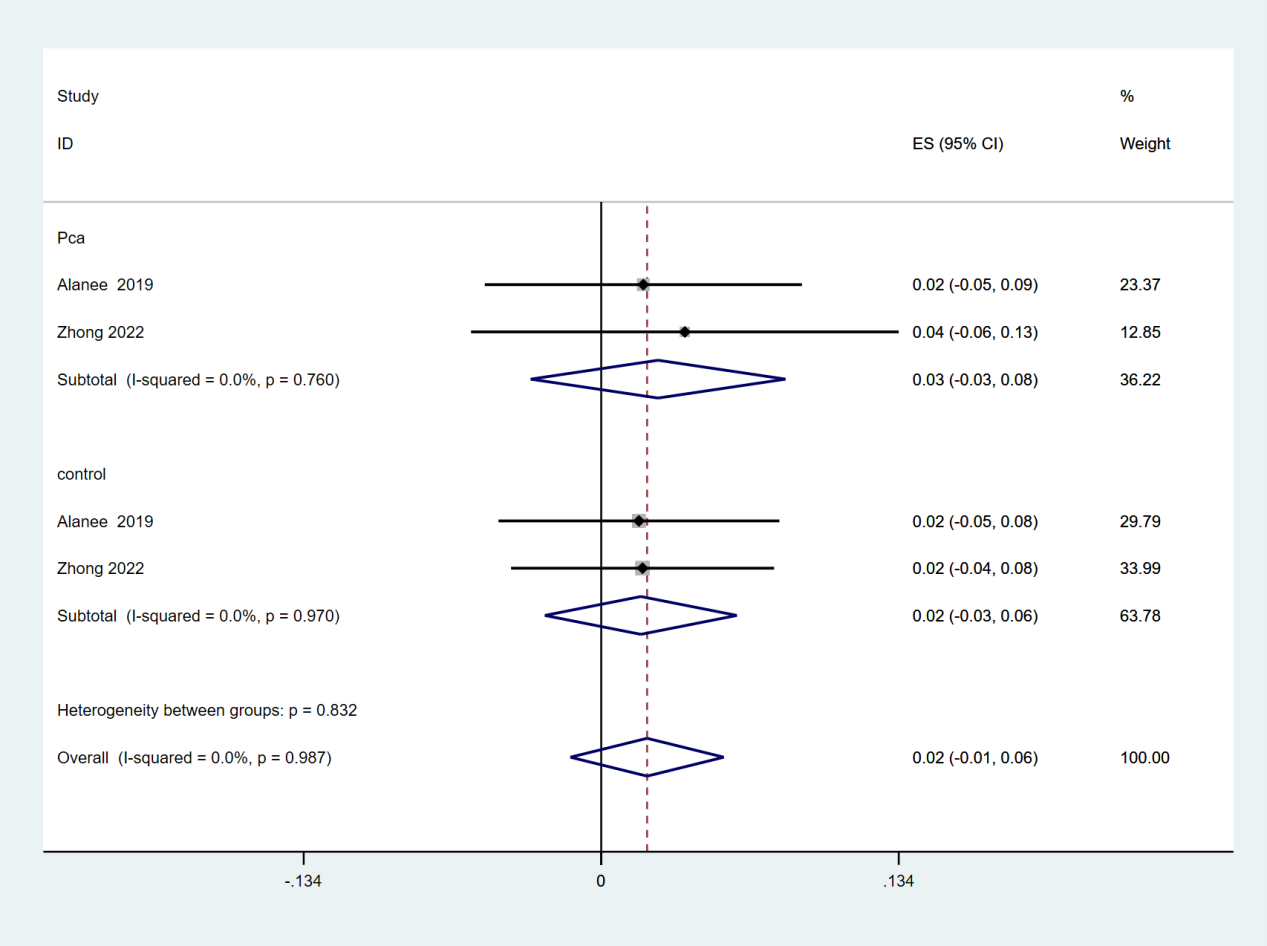


**Figure S63.** Forest plot of relative abundance of *Faecalibacterium* in prostate patients and controls.


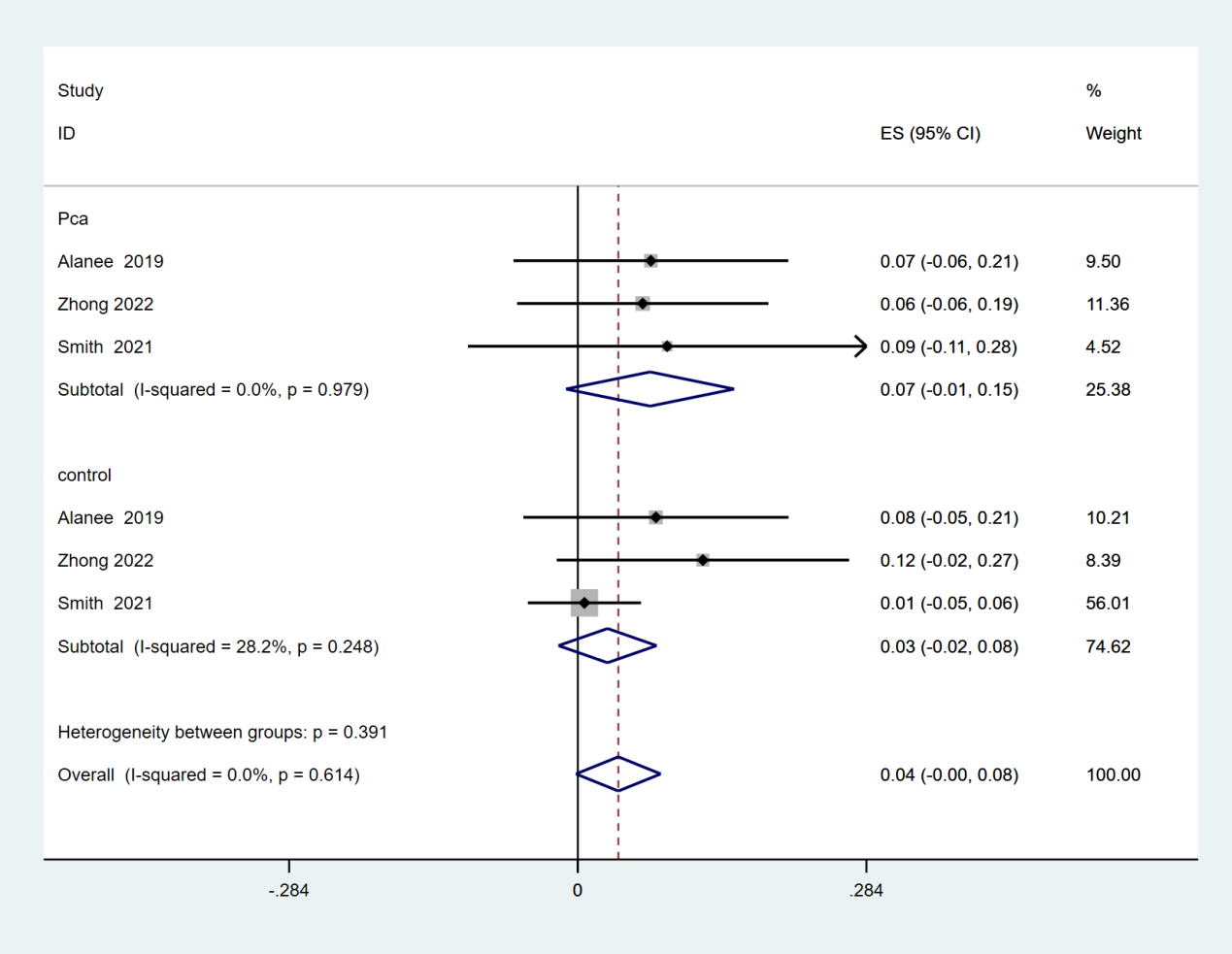


**Figure S64.** Forest plot of relative abundance of *Bacteroides* in prostate patients and controls.


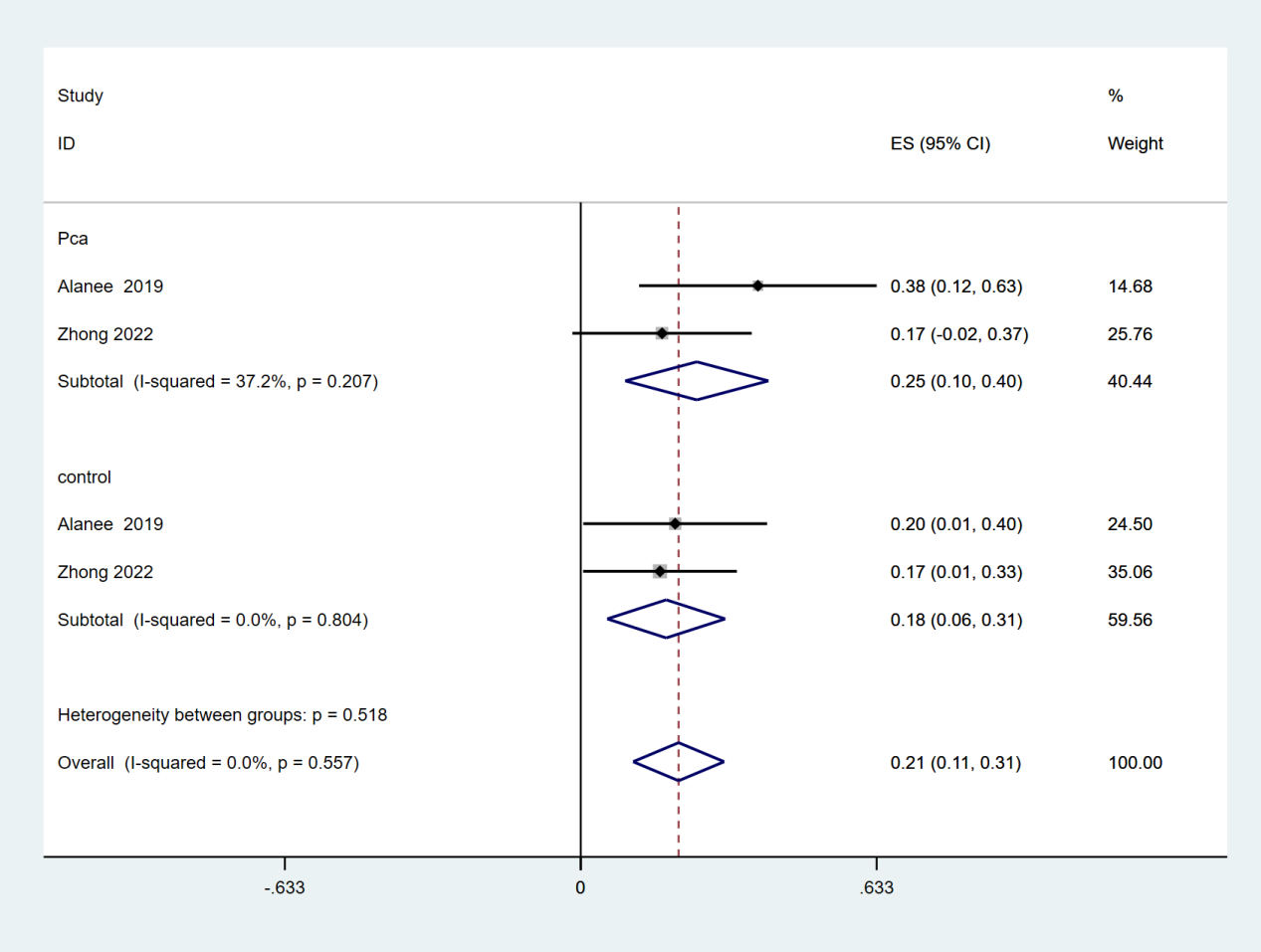


**Figure S65.** Forest plot of relative abundance of *Veillonella* in prostate patients and controls.


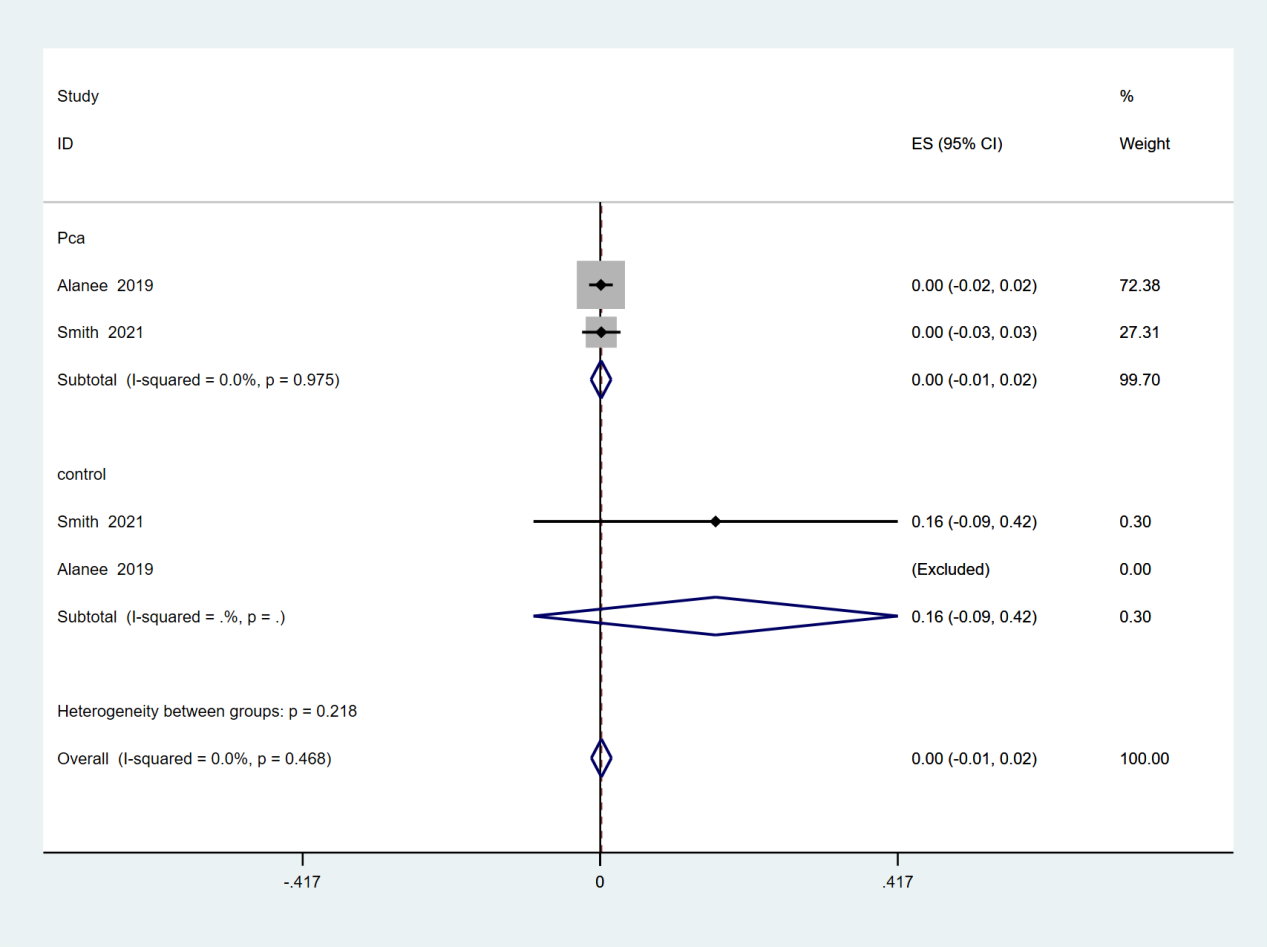


**Figure S66.** Forest plot of relative abundance of *Streptococcus* in prostate patients and controls.


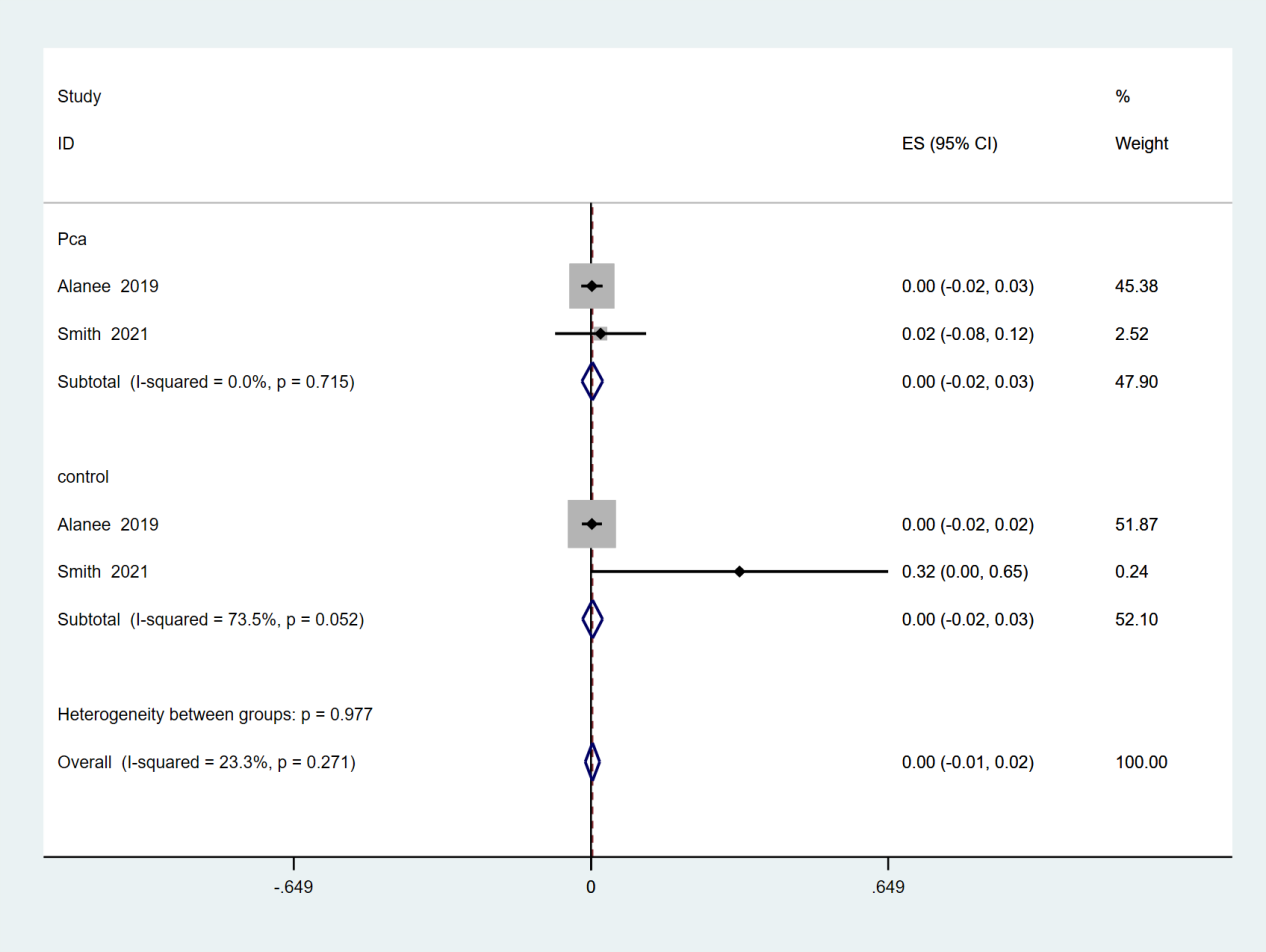


**Figure S67.** Forest plot of relative abundance of *Megasphaera* in prostate patients and controls.


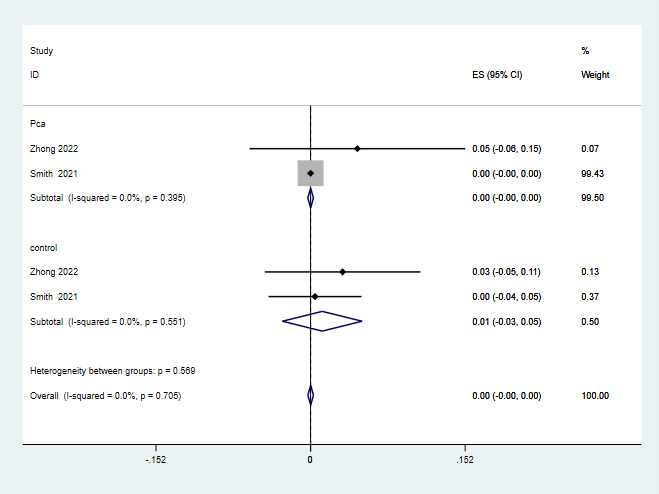

Supplement: Supplementary file 3 — Supplementary Material 3. [file 12885_2024_12018_MOESM3_ESM.zip › Additional file 3/Figure S61-67. Forest plot of relative abundance of GM in at genus level.docx]
