# Supplementary material for: Gut microbiota in patients with prostate cancer: a systematic review and meta-analysis
Source: BMC Cancer. 2024 Feb 24;24:261. doi: 10.1186/s12885-024-12018-x (PMC10893726; doi:10.1186/s12885-024-12018-x)
Supplement: Supplementary file 4 — Supplementary Material 4. [file 12885_2024_12018_MOESM4_ESM.zip › Additional file 4/Table S2. Eggerí»s and Beggí»s tests for publication bias in alpha-diversity.docx]

**Table S2.** *P* value for Egger’s and Begg’s tests for publication bias in alpha-diversity

|  | *P* for Egger’s test | *P* for Begg’s test |
| --- | --- | --- |
| Chao1 | 0.659 | 0.707 |
| Observed Species | 0.084 | 0.089 |
| Shannon | 0.260 | 0.858 |
| Simpson | 0.347 | 0.734 |

No significant publication bias is present when *P*>0.05
