# Supplementary material for: Gut microbiota in patients with prostate cancer: a systematic review and meta-analysis
Source: BMC Cancer. 2024 Feb 24;24:261. doi: 10.1186/s12885-024-12018-x (PMC10893726; doi:10.1186/s12885-024-12018-x)
Supplement: Supplementary file 5 — Supplementary Material 5. [file 12885_2024_12018_MOESM5_ESM.zip › Additional file 5/Table S3-7. Eggerí»s and Beggí»s tests for publication bias in relative abundance of GM.docx]

**Table S3.** *P* value for Egger’s and Begg’s tests for publication bias in GM at the phylum level

|  | *P* for Egger’s test | *P* for Begg’s test |
| --- | --- | --- |
| Actinobacteria | < 0.05 | 0.032 |
| Bacteroidetes | 0.794 | 0.858 |
| Cyanobacteria | 0.308 | 0.048 |
| Firmicutes | 0.044 | 0.210 |
| Proteobacteria | 0.076 | 0.032 |
| Verrucomicrobia | 0.001 | 0.133 |
| Fusobacteria | 0.043 | 0.072 |
| Synergistetes | 0.310 | 1.000 |
| Spirochaetes | 0.289 | 1.000 |

No significant publication bias is present when *P*>0.05

**Table S4.** *P* value for Egger’s and Begg’s tests for publication bias in GM at the class level

|  | *P* for Egger’s test | *P* for Begg’s test |
| --- | --- | --- |
| Actinobacteria | 0.001 | 0.035 |
| Bacteroidia | 0.926 | 0.902 |
| Bacilli | 0.013 | 0.035 |
| Clostridia | 0.228 | 0.386 |
| Erysipelotrichia | 0.276 | 0.707 |
| Negativicutes | 0.002 | 0.221 |
| Gammaproteobacteria | 0.243 | 0.174 |
| Coriobacteriia | 0.004 | 0.308 |
| Deltaproteobacteria | 0.011 | 0.089 |
| Verrucomicrobiae | 0.154 | 0.308 |
| Fusobacteria | 0.244 | 0.308 |
| Alphaproteobacteria | 0.122 | 0.734 |
| Betaproteobacteria | 0.253 | 1.000 |
| Epsilonproteobacteria | 0.239 | 0.308 |
| Synergistia | 0.267 | 0.296 |
| Spirochaetes | 0.289 | 1.000 |

No significant publication bias is present when *P*>0.05

**Table S5.** *P* value for Egger’s and Begg’s tests for publication bias in GM at the order level

|  | *P* for Egger’s test | *P* for Begg’s test |
| --- | --- | --- |
| Bacteroidales | 0.023 | 0.024 |
| Lactobacillales | 0.026 | 0.024 |
| Clostridiales | 0.043 | 0.260 |
| Selenomonadales | 0.102 | 1.000 |
| Enterobacteriales | 0.034 | 0.089 |
| Actinomycetales | 0.050 | 0.308 |
| Bifidobacteriales | 0.001 | 0.296 |
| Coriobacteriales | 0.004 | 0.308 |

No significant publication bias is present when P>0.05

**Table S6.** *P* value for Egger’s and Begg’s tests for publication bias in GM at the famliy level

|  | *P* for Egger’s test | *P* for Begg’s test |
| --- | --- | --- |
| Corynebacteriaceae | 0.040 | 0.308 |
| Prevotellaceae | 0.443 | 0.074 |
| Streptococcaceae | 0.039 | 0.060 |
| Lachnospiraceae | 0.051 | 0.174 |
| Ruminococcaceae | 0.064 | 0.152 |
| Erysipelotrichaceae | 0.077 | 0.308 |
| Acidaminococcaceae | 0.049 | 0.734 |
| Veillonellaceae | 0.005 | 0.016 |
| Burkholderiaceae | 0.916 | 1.000 |
| Enterobacteriaceae | 0.115 | 0.060 |
| Bacteroidaceae | 0.046 | 0.108 |
| Bifidobacteriaceae | 0.002 | 0.221 |
| Actinomycetaceae | 0.051 | 0.308 |

No significant publication bias is present when P>0.05

**Table S7.** *P* value for Egger’s and Begg’s tests for publication bias in GM at the genus level

|  | *P* for Egger’s test | *P* for Begg’s test |
| --- | --- | --- |
| Prevotella | 0.010 | 0.060 |
| Escherichia -Shigella | 0.073 | 0.308 |
| Faecalibacterium | 0.007 | 0.133 |
| Bacteroides | 0.070 | 0.089 |
| Veillonella | 0.201 | 0.296 |
| Streptococcus | 0.124 | 0.089 |
| Megasphaera | 0.097 | 0.089 |

No significant publication bias is present when P>0.05
